# SEC14-GOLD protein PATELLIN2 binds IRON-REGULATED TRANSPORTER1 linking root iron uptake to vitamin E

**DOI:** 10.1093/plphys/kiac563

**Published:** 2022-12-09

**Authors:** Jannik Hornbergs, Karolin Montag, Jennifer Loschwitz, Inga Mohr, Gereon Poschmann, Anika Schnake, Regina Gratz, Tzvetina Brumbarova, Monique Eutebach, Kalina Angrand, Claudia Fink-Straube, Kai Stühler, Jürgen Zeier, Laura Hartmann, Birgit Strodel, Rumen Ivanov, Petra Bauer

**Affiliations:** Institute of Botany, Heinrich Heine University, Düsseldorf 40225, Germany; Institute of Botany, Heinrich Heine University, Düsseldorf 40225, Germany; Institute of Theoretical Chemistry and Computer Chemistry, Heinrich Heine University, Düsseldorf 40225, Germany; Institute of Biological Information Processing (IBI-7: Structural Biochemistry), Forschungszentrum Jülich, 52425 Jülich, Germany; Institute of Botany, Heinrich Heine University, Düsseldorf 40225, Germany; Institute of Molecular Medicine, Proteome Research, Medical Faculty and University Hospital, Heinrich-Heine-University Düsseldorf, Düsseldorf 40225, Germany; Institute for Molecular Ecophysiology of Plants, Heinrich Heine University, Düsseldorf 40225, Germany; Institute of Botany, Heinrich Heine University, Düsseldorf 40225, Germany; Institute of Botany, Heinrich Heine University, Düsseldorf 40225, Germany; Institute of Botany, Heinrich Heine University, Düsseldorf 40225, Germany; Department of Biosciences-Plant Biology, Saarland University, Campus A2.4, D-66123 Saarbrücken, Germany; Leibniz Institute for New Materials, D-66123 Saarbrücken, Germany; Institute of Molecular Medicine, Proteome Research, Medical Faculty and University Hospital, Heinrich-Heine-University Düsseldorf, Düsseldorf 40225, Germany; Molecular Proteomics Laboratory, Heinrich Heine University, Düsseldorf 40225, Germany; Institute for Molecular Ecophysiology of Plants, Heinrich Heine University, Düsseldorf 40225, Germany; Cluster of Excellence on Plant Science (CEPLAS), Heinrich Heine University, Düsseldorf 40225, Germany; Institute of Macromolecular Chemistry, Heinrich Heine University, Düsseldorf 40225, Germany; Institute of Theoretical Chemistry and Computer Chemistry, Heinrich Heine University, Düsseldorf 40225, Germany; Institute of Biological Information Processing (IBI-7: Structural Biochemistry), Forschungszentrum Jülich, 52425 Jülich, Germany; Institute of Botany, Heinrich Heine University, Düsseldorf 40225, Germany; Institute of Botany, Heinrich Heine University, Düsseldorf 40225, Germany; Cluster of Excellence on Plant Science (CEPLAS), Heinrich Heine University, Düsseldorf 40225, Germany

## Abstract

Organisms require micronutrients, and Arabidopsis (*Arabidopsis thaliana*) IRON-REGULATED TRANSPORTER1 (IRT1) is essential for iron (Fe^2+^) acquisition into root cells. Uptake of reactive Fe^2+^ exposes cells to the risk of membrane lipid peroxidation. Surprisingly little is known about how this is avoided. IRT1 activity is controlled by an intracellular variable region (IRT1vr) that acts as a regulatory protein interaction platform. Here, we describe that IRT1vr interacted with peripheral plasma membrane SEC14-Golgi dynamics (SEC14-GOLD) protein PATELLIN2 (PATL2). SEC14 proteins bind lipophilic substrates and transport or present them at the membrane. To date, no direct roles have been attributed to SEC14 proteins in Fe import. PATL2 affected root Fe acquisition responses, interacted with ROS response proteins in roots, and alleviated root lipid peroxidation. PATL2 had high affinity in vitro for the major lipophilic antioxidant vitamin E compound α-tocopherol. Molecular dynamics simulations provided insight into energetic constraints and the orientation and stability of the PATL2-ligand interaction in atomic detail. Hence, this work highlights a compelling mechanism connecting vitamin E with root metal ion transport at the plasma membrane with the participation of an IRT1-interacting and α-tocopherol-binding SEC14 protein.

## Teaser

The interaction between a plasma membrane root iron transporter and a SEC14-GOLD peripheral membrane protein is critical for root iron mobilization and antioxidant activities in response to iron.

## Introduction

Essential trace elements are crucial for metabolic processes, stress responses, and combatting disease, such as photosynthesis in plants, oxygen transport or lowering cancer risk in humans. Micronutrients are often of limited access for humans (Global Nutrition Report, 2021) and for plants (Briat et al., 2015). ZINC- and IRON-REGULATED TRANSPORTERs (ZRTs and IRTs, respectively, ZRT- and IRT-LIKE PROTEINS, ZIPs) represent an evolutionarily conserved family of membrane transporters for achieving divalent metal ion uptake into cells (Guerinot, 2000; Hu, 2021), with Arabidopsis (*Arabidopsis thaliana*) IRT1 being a founding member of these affinity transporters (Eide et al., 1996; Zhao and Eide, 1996).

IRT1 is essential for taking up soil Fe (Eide et al., 1996; Henriques et al., 2002; Vert et al., 2002), and it has emerged as a model for studying the regulation of divalent metal ion transport across the plasma membrane (Ivanov and Vert, 2021). Plants control IRT1 activity tightly because this transporter has a broad specificity for reactive metal ions (Korshunova et al., 1999; Connolly et al., 2002). These reactive substrates and cytotoxic Fenton reagents, like Fe^2+^, catalyze the generation of ROS-derived radicals resulting in oxidative stress (Le et al., 2016; Dubeaux et al., 2018; Le et al., 2019; Gratz et al., 2021; Juan et al., 2021; von der Mark et al., 2021). The large cytoplasmic IRT1 loop between transmembrane helices three and four, also known as a variable region of IRT1 (Guerinot, 2000) or briefly IRT1vr, plays a crucial role in IRT1 control. Interaction with the ubiquitin ligase IDF1 causes mono-ubiquitination of IRT1vr and endocytosis of IRT1 (Shin et al., 2013). Endocytosed IRT1 may be recycled with the help of complexes containing peripheral membrane proteins (Barberon et al., 2014; Ivanov et al., 2014; Brumbarova and Ivanov, 2016). IRT1vr consists of membrane-proximal regions separated by an intrinsically disordered part (Khan et al., 2019). A calcium-requiring C2-domain protein, namely ENHANCED BENDING1 (EHB1), binds to the membrane-proximal regions of IRT1vr and inhibits Fe uptake (Khan et al., 2019). The disordered IRT1vr subdomain has a functional histidine-rich metal-ion-binding site. When Mn^2+^ and Zn^2+^ are present in excess, a calcium-responsive protein kinase action is triggered, followed by IRT1 polyubiquitination, endocytosis, and vacuolar degradation of IRT1 (Dubeaux et al., 2018). Thus, IRT1vr represents a platform for recruiting regulatory protein complexes to steer the uptake of reactive divalent metal ions. Even more yet unknown protein–protein interactions may control IRT1 to avoid deleterious effects of metal ion uptake.

Fe^2+^ facilitates lipid peroxidation of polyunsaturated fatty acids in the membrane and their breakdown through cycles of lipid radical formation (Juan et al., 2021). Tocopherols and related tocotrienols, a group of lipophilic antioxidant substances, collectively termed “vitamin E,” counteract this effect (Mène-Saffrané, 2018). Vitamin E compounds are antioxidants to protect membrane lipid identity during photosynthesis, energy storage, or during plant stress responses (Mène-Saffrané, 2018). The relation of polyunsaturated fatty acids and vitamin E compounds also represents an important determinant for disease risks in humans, e.g. in cancer prevention, emphasizing the general role played by vitamin E in stress reduction (Sylvester, 2019; Zingg and Meydani, 2019).

Lipid transfer proteins play fundamental roles in the homeostasis of vitamin E in humans (Atkinson et al., 2019), and one class is represented by the SEC14-like phosphatidylinositol transfer protein (SEC14L-PITP) superfamily, also present in plants (de Campos and Schaaf, 2017; Wong et al., 2017; Montag et al., 2020). The characteristic SEC14 domain provides a lipid-binding pocket to transfer single lipophilic substrates between membranes, exchange them in the membrane, or present them as substrates to enzymes in the membrane (de Campos and Schaaf, 2017). Golgi dynamics (GOLD) domain-containing SEC14 PITPs mediate protein–lipid and protein–protein interactions in complex multicellular organisms (Anantharaman and Aravind, 2002; Carney and Bowen, 2004; Saito et al., 2007; Montag et al., 2020). SEC14L-GOLD-PITPs are known as PATELLIN (PATL) proteins in land plants (Peterman et al., 2004). They acquired partly different features compared to animal counterparts. For example, the SEC14 domain enables membrane association (Peterman et al., 2004; Montag et al., 2020), while the GOLD domain of PATL2 confers specificity for associating particularly with phosphoinositides (PIs) (different types of phosphatidylinositol phosphates, PIPs) of the plasma membrane (Montag et al., 2020). PATLs localize at the cell plate (*patella*) during cell division (Peterman et al., 2004; Suzuki et al., 2016; Wu et al., 2017; Tejos et al., 2018). The six Arabidopsis PATLs have overlapping and redundant roles in cell polarity but also specific functions in stress responses (Peterman et al., 2004; Suzuki et al., 2016; Rutter and Innes, 2017; Wu et al., 2017; Chu et al., 2018; Tejos et al., 2018; Zhou et al., 2018; Montag et al., 2020). PATL1 acts upon salt treatment and freezing (Chu et al., 2018; Zhou et al., 2018). Interestingly, phosphopeptides derived from PATL2 were detected in Fe starvation experiments (Lan et al., 2012) and under salt stress in membrane fractions (Hsu et al., 2009; Chang et al., 2012). These observations suggest that PATLs may play yet undescribed roles in plant stress resilience. So far, little is known about the cellular functions of plant PATLs in a stress physiological context and their lipophilic substrate-binding characteristics.

We retrieved the SEC14-GOLD PITP PATL2 in a screen for IRT1vr-regulatory proteins from Fe-deficient roots. A role of PATL2 during nutrient and Fe uptake had not been known. We show that PATL2 affects Fe acquisition responses. We provide evidence that suggests PATL2 to be part of an operational system that prevents lipid peroxidation in roots through binding the lipophilic antioxidant α-tocopherol. Hence, this work highlights participation of the IRT1-interacting SEC14 protein PATL2 in redox control during Fe^2+^ transport.

## Results

### IRT1 can interact through IRT1vr with the N terminus of PATL2 in root epidermis cells of the root differentiation zone where IRT1 and PATL2 proteins are expressed

**Figure 1:**
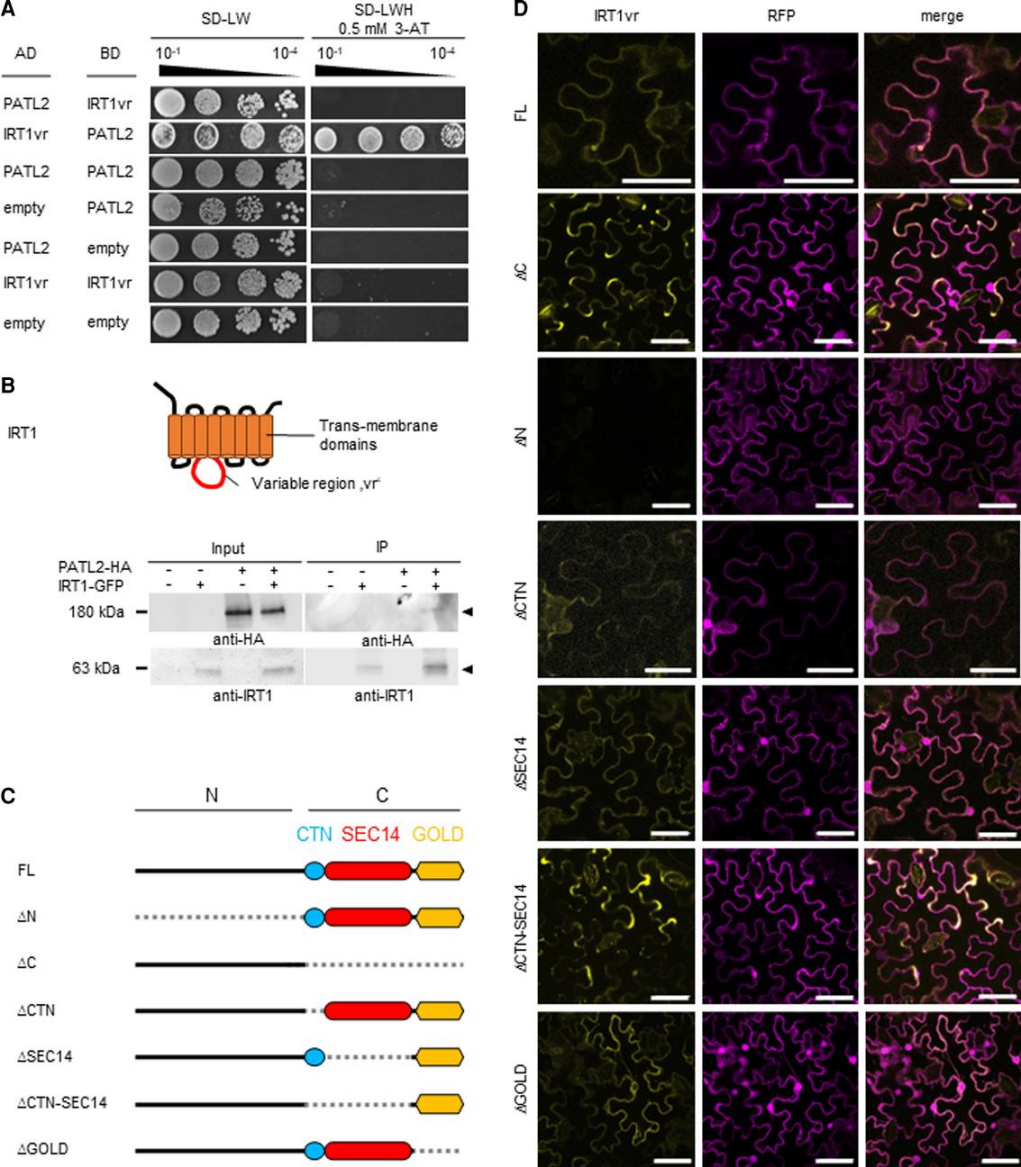
The N-terminal region of PATELLIN2 (PATL2) interacted with the variable region and large cytoplasmic loop of IRON-REGULATED TRANSPORTER1 (IRT1). A, Targeted yeast-two-hybrid assay validating the interaction of PATL2 with the large intracellular loop, also known as “variable region” of IRT1, IRT1vr. Above, photos of yeast colony growth; yeast cells spotted in dilutions as indicated from 10^−1^ to 10^−4^; right, selection medium (SD-LW), growth indicates protein–protein interaction; left, non-selection medium (SD-LWH), growth serves as positive control. AD, activation domain; BD, binding domain; SD, synthetic dropout medium; L, leucine; W, tryptophane; H, histidine. Below, scheme of the IRT1 structure, in red IRT1vr. B, Plant cell co-immunoprecipitation (IP) analysis, demonstrating IP of triple hemagglutinin (HA_3_)-tagged PATL2-HA_3_ along with IRT1-GFP from plant protein extracts (anti-GFP beads). Protein immunoblot detection before (input) and after IP, in the presence (+) or absence (−) of proteins as indicated; arrowheads, sizes of detected proteins. IRT1-GFP had previously been localized at the plasma membrane and used for Co-IP studies to pull down IRT1-interacting HA_3_-ENHANCED BENDING1 (EHB1), but not a non-interacting plasma membrane-associated mutant version of EHB1 (Khan et al., 2019). Functional complementation data are not available for the IRT1-GFP construct. The PATL2-HA_3_ construct functionally complemented a *patl2* mutant, as described in a later section of this work. See also images in Supplemental Materials and Methods. C, Schematic representation of PATL2 full-length (FL) and various mutants with deletions (Δ, indicated by dashed lines) of the N- and C-terminal parts (N, C), the CRAL-TRIO-N-terminal extension (CTN), the SEC14, CTN-SEC14, and Golgi dynamics (GOLD) domains, used in (D) and generated according to Montag et al. (2020). D, Plant cell BiFC of split Yellow fluorescent protein (YFP) between PATL2 full-length (FL) and its deletion variants depicted in (C) fused N-terminally with nYFP and IRT1vr fused N-terminally with cYFP. Note that localization of reconstituted YFP corresponds to previously determined localization of YFP-PATL2 and respective deletion mutants in plant cells (Montag et al., 2020). YFP signal, indicating protein–protein interaction; Red fluorescent protein (RFP) signal, positive plant cell transformation control; merge, overlay of YFP and RFP signals. B, D, Plant cells are transiently transformed *N. benthamiana leaf* epidermis cells. Size bars: 50 *µ*m.

Identifying the IRT1vr protein interaction environment is an important step in the understanding of the cellular regulation of this divalent metal transporter. Screening a cDNA expression library prepared from Fe-deficient Arabidopsis roots has been successful to unravel the signaling inputs involved in the regulation of IRT1 via IRT1vr (Khan et al., 2019). In this screen, we found that the peripheral membrane protein PATL2 was among identified putative interactors. PATL2-IRT1vr protein interaction was subsequently reconfirmed in a targeted Y2H assay with the combination of AD-IRT1vr and BD-PATL2 (Figure 1A). The combination AD-PATL2 and BD-IRT1vr did not result in yeast growth and protein interaction, perhaps caused by steric hindrance affecting the yeast growth assay. Green fluorescent protein (GFP)-tagged IRT1-GFP protein was co-immunoprecipitated together with triple hemagglutinin (HA_3_)-tagged PATL2-HA_3_ protein using transient expression of *Nicotiana benthamiana* epidermis leaf cells (Figure 1B). Previous microscopic experiments have shown that IRT1-GFP localized to the plasma membrane in these leaf cells, as was also the case for YFP-PATL2 (Khan et al., 2019; Montag et al., 2020). IRT1-GFP did not un-specifically interact with the HA_3_ tag or co-precipitate with non-interacting membrane proteins in previous co-immunoprecipitation experiments (Khan et al., 2019). Hence, the data suggest that IRT1-GFP interacted with PATL2-HA_3_ in plant cells.

Next, we pinpointed the PATL2 domain responsible for interaction with IRT1vr using a set of previously described PATL2 deletion constructs (Montag et al., 2020) in a plant cell YFP bimolecular fluorescence complementation (BiFC) experiment in transiently transformed *N. benthamiana* leaf epidermis cells. We found the reconstituted split YFP signals indicative of protein–protein interactions when IRT1vr-cYFP was combined together with full-length PATL2-nYFP (FL). In PATL2, the SEC14 domain (= SEC14), sometimes termed CRAL-TRIO domain (Novick et al., 1980), is preceded by a N-terminally located CRAL-TRIO domain extension (= CTN) (Montag et al., 2020). The GOLD domain (= GOLD) is present at the C terminus in PATL2. Protein interaction was also found for the protein pair IRT1vr-cYFP and PATL2ΔC-nYFP (ΔC), lacking the entire CTN-SEC14-GOLD domain (Figure 1, C and D). Furthermore, protein interaction was detected for IRT1vr-cYFP together with four deletion variants lacking individual C-terminal domains, namely PATL2ΔCTN-nYFP (ΔCTN), PATL2ΔSEC14-nYFP (ΔSEC14), PATL2ΔCTN-SEC14-nYFP (ΔCTN-SEC14), and PATL2ΔGOLD-nYFP (ΔGOLD) (Figure 1, C and D). In all these cases, YFP signals were detected at a membrane or in the cytoplasm in the expected localization patterns according to previous localization of GFP-tagged PATL2 and respective deletion forms (Montag et al., 2020) (Figure 1, C and D). No YFP signals were detected for the combination of IRT1vr-cYFP and PATL2ΔN-nYFP (ΔN) lacking the N terminus (Figure 1, C and D). Previously, YFP-PATL2ΔN was stably expressed and located at the plasma membrane (Montag et al., 2020). Moreover, the PATL2ΔN variant protein had previously been successfully produced in *Escherichia coli* and found to bind phospholipids (Montag et al., 2020). Many SEC14-GOLD proteins share a structure similar to PATL2ΔN devoid of a N region (Montag et al., 2020). Indeed, PATL2ΔC consisting of only the N part led to YFP complementation with IRT1vr, as described above. Therefore, these results indicate that PATL2 interacts via its N-terminal region with IRT1vr.

IRT1-mCherry and YFP-PATL2 fluorescent fusion proteins had both been localized at the plasma membrane in transiently transformed *N. benthamiana* leaf epidermis cells (Khan et al., 2019; Montag et al., 2020). We found indeed co-localization of these two fusion proteins at the plasma membrane when they were co-expressed in this system (Supplemental Figure 1, A and B).

GFP-PATL2 and IRT1-mCitrine had been previously located at the plasma membrane in root epidermis cells (Dubeaux et al., 2018; Tejos et al., 2018). When Arabidopsis seedlings containing proPATL2::GFP-PATL2 (Tejos et al., 2018) were cultivated in our growth conditions in the 6-day system at 0 *µ*M Fe, GFP-PATL2 signals were localized at the plasma membrane in root epidermis cells of the root differentiation zone, where Fe acquisition takes place (Supplemental Figure 1C). Upon application of FM-64, co-localization at the plasma membrane was visible. FM-64 was rapidly internalized by root hair cells and then localized in vesicles in the cytoplasm, well revealing the position of the vacuole. It can be seen that GFP-PATL2 was at the plasma membrane but not at the tonoplast (Supplemental Figure 1D). Additionally, there were strong GFP-PATL2 signals in the central root tissues (Supplemental Figure 1C). We grew proPATL2::GFP-PATL2 seedlings alongside proIRT1::IRT1-mCitrine/*irt1-1* seedlings in the 6-day system at 0 *µ*M Fe and compared the protein localization patterns in more detail in the root differentiation zone (Supplemental Figure 1, D and E). The analysis showed that GFP-PATL2 and IRT1-mCitrine signals were both detectable under 0 *µ*M Fe supply in the root differentiation zone. Signals were both present in the epidermis and in root hair cells. A difference was that IRT1-mCitrine signals were clearly weaker than GFP-PATL2 signals, whereby strongest IRT1-mCitrine signals were present in root hair cells. GFP-PATL2 and IRT1-mCitrine signals were present at the plasma membrane. Again, differences were noted with regard to the patterns of localization inside the root epidermis cells. GFP-PATL2 signals were uniformly distributed at the plasma membrane, which was not the case for IRT1-mCitrine signals. Some parts of the plasma membrane were devoid of IRT1-mCitrine signal, which may reflect either the described “polar localization” pattern of IRT1-mCitrine (Dubeaux et al., 2018) or a response to positioning of plant roots on the agar medium. Furthermore, IRT1-mCitrine signals were strong in several small foci of the plasma membrane and in the cytoplasm, which was likely a result of the described regulation of IRT1-mCitrine by endocytosis and its presence in vesicles (Dubeaux et al., 2018). Taken together, these results suggest that PATL2 and IRT1 can colocalize at the plasma membrane in the epidermis of the root differentiation zone.

The expression in the same root epidermis cells of the root differentiation zone was also seen in studies of the *PATL2* and *IRT1* promoter activities driving a GUS reporter, which overlapped in the root epidermis and root hair cells under Fe supply (50 *µ*M Fe) and Fe deficiency (0 *µ*M Fe) in our growth system (Supplemental Figure 2). Beyond that, the *PATL2* promoter was active throughout plant development and in several tissues of the root, as expected (Tejos et al., 2018; Supplemental Figure 2A).

Hence, IRT1 and PATL2 can interact at the plasma membrane in root epidermis cells.

### The most drastic and consistent Fe deficiency response phenotype of *patl2* mutants was observed at the level of Fe reductase activity

It had not been known whether PATL2 affects Fe acquisition responses. To clarify the importance of PATL2 in Fe uptake, we studied Fe deficiency response phenotypes of two loss-of-function mutant alleles of PATL2, *patl2-1* and *patl2-2* (Supplemental Figure 2).

**Figure 2:**
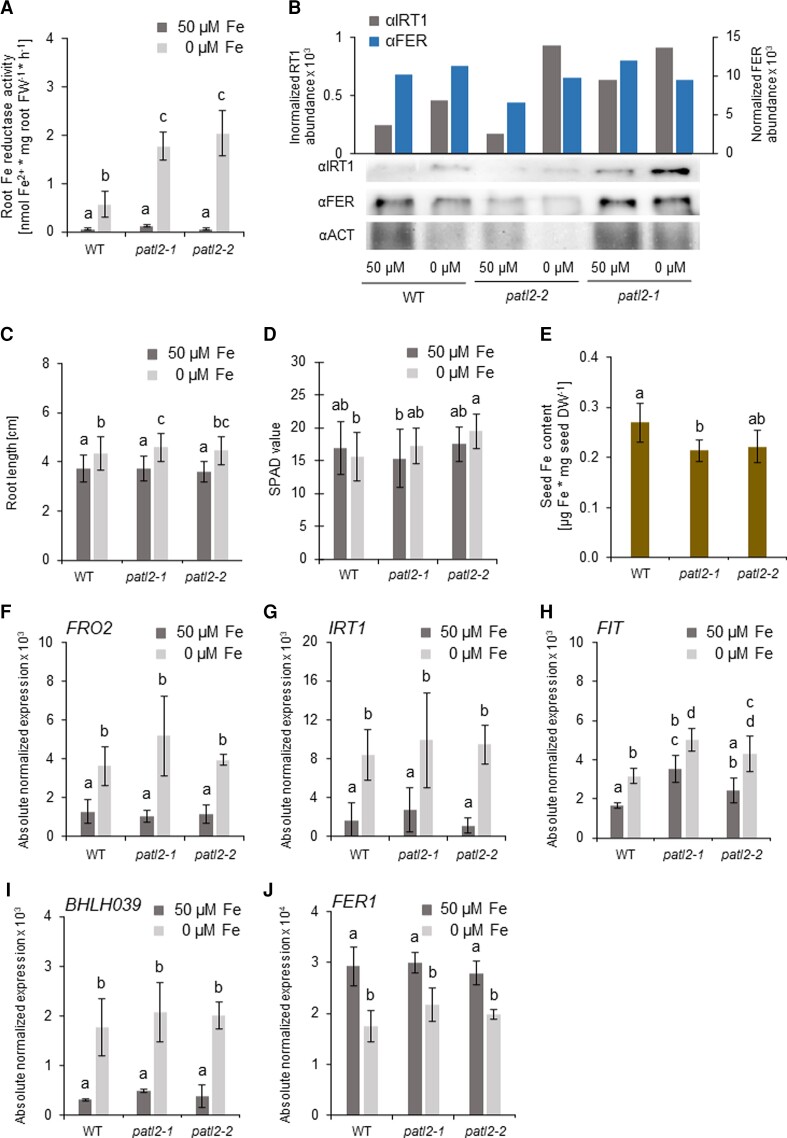
Enhanced Fe reductase activity was the most drastic and consistent phenotype of *patellin2* (*patl2*) loss of function mutants. Physiological and molecular analysis of *patl2* mutant plants (additional allele description in Supplemental Figure 3, A–G). A, Root Fe reductase activity. Plants were grown in the 14 + 3 system. *n* = 3 (B) IRON-REGULATED TRANSPORTER1 (IRT1) and ferritin (FER) immunoblot analysis with anti-IRT1, anti-FER and anti-ACTIN, as indicated. Top, actin-normalized band signal intensities; bottom, immunoblot bands after chemiluminescent signal detection. See also images in Supplemental Materials and Methods. Plants were grown in the 14 + 3 system. C, Root length measurements. Plants were grown in the 10-day system. *n* = 83. D, Leaf SPAD values. Plants were grown in the 14 + 3 system. *n* = 18. E, Fe contents per seed dry weight, harvested from soil-grown plants. *n* = 3. F, J, Root gene expression of Fe response markers. Plants were grown in the 14 + 3 system. *n* = 3. F–I, Fe deficiency markers (F) *FERRIC REDUCTASE OXIDASE2* (*FRO2*), (G) *IRT1*, (H) *FER-LIKE FE DEFICIENCY-INDUCED TRANSCRIPTION FACTOR* (*FIT*), (I) *BASIC HELIX-LOOP-HELIX039* (*BHLH039*), and (J) Fe sufficiency marker *FERRITIN1* (*FER1*). A–J, Wild type (WT), *patl2-1* and *patl2-2* plants were grown as indicated and exposed to Fe sufficiency (50 *µ*M Fe) and Fe deficiency (0 *µ*M Fe). Data in (A, C–J) are represented as mean ± standard deviations. Different letters indicate statistically significant differences (*P* < 0.05, determined by ANOVA with post hoc Fisher's LSD test).

When exposing *patl2* mutants to 0 and 50 *µ*M Fe, we found that the most drastic and consistent phenotype was observed at the level of Fe deficiency-induced root Fe reductase activity. Root ferric reductase activity is an indicator for Fe mobilization activity along the root (Robinson et al., 1999). Root ferric reductase activity was strikingly up-regulated two-to-four-fold at 0 *µ*M Fe in the two single *patl2* mutants compared to the wild type, and similarly in a double *patl1 patl2* mutant, but not in a single *patl1* mutant (Figure 2A; see also in further information on *PATL1*, Supplemental Figures 3–5).

In contrast to Fe reductase activity data, the analysis of other Fe deficiency responses indicated that *patl* mutants were either not affected or had mild or inconsistent phenotypes. IRT1 and ferritin (FER) protein abundances are markers for Fe deficiency and sufficiency in roots (Eide et al., 1996; Ravet et al., 2009). The increase in IRT1 protein abundance at 0 *µ*M Fe compared with 50 *µ*M Fe was similar in *patl2-1* and wild type and higher in *patl2-2* than wild type (Figure 2B). There was a mild Fe deficiency-induced root elongation of seedlings, a typical seedling response in our growth system (Kanwar et al., 2021) in *patl2-1* which was only 10% different compared with wild type (Figure 2C). Differences in leaf soil plant analysis development (SPAD) values, indicative of leaf chlorosis, were not noted in *patl2* mutants versus wild type (Figure 2D). The seed Fe contents were either not changed or reduced by 30% in *patl2-1* and 40% in *patl1-1* but not *patl2-2* or the double mutant, compared with wild type (Figure 2E; Supplemental Figure 5, C and D). At the level of root Fe deficiency response gene expression patterns, no difference was seen with regard to *FRO2* and *IRT1* gene expression (Figure 2, F and G, Supplemental Figure 5, E and F). *FIT* and *BHLH039* encode the transcription factors that target *FRO2* and *IRT1* (Yuan et al., 2008; Sivitz et al., 2012; Naranjo Arcos et al., 2017; Gratz et al., 2019a, 2019b). *FIT* was upregulated by 50% and 40% in the *patl2* mutants compared to wild type at 0 *µ*M (Figure 2H) but expressed at similar levels in these conditions in *patl1* and *patl1 patl2* mutants versus wild type (Supplemental Figure 5G). The *BHLH039* promoter is directly responsive to low Fe and targeted by the upstream Fe deficiency signaling cascade (Zhang et al., 2015; Lei et al., 2020). *BHLH039* was similarly expressed between wild type and *patl2* and *patl1 patl2* mutants at 0 versus 50 *µ*M Fe (Figure 2I, Supplemental Figure 5H), while it was 25% less induced by 0 versus 50 *µ*M Fe in *patl1* (Supplemental Figure 5H). Ferritin gene *FER1*, a reliable marker indicating the Fe sufficiency status in our plant growth system (Naranjo Arcos et al., 2017), did not express differently in any condition (Figure 2J, Supplemental Figure S5, I and J).

Taken together, a most drastic phenotype of *patl2* mutants was observed for Fe reductase activity. PATL2 controls Fe reductase activity rather at protein level but not likely at gene expression level since *FRO2* expression remained unchanged between mutant and wild type.

Additionally, to evaluate the functional importance of the IRT1-PATL2 protein interaction, we employed the yeast *fet3 fet4* assay. The *fet3 fet4* yeast complementation assay was recently utilized to show that IRT1 interactor EHB1 had a negative effect on Fe transport via IRT1 (Khan et al., 2019). We found that the effects of PATL2, instead, were different. PATL2 had no growth-repressing effect in the IRT1-complemented *fet3 fet4* cells in the selective condition when growth relied on Fe^2+^ uptake by IRT1. A slight growth improvement was noted, which was not significant between PATL2-transformed and empty controls with the stringent statistical test applied, suggesting that the combined presence of IRT1 and PATL2 might have a growth-promoting and not a negative effect in *fet3 fet4* cells under Fe deficiency (Supplemental Figure 6).

In summary, among the tested Fe responses, PATL2 had a most drastic influence in negatively controlling Fe reductase activity in the root.

### The PATL2 interactome contained more proteins at 0 *µ* M Fe than 50 *µ* M Fe and was enriched in ROS and redox metabolism proteins

**Figure 3:**
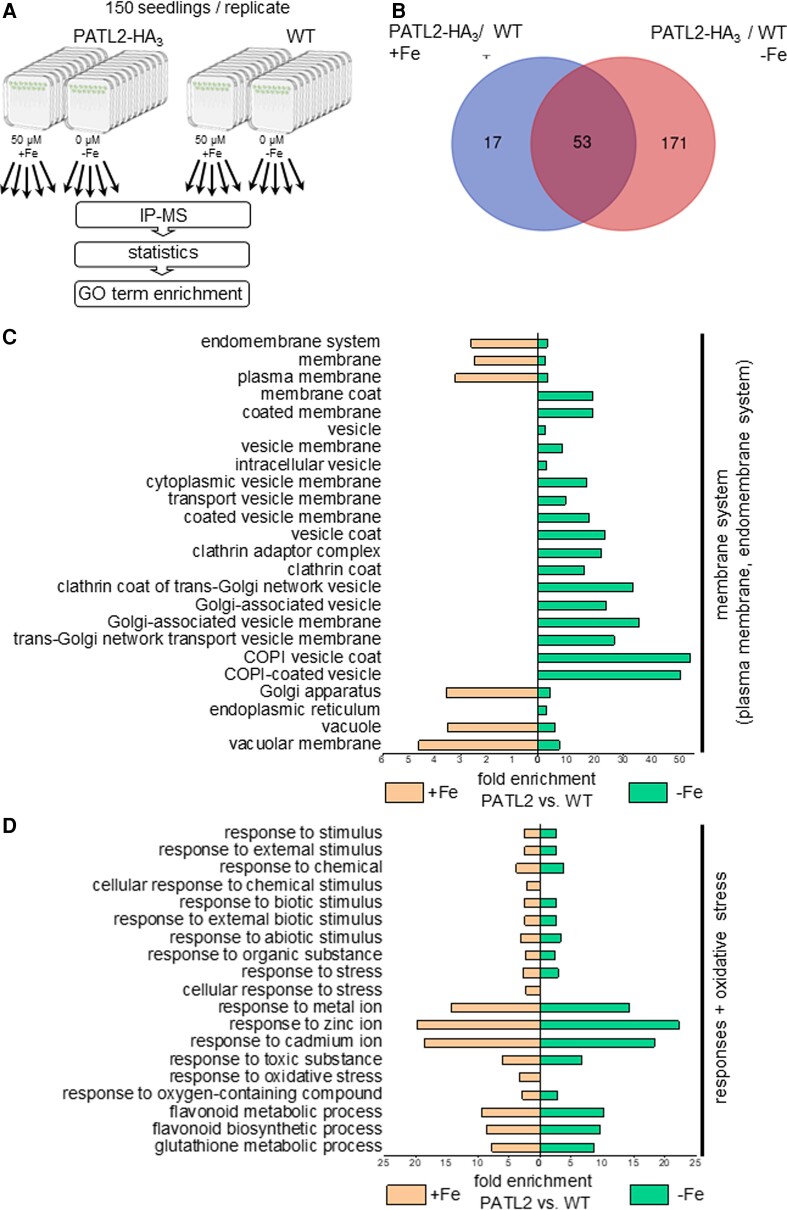
The PATELLIN2 (PATL2) interactome comprised more proteins at 0 *µ*M Fe than 50 *µ*M Fe and was enriched in membrane and oxidative stress-related protein functions. A, Overview of triple hemagglutinin (HA_3_)-tagged PATL2-HA_3_ interactome analysis. In total, 20 samples consisting of 5 biological replicates of PATL2-HA_3_ (pro35S::PATL2-HA_3_ plants) and wild type (WT) roots, each collected in the 14 + 3 d system under 50 *µ*M Fe (+Fe) and 0 *µ*M Fe (−Fe) were used for immunoprecipitation-mass spectrometry (IP-MS) analysis, followed by statistical analysis and GO term enrichment. The full workflow and additional information are detailed in Supplemental Figure 7. The construct pro35S::PATL2-HA_3_ complemented *patl2-2* ferric reductase phenotype (Supplemental Figure 7A). B, Venn diagram illustrating the number of identified proteins specific for the PATL2-HA_3_ interactome at + Fe and –Fe. Full protein lists are provided in Supplemental Table 1. C, D, Two selected functional categories identified after GO term enrichment under 0 and 50 *µ*M Fe in the PATL2-HA_3_ interactome versus WT. Additional information in Supplemental Tables 2 and 3.

Identifying proximity interactions between proteins and protein complexes is very critical for our understanding of the functional contexts of protein regulation. However, no information was available about the interworking of the molecular mechanisms and machineries of PATL2 in response to Fe supply in roots. Quantitative co-immunoprecipitation-coupled mass spectrometry (IP-MS) analysis uncovers stable protein complexes, which we expected to shed light on the role of PATL2 in roots. For reliable PATL2 protein interactome identification we used five biological replicates of roots of wild type and pro35S::PATL2-HA_3_-expressing Fe-deficient (0 *µ*M Fe) and Fe-sufficient (50 *µ*M Fe) plants (Supplemental Figure 7, A and B for complementation of the *patl2* Fe reductase phenotype by pro35S::PATL2-HA_3_). Following a statistical filtering and enrichment procedure comparing PATL2-HA_3_ to wild-type control root samples (Figure 3A, Supplemental Figure 7, C and D), we retrieved a total of 224 co-immunoprecipitated proteins specific to the PATL2 interactome (Supplemental Table 1, sum of columns AC/AE for 50 *µ*M Fe, AD/AF for 0 *µ*M Fe). Interestingly, 171 out of the 224 proteins were detected in plants grown under 0 *µ*M Fe, while only 17 of them were detected under 50 *µ*M Fe, indicating that PATL2-HA_3_ is involved in more functional protein complexes under 0 than 50 *µ*M Fe (Figure 3B, compare Supplemental Table 1, A and B and Supplemental Figure 7E). Among the enriched proteins and prospective PATL2-binding partners, we detected PATL1 and PATL4, indicating that the PATL2 protein complexes might contain multiple redundant PATL proteins. We did not identify IRT1 among the enriched proteins under Fe deficiency, presumably due to its overall low level of abundance as compared with that of the other proteins in the complex. The 224 proteins of the PATL2-HA_3_ interactome were enriched in diverse functions, irrespective of Fe nutrition (Figure 3, C and D; Supplemental Tables 2 and 3). The category of membrane systems was in line with the previously demonstrated membrane and cell plate localization of PATL2 (Peterman et al., 2004; Suzuki et al., 2016; Wu et al., 2017; Tejos et al., 2018; Montag et al., 2020). The direct association of PATL2 with the plant response and oxidative stress category was particularly exciting for us, as ROS and redox metabolism have emerged as a key system of balancing Fe acquisition in roots (Reyt et al., 2015; Le et al., 2016; Gratz et al., 2021; von der Mark et al., 2021). Several components of the PATL2-HA_3_ protein interactome belonged to the enzymatic antioxidant ROS-processing system, like ascorbate peroxidase (APX1), a catalase (CAT3), a glutathione peroxidase (GRX), and glutathione S-transferases (GSTs), that scavenge hydrogen or lipid peroxides, and redox-active thioredoxins (TRX) that regulate the cellular redox potential and transmit developmental and environmental signals (Supplemental Table 3, highlighted in yellow) (Noctor et al., 2018; Le et al., 2019). The recruitment of redox-active and ROS-scavenging enzymes by PATL2 is an indication that this SEC14 protein might attenuate cellular ROS stress or contribute to ROS signaling at the membrane in roots.

In summary, protein interactome studies of PATL2-HA_3_ showed that this SEC14 protein participates in protein complexes affected by Fe nutrition in roots, likely related to ROS- and redox-related antioxidant processes at the plasma membrane.

### Root lipid peroxidation, Fe reductase activity, and tocopherol contents in *patl2* and *vte2* mutants indicate a connection between Fe acquisition, oxidative stress, and vitamin E

**Figure 4:**
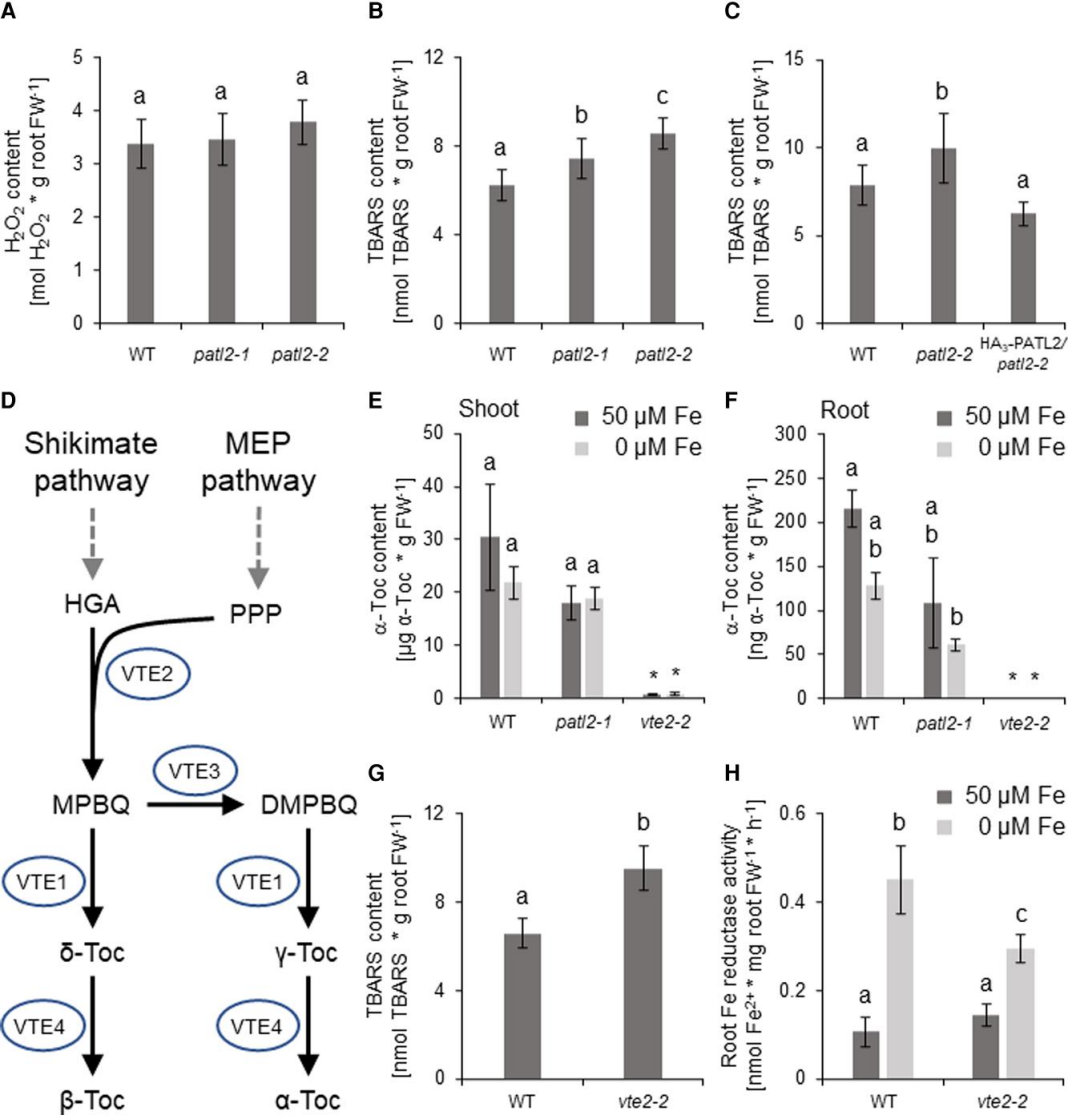
Root lipid peroxidation, Fe reductase activity, and tocopherol contents in *patellin2* (*patl2*) and *vitamin e2* (*vte2*) mutants indicate a connection between Fe acquisition, oxidative stress, and vitamin E. A, Root H_2_O_2_ concentration of wild type (WT), *patl2-1*, and *patl2-2* plants. B, C, Root TBARS content, indicating root lipid peroxidation levels in (B) wild type (WT), *patl2-1*, and *patl2-2* plants, and in (C) wild type (WT), *patl2-2*, and pro35S::PATL2-HA_3_ (PATL2-HA_3_)/*patl2-2* plants; triple hemagglutinin tag, HA_3_. Enhanced root TBARS contents and lipid peroxidation were found in *patl2* mutant plants versus WT. D, Schematic representation of tocopherol (Toc) biosynthesis and functions of tocopherol biosynthetic enzymes VTE1 to VTE4 with VTE2 catalyzing the key step. DMPBQ, 2,3-dimethyl-6-phytyl-1,4-benzoquinol; HGA, homogentisate; MEP, methyl erythritol phosphate; MPBQ, 2-methyl-6-phytyl-1,4-benzoquinol; PPP, phytyl pyrophosphate; VTE 1, tocopherol cyclase; VTE2, homogentisate phytyltransferase; VTE3, VITAMIN E DEFECTIVE 3, methyl transferase; VTE4, γ-tocopherol methyltransferase. E, F, α-tocopherol (α-Toc) contents in (E) shoots, (F) roots of WT, *patl2-1* and *vte2-2* plants. G, Root TBARS content of WT and *vte2-2* plants, 50 *µ*M Fe. H, Root Fe reductase activity of WT and *vte2-2* plants. *vte2-2* mutant plants had lower root Fe reductase activity and enhanced root lipid peroxidation levels. A–C, E–H, Plants were grown in the 14 + 3-d system. FW, fresh weight. A–C, E–H, data are represented as mean ± standard deviation. Different letters indicate statistically significant differences (*P* <0.05, determined by ANOVA with post hoc Fisher's LSD test). A–C, *n* = 5; (E–H) *n* = 3. Samples marked by * excluded from statistical analysis.

The complex with ROS-related enzymes provided a different prospective functional context for PATL2, relevant for Fe uptake. To explore it further at the physiological level, we determined levels of peroxides as they are substrates of many enzymes of the antioxidant system with which PATL2 is associated. Hydrogen peroxide (H_2_O_2_) is an oxidative stress signal molecule. We did not find any difference in its amounts between *patl2* and wild-type plants (Figure 4A). Due to the presence of PATL2 at the plasma membrane, we reasoned that lipid peroxides might be more important than H_2_O_2_. Thiobarbituric acid reactive substances (TBARS) are generated as side products in the lipid peroxidation-mediated membrane lipid breakdown. We used the TBARS content as a measure for lipid peroxidation and detected a 15% increase in *patl2-1* and a 30% increase in *patl2-2* mutant roots as compared with wild type (Figure 4B, complemented by PATL2-HA_3_ in Figure 4C). The sensitivity of *patl2* mutants to develop lipid peroxidation under Fe supply suggests that PATL2 has a protective role.

The observation that *patl2* mutants have a lipid peroxidation phenotype was very interesting because lipid peroxidation is reduced in the presence of antioxidants of the vitamin E group. In humans, hereditary genetic disease forms of ataxia with vitamin E deficiency are caused by defects of the SEC14 domain α-tocopherol transfer protein (α-TTP) (Atkinson et al., 2019; Arai and Kono, 2021). Therefore, the question arose whether vitamin E also plays a role in plant root lipid peroxidation and Fe acquisition and whether vitamin E is at all present in roots. *VITAMIN E* (*VTE*) genes 1–-4 encode the enzymes for biosynthesis of vitamin E compounds in plants (Figure 4D). *VTE* genes were expressed in roots in the 14 + 3 growth system, although to a lower level than in corresponding shoots. *VTE* genes were not differentially regulated by Fe nor by the absence or presence of PATL2 in roots (Supplemental Figure 8, A–D). Shoots had much higher tocopherol levels than roots. In both plant parts, α- and to lesser amount γ-tocopherol were the major tocopherols (Sattler et al., 2004; Mène-Saffrané, 2018) (Figure 4, E and F; Supplemental Figure 8, E and F). Interestingly, among different tocopherols, α-tocopherol levels were enhanced in roots grown in 50 *µ*M compared with 0 *µ*M Fe in wild type (Supplemental Figure 8, E and F). Neither the shoot nor root α-tocopherol contents were significantly differing between wild type and *patl2* mutant in the respective comparable conditions (Figure 4, E and F). There was only a significant difference between α-tocopherol contents in roots of 50 *µ*M Fe-treated wild type and 0 *µ*M Fe-treated *patl2-1* (Figure 4F). Hence, tocopherol contents were not found clearly affected by *patl2* mutation, however, tocopherol levels were regulated by Fe supply.

We tested whether a deficiency in vitamin E affects Fe acquisition responses. VTE2 is the key enzyme for vitamin E biosynthesis, and loss of function of *VTE2* leads to the elimination of a major α-tocopherol-generating pathway (Havaux et al., 2005) (Figure 4D). In consequence, the *vte2-2* mutant shoots had hardly any α-tocopherol (Figure 4E), as expected (Stahl et al., 2019), while roots of *vte2* mutants had no detectable level of α-tocopherol (Figure 4F). *vte2* mutant plants had a 50%-increased root TBARS content which was an increase in the same range as in *patl2* mutants under Fe supply, showing that tocopherols decrease lipid peroxidation in roots similar to PATL2 (Figure 4G). On the other hand, *vte2* plants had a 40%-decreased root Fe reductase activity at 0 *µ*M Fe supply (Figure 4H), thus the opposite phenotype as seen in *patl2* mutants. At the gene expression level, *FIT* was reduced by 30% in the *vte2* mutant under 0 *µ*M Fe compared to wild type (Supplemental Figure 9A). No significant differences were observed at the gene expression level for *BHLH039*, *FRO2*, *IRT1*, or *PATL2* nor of the Fe sufficiency marker *FER1* between *vte2* mutant and wild type (Supplemental Figure 9, B–F). Therefore, tocopherol plays a role to prevent lipid peroxidation and regulate Fe reduction. The discrepancy of Fe reductase activity between *patl2* and *vte2* mutants could be caused by more profound effects of vitamin E in cells (Sattler et al., 2004).

Taken together, PATL2 and the amount of antioxidant α-tocopherol in roots affected lipid peroxidation and Fe reduction. Furthermore, tocopherol contents were regulated by Fe supply. This indicates a connection between Fe acquisition, oxidative stress, and vitamin E.

### PATL2 binds the ligand α-tocopherol with high affinity in vitro

**Figure 5:**
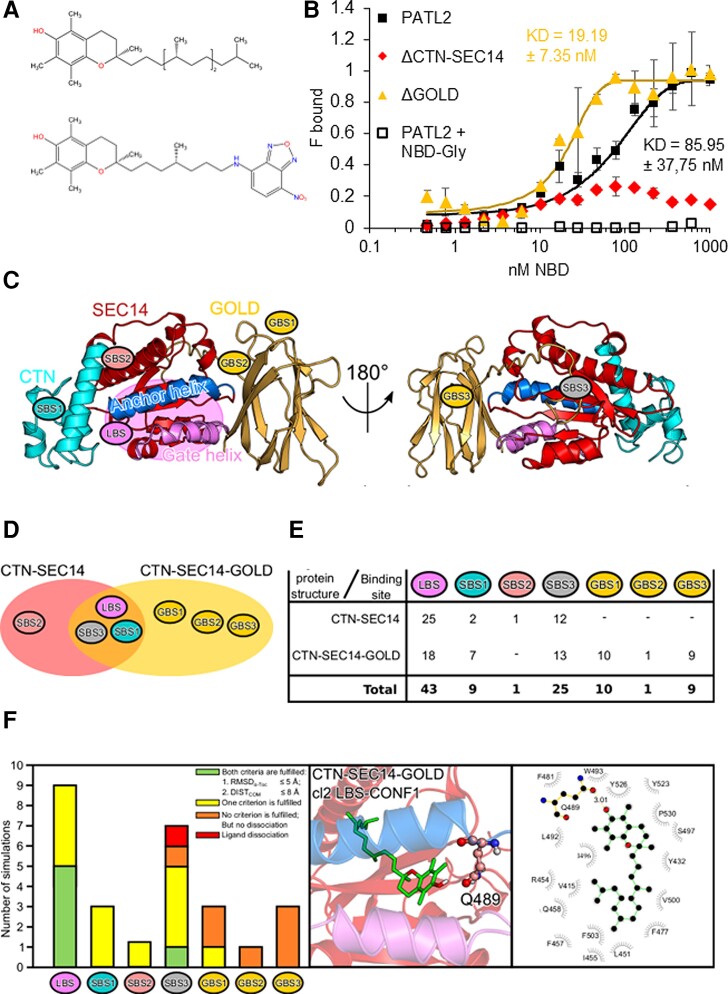
Biochemical in vitro and computational evidence for PATELLIN2 (PATL2) binding α-tocopherol inside the LBS of the SEC14 domain. A, Formula of α-tocopherol (upper structure) and nitrobenzoxadiazole (NBD)-α-tocopherol (lower structure). B, PATL2 protein-α-tocopherol ligand binding assay, using spectrofluorimetric measurements with Strep-tagged PATL2 (PATL2), PATL2 devoid of the Golgi dynamics domain (ΔGOLD), PATL2 devoid of the CRAL-TRIO-*N*-terminal extension, and SEC14 domain (ΔCTN-SEC14) with NBD-α-tocopherol (NBD-Toc). PATL2 with NBD-glycine (NBD-Gly) served as negative control. The assays were conducted using 50 nM protein and varying ligand concentrations as indicated. The fraction bound (*F*_bound_) corresponds to the relative fluorescence measured after 24 h of incubation (with maximum PATL2-α-Toc fluorescence set to 1). The dissociation constant (*K*_D_) was calculated for the PATL2 and ΔGOLD protein-ligand interaction with α-tocopherol at half *F*_bound_. Data are represented as mean ± Sd (*n* = 3), and best fitting of curves. Details on the establishment of the assay in Supplemental Figure 10. C–F, MD simulations of the PATL2-α-tocopherol. The full simulation workflow is shown in Supplemental Figure 11. C, Homology model of the CTN-SEC14-GOLD structure of PATL2 with the different docking sites of the CTN-SEC14 domain (SBS1-3), the LBS inside the CTN-SEC14 domain and the GOLD-binding sites (GBS1-3) in the front view (left side) and back view (rotated by 180°, right side). The different domains of PATL2 are labeled and colored; cyan, CTN; red, SEC14; orange, GOLD; blue, anchor helix; pink, gate helix; pink oval, LBS. D–F, Summary of the ensemble docking results for CTN-SEC14-GOLD and CTN-SEC14. D, Venn diagram showing the overlap of the binding sites. E, Number of docking events for the different binding sites. F, Left, histogram of the 27× 100 ns MD simulations of PATL2-α-Toc binding. α-Tocopherol-binding modes were validated by the average root mean square deviation of the ligand (RMSD_mean_) and the average distance between the centers of mass (COM) of the respective binding site and α-Toc (DIST_COM_) for stability during MD simulation for each protein conformation (homology model and three MD clusters, Supplemental Figures 16 and 17). Green, binding modes, fulfilling both criteria (RMSD_α-Toc_ ≤ 5 Å, DIST_COM_ ≤ 8 Å); yellow, fulfilling one criterion; orange, fulfilling no criterion; red, indicating dissociation of α-tocopherol. Middle panel, representative 3D configuration of stable α-tocopherol-binding to the LBS. The protein representation is the same as in (C), while α-tocopherol is shown as green sticks and the residue Q489 as ball-and-stick model. The side chain of Q489 forms a hydrogen bond with the hydroxyl group of α-tocopherol. Right, the corresponding α-tocopherol–protein interaction diagram (created with Ligplot+); gray half-circles, residues with hydrophobic interactions; orange, residues with salt bridges or hydrogen bonds; green, connection to the ligand. Detailed results of the analysis are provided in Supplemental Figures 11–20.

The interconnection of α-tocopherol and PATL2 in lipid peroxidation in roots and the link of these components to Fe mobilization made us predict that PATL2 binds α-tocopherol as a ligand, in analogy to α-TTP. Such a ligand interaction was also proposed for a *Lycopersicon esculentum* SEC14 protein and homolog of chloroplastic Arabidopsis PATL6 (Bermúdez et al., 2018). However, the affinity chromatography method that detected potential SlTBP-α-tocopherol-binding in a qualitative manner did not provide a proof in the form of physico-biochemical parameters of the ligand-binding (Bermúdez et al., 2018). Hence, a clear biochemical proof that a SEC14 protein from plants binds α-tocopherol was still lacking. Since α-tocopherol is barely soluble in an aqueous solution, we used a spectrofluorimetric method to demonstrate quantitative binding parameters of PATL2 and the ligand nitrobenzoxadiazole (NBD)-α-tocopherol (Nava et al., 2006) (Figure 5, A and B, Supplemental Figure 10). The NBD-ligand has an increased fluorescence upon binding inside a hydrophobic environment that is provided by the SEC14 lipid-binding site (LBS). Such fluorescence changes were not detected with the negative NBD-glycine control (Figure 5B). PATL2 as well as PATL2ΔGOLD bound NBD-α-tocopherol with high affinity in the nanomolar range in a similar order of magnitude as previously shown for human α-tocopherol-binding SEC14 proteins (Figure 5B, Supplemental Figure 10) (Panagabko et al., 2019). Interestingly, the PATL2ΔGOLD protein had a slightly higher affinity for the ligand than PATL2 (in average 19 nM versus average 86 nM, Figure 5B), suggesting that the GOLD domain might have a quenching effect. PATL2ΔCTN-SEC14, on the other hand, did not show binding with the ligand, indicating that a lipid-binding pocket and hydrophobic environment are only present in the SEC14 domain (Figure 5B, Supplemental Figure 10). These quantitative biochemical protein-ligand binding data show that binding of α-tocopherol to PATL2 occurs with a high affinity in vitro.

### Molecular simulations provide a structural model for the binding of α-tocopherol inside the SEC14 lipid-binding pocket of PATL2

Biochemical interactions are complemented in powerful manner and with high resolution by molecular dynamics (MD) simulations that illustrate binding characteristics and energetics at atomic and structural levels. We used MD simulations to investigate structural and energetic constraints of PATL2 required for α-tocopherol-binding. This theoretical approach used structural homology modeling, atomistic MD simulations, and free energy calculations (whole workflow in Supplemental Figure 11). In particular, we addressed whether the LBS pocket serves as a primary binding site for α-tocopherol, how the ligand is oriented at its binding site, and how stable its interaction with the protein is.

The first simulation step was to generate PATL2 structures by homology modeling since experimental ones were not available (Supplemental Figure 12). Because of the intrinsically disordered regions in the N-terminal part of PATL2 (Supplemental Figure 12A, left model), we focused on the C-terminal part of PATL2 devoid of the N-terminal region, either with GOLD domain, termed here PATL2-CTN-SEC14-GOLD, or without, termed here PATL2-CTN-SEC14 (Supplemental Figure 12A, middle and right models). The PATL2 homology models revealed that the cytosolic side of the SEC14 domain was predominately negatively charged, while the predicted membrane-oriented side was overall positively charged (Supplemental Figure 12, B–D). The LBS was flanked by two helical regions. Such regions direct SEC14 proteins into the membrane and control the opening and closing state of the LBS, known as the anchor and gate helices (Sha et al., 1998; Schaaf et al., 2008; Sugiura et al., 2019). Hence, these two helices of PATL2 were predicted to be oriented toward the membrane.

The resulting electrostatic potentials of the protein surfaces suggested that the gate helix interacts with the anionic lipids (such as PIPs) of the membrane via the positively charged surface. The GOLD domain had also positively charged areas (e.g. KKKK), which supports binding to negatively charged headgroups of the phospholipids in the plasma membrane. Furthermore, a polar linker connected the SEC14 with the GOLD domain. The SEC14 domains formed a LBS, which was the area where we suspected the ligand to bind. We simulated the PATL2-CTN-SEC14-GOLD and PATL2-CTN-SEC14 models for 500 ns to validate their stability and elucidate flexibilities (Supplemental Table 4). The root mean square deviation (RMSD) between the structures over time and the respective homology model indicated stability (RMSD <5 Å). The root mean square fluctuations (RMSFs) of the individual residues allowed us to identify flexible regions (Supplemental Figure 13). We found higher RMSD values compared to an earlier reported simulation of a SEC14 protein (Schaaf et al., 2011). Since this study reported a short simulation and RMSD values which were a magnitude lower than ours, the differences can be explained by the experimental setup of the simulations. We simulated the SEC14 protein in its lipid-free state, while Schaaf et al. (2011) simulated a lipid-bound state. We also included the GOLD domain in the simulation for PATL2-CTN-SEC14-GOLD, which was not the case in the other study. In the lipid-free state, the SEC14 protein region has more flexible areas, as revealed by the RMSF values of the individual residues (Supplemental Figure 13). During the simulation of the PATL2-CTN-SEC14-GOLD model, the GOLD domain rotated by about 90°, which was enabled by the high flexibility of the linker between the SEC14 and GOLD domains. The CTN and SEC14 domains, on the other hand, were very stable, apart from the N-terminal helix of the CTN domain and the already mentioned anchor helix region (RMSF values >4 Å). The findings were similar for the PATL2-CTN-SEC14 model. To identify the preferred protein structures in the MD simulations, we clustered them and analyzed the three most populated clusters that represented 78% of all PATL2-CTN-SEC14-GOLD conformations and 92% of all PATL2-CTN-SEC14 structures in more detail (Supplemental Figures 14 and 15). Interestingly, the LBS was not open in these three PATL2-CTN-SEC14-GOLD clusters, whereas it was open in the three PATL2-CTN-SEC14 clusters, indicating that the GOLD domain caused the LBS to close (Supplemental Figure 14). Hence, our simulations suggest a biochemical role for the GOLD domain, separated by a linker, in controlling the access to the SEC14 LBS.

In the second simulation step, we tested the binding of α-tocopherol to PATL2. To this end, we employed the ensemble docking approach where different protein structures were used in the docking process to include protein dynamics. We used four different protein conformations of both PATL2-CTN-SEC14-GOLD and PATL2-CTN-SEC14 (the homology model as well as the three main conformational clusters). We focused on three possible binding areas, the LBS, SEC14-binding sites (SBSs), and GOLD domain-binding sites (GBSs) and analyzed in detail for each of the three docking processes per protein form the five most favorable binding modes, as judged by their binding energies (Δ*G*, with more negative Δ*G* values representing better protein-ligand binding) (Supplemental Figure 16). The lowest Δ*G* values were obtained for four of the PATL2-CTN-SEC14 and seven of the PATL2-CTN-SEC14-GOLD binding modes, with the energies ranging between −3 and −9 kcal mol^−1^ (Figure 5C; Supplemental Figure 16, B and C). These binding modes predominantly involved the LBS, SBS1, and SBS3, when considering both protein forms (Figure 5D), whereby the LBS was by far the most important one (Figure 5E). The binding site SBS2 was of minor importance for either protein form. In PATL2-CTN-SEC14-GOLD, binding at GBS1 and GBS3 took place with a similar probability as at SBS1 (Figure 5C), while GBS2 turned out not to be ideal for binding of α-tocopherol (Figure 5, C–E; Supplemental Figure 17). In summary, the amphiphilic α-tocopherol molecule preferred to bind inside hydrophobic pockets that harbored polar and positively charged residues, which was best fulfilled by the LBS as well as SBS1, SBS3, GBS1, and GBS3.

To further evaluate the stability of the different α-tocopherol-binding modes, we performed 100 nsMD simulations of 17 binding modes for PATL2-CTN-SEC14-GOLD and 10 for PATL2-CTN-SEC14 (Supplemental Figures 17 and 18, Supplemental Table 4). The selection was made by using at least one conformation with lowest Δ*G* for each of the binding sites. Two criteria were used to assess the stability of the binding, (1) the average RMSD of α-tocopherol in its binding site <5 Å and (2) the average distance between the centers of mass of the binding site and α-tocopherol <8 Å. These two criteria, or at least one of them, were best fulfilled for LBS, second best for SBS3, and least for SBS2 and GBS2, which thus confirmed the docking results (Figure 5, E and F left side, Supplemental Figures 17 and 18). The MD simulations revealed six stable binding modes of α-tocopherol, five involving the LBS and one the SBS1 (Supplemental Table 5, Supplemental Figures 17 and 18, indicated by a *; Figure 5F, middle and right side, showing an example of stable binding of α-tocopherol to the LBS of PATL2-CTN-SEC14-GOLD, along with the interactions between the ligand and the amino acid residues; other examples in Supplemental Figure 19). The external α-tocopherol-binding might be an allosteric binding mode (Supplemental Figure 20) (Laskowski et al., 2009). Interestingly, the simulation revealed that the charged tocopherol head group could be oriented toward the entrance of the LBS, which might lead to a more open state of the gating helix if the internal ligand orientation occurs prior to membrane association.

Taken together, we conclude from the MD simulations that α-tocopherol can be most stably associated inside the LBS of the SEC14 domain. The high affinity of PATL2-α-tocopherol-binding determined in the biochemical ligand-binding assay therefore was most likely conferred by binding of α-tocopherol inside the LBS of the SEC14 domain. In this situation, the charged headgroup of α-tocopherol can be presented toward the opening of the binding pocket oriented toward the membrane.

## Discussion

**Figure 6:**
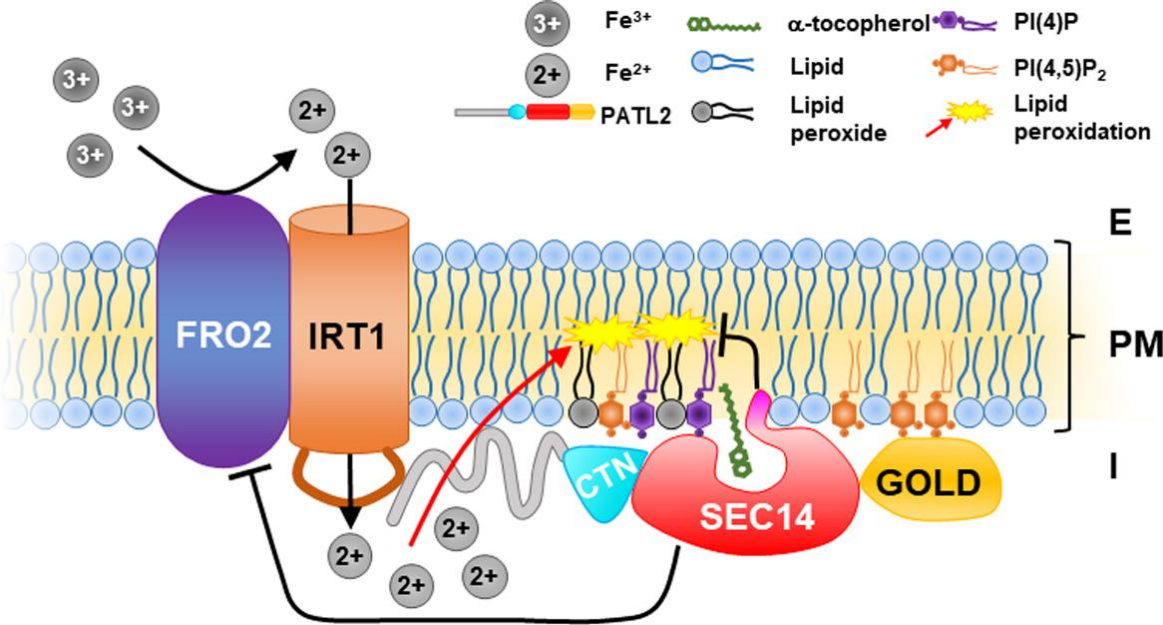
PATELLIN2 (PATL2) binds α-tocopherol and interacts with IRON-REGULATED TRANSPORTER1 (IRT1) to reduce membrane oxidative damage (summary working model). Import of ferrous iron (Fe2+, represented by circled 2+) via IRT1 bears the risk of oxidative damage and lipid peroxidation. FERRIC REDUCTASE OXIDASE1 (FRO2) and IRT1 are in close proximity at the plasma membrane (PM) (Martin-Barranco et al., 2020). External ferric Fe (Fe3+, represented by circled 3+; E, external) is reduced by FRO2 to generate ferrous Fe (Fe^2+^; Robinson et al., 1999). IRT1 imports Fe^2+^ (Vert et al., 2002). Internal Fe^2+^ (I, internal) reacts with reactive oxygen species (ROS) and polyunsaturated fatty acids which causes lipid peroxidation stress, represented in yellow (Le et al., 2019; Juan et al., 2021). PATL2 localizes with its CRAL-TRIO-N-terminal extension and SEC14 (CTN-SEC14) domain to PIP PI(4)*P* and PI(4,5)P_2_ and via the Golgi dynamics (GOLD) domain to PI(4,5)P_2_ contained in the PM, represented in violet and orange (Montag et al., 2020). In this work, it is shown that PATL2 interacts via its N-terminal region (represented by gray extension) with the variable region of IRT1 (IRT1vr, represented as brown loop of IRT1). α-Tocopherol, represented in olive green, is bound by the CTN-SEC14 domain of PATL2 in vitro and protects from lipid peroxidation stress. In this proposed model, the antioxidant reduces membrane oxidative damage during Fe import via IRT1. This highlights a novel mechanism of a SEC14 protein acting during cellular divalent metal ion import by way of the evolutionarily conserved ZINC AND IRON-REGULATED TRANSPORT PROTEIN (ZIP) family.

Identifying PATL2 as part of the IRT1vr protein interaction platform provided insight into the molecular operations of cellular metal ion uptake suggesting vitamin E-mediated protection from lipid peroxidation. First, IRT1 can bind PATL2, and PATL2 may come into proximity of IRT1 in the root epidermis of the root differentiation zone upon Fe deficiency, when both are expressed. Second, PATL2 affected root Fe reductase activity, and it is associated with a larger protein complex under Fe deficiency than Fe sufficiency showing that PATL2 is linked with Fe response regulation. Third, PATL2 was present in a complex with enzymes of the ROS response category and of the antioxidant system. α-tocopherol contents depended on Fe supply in roots, and they affected the ability of roots to reduce Fe. Finally, PATL2 was able to bind α-tocopherol in vitro, and the simulations provided a structural model how PATL2 may present α-tocopherol at the membrane. Taken together (Figure 6), we propose that IRT1vr may recruit PATL2. PATL2 may transfer α-tocopherol to IRT1 plasma membrane sites and with the help of a ROS response protein complex quench deleterious Fe^2+^ effects. Root Fe reductase activity may become compromised to reduce lipid peroxidation stress.

### Functional specificity of the PATL2 modular composition for IRT1 and α-tocopherol-binding sites

To date, still only little information had been available regarding the structural constraints of ligand binding and functional implications for SEC14 proteins with complex modular architecture in plants. PATL2 is recruited and attaches to IRT1 via its N-terminal region. It also attaches to the plasma membrane that is rich in PIPs which are recognized by binding-sites on the outside of the SEC14-GOLD domain. Furthermore, PATL2 binds a Fe-dependent protein complex which contains antioxidant enzymes. A fascinating hypothesis is that PATL2 is multifunctional and provides with its multi-domain architecture all features needed from transfer of α-tocopherol to the plasma membrane, IRT1 binding, and recruitment of the antioxidant system.

PATL2 can bind IRT1/IRT1vr, and this has been shown by applying three independent methods with tagged proteins (yeast two-hybrid assays, BiFC and Co-IP upon transient leaf epidermis transformation) and a deletion study by BiFC. Since the negative controls were as expected, as detailed in the “Result” section, it is unlikely, that the interaction data of the three assays were the result of interactions among the tags.

The deletion mapping helped to pinpoint the N-terminal part of PATL2 as the IRT1vr-interacting site. The homology model of PATL2 revealed that the large mostly intrinsically disordered N-terminal region, that interacted with IRT1vr, is set aside from the highly structured C-terminal CTN, SEC14, and GOLD domains. The N terminus, which is the most discriminating but also structurally flexible part within the PATL family (Peterman et al., 2004), was both necessary and sufficient for interaction with IRT1vr, and thereby specified biological functionality. The critical PATL2ΔN deletion mutant was previously found to bind lipids and localize to the plasma membrane similar to wild-type PATL2. Additionally, PATL2ΔN was stable in our computational molecular simulation (PATL2-CTN-SEC14-GOLD is equivalent to PATL2ΔN). Hence, the negative interaction of PATL2ΔN by BiFC suggests that it does not interact with IRT1vr, in agreement with the finding that the N-terminal part alone, namely as PATL2ΔC mutant, does interact. The binding is likely to occur in root epidermis cells as suggested by fluorescent protein localization studies. GFP-PATL2 and IRT1-mCitrine were present at the plasma membrane in the root differentiation zone and in root epidermis cells, when they were expressed from their endogenous promoters and grown side by side. The two proteins also co-localized at the plasma membrane upon transient expression. Thus, there is strong indication that PATL2 and IRT1 interact in root epidermis cells at the plasma membrane. Yet, it still remains to be demonstrated in future studies how localization of PATL2 relates to that of IRT1 when the two proteins are coexpressed in plant roots and IRT1 endocytosis is triggered. There are presently no functional data showing that proPATL2::GFP-PATL2 rescues the embryo lethality or the PATL2 phenotypes observed in this study. Yet, the available functional data with regard to localization at the root epidermis plasma membrane (Tejos et al., 2018 and this study) are in agreement with phospholipid binding in biochemical phospholipid and liposome-binding assays (Montag et al., 2020). The localization of proPATL2::GFP-PATL2 at the plasma membrane and at the cell plate (Tejos et al., 2018) is also in agreement with immunolocalization of PATL2 and PATL1 in plant cells (Peterman et al., 2004). PATL1, PATL2, and PATL4 might play largely redundant functions as they were all three detected in the root interactome, in agreement with their gene coexpression patterns in plants (Montag et al., 2020). Finally, we propose that the PATL unique N-terminal regions target plasma membrane proteins to diminish oxidative stress, like IRT1 and salt transporters (Chu et al., 2018; Zhou et al., 2018). The PATL2-associated categories endomembrane transport and ROS responses suggest that PATL2 is involved in remodeling the plasma membrane under stress and enabling ROS signaling. The phosphorylation sites in the N-terminal region of PATL2 (Lan et al., 2012) may be important for signal-driven post-translational modification and regulate the structure of the PATL2 protein to steer regulatory protein interactions of PATL2 in the response to environmental stresses.

The CTN-SEC14 domain, on the other side, is a canonical lipophilic-binding site, which contains the preferred and most stable α-tocopherol LBS. The PATL2ΔGOLD protein variant had similar to slightly higher affinity to α-tocopherol as PATL2, in agreement with the MD simulation data about PATL2-CTN-SEC14. The GOLD domain is clearly not necessary for the high-affinity binding to α-tocopherol. This is also in agreement with α-TTP, that does not have a GOLD domain, yet its dissociation constant for α-tocopherol is in a similar range as the one of PATL2 (Atkinson et al., 2019). The electrostatic potential surface of the SEC14 domain was mainly positively charged at the side of the gate and anchor helix, but negatively charged on the opposite side. Negatively charged PIs bind positively charged protein regions and thereby have a key role in regulation and binding proteins in the membranes, also in plants (Krauss and Haucke, 2007; Stahelin et al., 2014; Noack and Jaillais, 2020). Based on this, we can assume that the PATL2 gate and anchor helices are plasma membrane-oriented. The entrance between the LBS and the CTN domain had also a positively charged surface, so that lipids might enter or be released at this position. α-Tocopherol has amphiphilic properties. α-Tocopherol could adopt different stable binding modes within the LBS, whereby the headgroup of the ligand was oriented primarily toward the entrance of the binding site. This is very interesting, as PATL2 is thereby able to present the headgroup of α-tocopherol toward the membrane, so that the antioxidant role of this compound is exerted in the membrane. Only a single further stable binding site apart from the LBS was identified for α-tocopherol, which involved a region of the SEC14 domain. This latter binding mode may also have biological importance, e.g. cause an allosteric effect in substrate binding (Laskowski et al., 2009). On the other side, the additional binding site on the surface of the CTN-SEC14 domain may interact with charged headgroups of lipophilic substances, e.g. those present in the membranes. Surface binding sites adjacent to the LBSs and the gate helix could be involved in membrane attachment of PATL2, in agreement with PATL2-liposome-binding data (Montag et al., 2020) and as found for α-TTP (Zhang et al., 2011). The gate helix acts like a “lid,” and depending on its relative position to the LBS, the passage is more or less open or closed (Schaaf et al., 2008; Zhang et al., 2011). The GOLD domain also influenced the opening of the LBS of PATL2. Hence, the GOLD domain may control access of substrates to the LBS in certain circumstances, e.g. when being involved in certain protein complexes.

Future work may address binding of α-tocopherol to PATL2 in the presence of the lipid membrane or the insertion pathway of α-tocopherol into the LBS in the presence and absence of IRT1. The simulation results can be extended and exploited along with functional mutant plant complementation to predict and test specific amino acid residues of PATL2 that are crucial for the tocopherol-binding in the LBS. It will be interesting to identify a regulatory protein kinase that links the sensing of abiotic and nutrition stress with phosphorylation and activation of PATL2. It can be asked whether the phosphorylation cue of PATL2 is important for IRT1 binding, membrane association, lipophilic substrate ligand binding, and/or for recruitment of the ROS-related protein interactome. Finally, it will be interesting to identify which proteins bind the GOLD domain of PATL2 and may control lipophilic substrate availability or exchange in the membrane during Fe import and interaction with IRT1.

### Physiological and cellular integration of the IRT1-PATL2-α-tocopherol interaction

During Fe^2+^ import via IRT1, the Fenton-active divalent metal ions may cause membrane oxidative stress. The initiated radical cascade leading to lipid breakdown is halted by antioxidants. We detected high α-tocopherol levels in shoots, which is explained by the high-level biosynthesis in chloroplasts, reflected by the high expression level of *VTE* genes in agreement with available reports (Mène-Saffrané, 2018). *VTE* genes were also expressed in roots, but to a lower level, in agreement with low α-tocopherol levels in roots. Hence, we suspect that α-tocopherol was being synthesized in root cell plastids, as previously found for different plant species (Nowicka et al., 2021). Evidence is still disputed on the occurrence of tocochromanols in other compartments but plastids in plants (Nowicka et al., 2021). For example, ER-derived oleosomes are present in seeds, along with leucoplasts, and seeds are a source of plant oils and vitamin E. However, it is debated whether the vitamin E stems from oleosomes or leucoplasts. Some studies suggest that these antioxidant compounds can be present in other compartments. For example, the possibility of a transport of isoprenoid chromanol derivatives from chloroplasts to the ER was suggested due to a transorganellar complementation of the VTE pathway by ER-localized enzymes (Mehrshahi et al., 2013). We suspect that a transport from the plastids via the ER toward the membrane is possible in light of the recent discoveries on the close associations between these compartments (Brandizzi, 2021). Lipid transfer proteins such as the class of SEC14 proteins represent a way for the transport of lipophilic substances to target membranes besides vesicle transport even long-distance (Wong et al., 2017). In general, there is a connection of these antioxidant compounds with oxidative stress. It can therefore be assumed that isoprenoid chromanols including α-tocopherol play also fundamental roles in ROS signaling in the cell. Yet, the role of tocopherol in regulation and signaling outside of the chloroplasts is currently underexplored and more research is needed in this field to discover the relevant connections. Tocopherol radicals are reduced via the redox power of the ascorbate and glutathione cycles (Noctor et al., 2018; Le et al., 2019). Since such enzymes were part of the PATL2 interactome, this suggests that the reducing power is kept in vicinity of α-tocopherol and IRT1. This was also very interesting, because the underlying antioxidant enzyme genes, including *CAT3*, *APX1*, and several *GRX* and *GST* genes, are up-regulated in roots of Fe accumulation mutants, that constitutively up-regulated root Fe acquisition upon sufficient Fe (Le et al., 2019). Hence, the antioxidant system seems required during Fe uptake. ROS scavenging enzymes may thus have fast access to their poorly water-soluble substrates, and redox reconstitution of active α-tocopherol can occur in close proximity to IRT1 via the required redox-active enzyme machinery of the ascorbate-glutathione cycle. PATL2 may act at different levels to lower the risk of lipid breakdown, e.g. by presenting the α-tocopheroxyl radical headgroup at the membrane or by delivering α-tocopherol and releasing in exchange of phospholipids (Bankaitis et al., 2012; Kono et al., 2013; Huang et al., 2016; Wong et al., 2017; Panagabko et al., 2019; Sugiura et al., 2019). By similar mechanisms, PATL2 may also use its modular composition for changing the PIP landscape of the plasma membrane to prepare IRT1 endocytosis and/or degradation. PATL1 and PATL2 were reported to accumulate in plant extracellular vesicles, which carry signals and defense signals in the extracellular space (Rutter and Innes, 2017). This is interesting because these extracellular vesicles contained ROS-related functions, including APX1 and GST (GLUTATHIONE S-TRANSFERASE PHI2 [AT4G02520]) (Rutter and Innes, 2017), coinciding with the two PATL2 interactome-enriched GO categories ROS responses and endomembrane trafficking. In plants, ROS signaling via NADPH oxidase respiratory burst oxidase homolog (RBOH) proteins is connected with clathrin-mediated endocytosis (Lee et al., 2021), also dependent on extracellular Fe^2+^ (Martinière et al., 2019). The identified PATL2-interacting TRX3 possibly links redox functions to membrane-related processes. The *Brassica oleracea* TRX3 homolog, THL4, was localized to membrane compartments and participates in regulating plant receptor kinases (Cabrillac et al., 2001; Ivanov and Gaude, 2009). PATL2 may thus also act during endocytosis at the cross-roads of IRT1 plasma membrane protein regulation and ROS signaling. Plasma membrane-bound RBOH activation upon stress signaling involves activation by calcium-induced protein kinases of the CIPK family (Drerup et al., 2013; Han et al., 2019), that also control FRO2 activity, IRT1 protein stability, and activity of the transcription factor FIT, regulating transcriptional induction of *IRT1* and *FRO2* under low Fe (Tian et al., 2016; Dubeaux et al., 2018; Gratz et al., 2019a, 2019b). Hence, it is possible that PATL2 has a broader function in bridging ROS and Fe signaling at the membrane. Clearly, ROS signaling is tightly linked with Fe acquisition regulation (Le et al., 2016, 2019; Gratz et al., 2021; von der Mark et al., 2021), and the connection between IRT1 and PATL2 adds another layer of ROS regulation.

An interesting aspect is also that Fe reductase activity itself could be linked with vitamin E. The enzymatic antioxidant chain ultimately consumes NADPH, and that might compromise the NADPH oxidase activity of FRO2. NADPH oxidation is predicted to occur at the cytoplasmic side of FRO2 (Schagerlöf et al., 2006). Perhaps, PATL2 binds FRO2 and thereby affects FRO2 activity negatively or NADPH levels control ferric reduction. The opposite Fe reductase activity phenotype of the *vte2* mutant, on the other side, can be explained by a more profound effect on plant physiology. On the other hand, it is also possible that the increased Fe reduction of *patl2* roots is a sum of different Fe redox changes including enzymatic and non-enzymatic processes in roots. The lower Fe reductase activity of the *vte2* mutant speaks for the necessity of α-tocopherol for Fe acquisition. More research is needed to explore the cause of the elevated Fe reduction activities in *patl2* roots, and whether this involves FRO2 regulation.

### Conclusions and perspectives of the study

In conclusion, we propose that the interaction of IRT1vr with the α-tocopherol-binding SEC14-GOLD protein PATL2 is relevant for Fe homeostasis to control oxidative stress. The proposed model offers a basis for future research to understand the role of tocopherol and of SEC14 proteins during Fe homeostasis in roots. The proposed mechanism has not yet been demonstrated in roots during Fe acquisition. To overcome this limitation in the future, it will be important to test whether PATL2 co-localizes and interacts with IRT1 in root cells during Fe import, whether PATL2 enables lipophilic exchange between membranes upon its recruitment to IRT1 sites, and to answer whether the ROS scavenging enzyme machinery is recruited to catalyze α-tocopherol replenishment. It will be interesting to investigate the functional relevance of domains and specific amino acid residues of PATL2 for binding and presenting α-tocopherol to the membrane in roots to provide further support for the proposed mechanisms. Furthermore, it can be explored whether PATL2 has any effect on membrane remodeling and controlling the stability and localization of IRT1 and FRO2. Finally, we focus here on the connection with Fe homeostasis. It is possible that PATL2 acts in similar manner in other environmental stress situations leading to lipid peroxidation stress at the plasma membrane.

## Materials and methods

### Yeast two-hybrid (Y2H) assay

The screen of a cDNA expression library prepared from Fe-deficient roots against IRT1vr was previously described (Khan et al., 2019). The identified PATL2 coding sequence was retested in an independent targeted Y2H assay for validation of interaction, as described in Khan et al. (2019) using pairs of pGBKT-GW and pACT2-GW vectors with respective *IRT1vr* and *PATL2* fragments. Plasmids were co-introduced into *Saccharomyces cerevisiae* yeast strain AH109 and spotted on a double-selective SD medium lacking tryptophane and leucine (growth control), and SD medium lacking tryptophane, leucine, and histidine, and supplemented with 0.5 mM 3-AT, pH 5.8 (selective, growth indicates protein interaction). Yeast plates were pictured after 4 days of incubation at 30°C. Primers for molecular cloning are listed in Supplemental Table 6. Additional information is available in Supplemental Materials and Methods.

### Bimolecular fluorescence complementation

The 2in1 pBiFC-2in1 vector system (Grefen and Blatt, 2012) was used to verify protein–-protein interactions in transiently transformed *N. benthamiana* leaf epidermis cells. IRT1vr, PATL2, PATL2ΔN, PATL2ΔC, PATL2ΔCTN, PATL2ΔCTN-SEC14, PATL2ΔSEC14, and PATL2ΔGOLD fragments lacking stop codons were amplified and transferred to the pBiFC-2in1-CC vector. The generation of IRT1vr, PATL2ΔN, PATL2ΔC, PATL2ΔCTN, PATL2ΔCTN-SEC14, PATL2ΔSEC14, and PATL2ΔGOLD deletion fragments was described previously (Khan et al., 2019; Montag et al., 2020). Primers for molecular cloning are listed in Supplemental Table 6. Additional information is available in Supplemental File 1.

After *N. benthamiana* leaf infiltration using *Agrobacterium tumefaciens*, the RFP signal encoded by the vector was an indicator for a successful transformation event. The YFP signal indicated protein–protein interaction of proteins tagged to the split YFP forms (nYFP and cYFP). Further negative controls for BiFC using IRT1vr are found in Khan et al. (2019). Confocal images of fluorescent signals were collected as described below.

### Protein co-immunoprecipitation

PATL2 coding sequence was cloned into pAUL1 (Lyska et al., 2013) to express triple hemagglutinin-tagged PATL2-HA_3_ protein. *N. benthamiana* leaves were transiently transformed as described above to express combinations of IRT1-GFP and PATL2-HA_3_. Co-IP was performed as in Khan et al. (2019) using anti-GFP beads (ChromoTek). Input and elution fractions were separated by SDS-PAGE followed by immunoblot analysis to detect PATL2-HA_3_ and IRT1-GFP. Antibodies used were anti-IRT1 (AS11 1780; Agrisera, 1:5,000), goat anti-rabbit IgG horseradish peroxidase (AS09 602; Agrisera, 1:5,000), rat monoclonal anti-HA horseradish peroxidase conjugated (3F10 671; Roche, 1:5,000), and anti-HA high affinity (11867423001; Sigma-Aldrich, 1:1,000). IRT1-GFP had been localized at the plasma membrane and used for Co-IP studies to pull down IRT1-interacting HA_3_-EHB1, but not a non-interacting plasma membrane-associated mutant version of EHB1 (Khan et al., 2019). Primers for molecular cloning are listed in Supplemental Table 6. Additional information is available in Supplemental File 1.

### Plant material

Mutant Arabidopsis (*A. thaliana*) lines were *patl2-1* (SALK_086866), *patl2-2* (SALK_009882), *patl1-1* (Salk_080201), and *vte2-2* (Havaux et al., 2005), in Columbia-0 (Col-0, wild type) ecotype background. *patl1-1 patl2-2* double knock-out mutant were generated by crossing. *PATL1* and *PATL2* promoter::GUS transcriptional fusions were generated by amplifying 1,000 bp fragments from the respective upstream regions in the pMDC107 vector (Curtis and Grossniklaus, 2003) to yield pMDC107:PATL1 and pMDC107:PATL2. The constructs were used to generate transgenic plants by floral dip with *A. tumefaciens* C58C1 (pTiB6S3ΔT)^H^ strain. Homozygous T4 generations were used in this work. Primers for Gateway cloning of plasmids are listed in Supplemental Table 6. Additional information is available in Supplemental File 1. pAUL1:PATL2 (2xpro35S, pro35S::PATL2-HA_3_) was used to transform wild type and *patl2-2*, as indicated in the text, yielding PATL2-HA_3_ and PATL2-HA_3_/*patl2-2*. The lines pIRT1::GUS line (Vert et al., 2002), proIRT1::IRT1-mCitrine/*irt1* (Dubeaux et al., 2019), and proPATL2::GFP-PATL2 (Tejos et al., 2018) are described.

### Physiological studies

Sterilized Arabidopsis seeds were sown on agar plates and seedlings grown upright on Hoagland medium agar plates containing 50 *μ*M FeNaEDTA as previously described (Khan et al., 2019). In the “14 + 3 system,” 14-day-old plants were transferred to new Hoagland medium that was either Fe-sufficient containing 50 *μ*M FeNaEDTA (50 *μ*M Fe) or Fe-deficient with no FeNaEDTA (0 *μ*M Fe) for three days. In the “6- or 10-day system” seedlings were directly grown on 50 *μ*M Fe or 0 *μ*M Fe Hoagland plates for the indicated number of days. Plants were grown at 20°C in a 16 h-light/8 h-dark rhythm in climate chambers (CLF PlantClimatics). For seed harvesting, plants were grown in soil in the greenhouse. *N. benthamiana* plants were grown on soil for four weeks before transformation. Root length measurements were taken from seedlings grown in the 10-day system using ImageJ as previously described (Khan et al., 2019). SPAD values, a relative indication of chlorophyll contents, were determined in leaves of plants grown in the 14 + 3 system using SPAD 502 Plus Chlorophyll Meter (Spectrum Technologies, Inc.).

### Localization and co-localization of fluorescence protein reporter fusion proteins

For co-localization experiments, constructs expressing YFP-PATL2 (Montag et al., 2020), IRT1-mCherry (Ivanov et al., 2014) and Lti6b-mRFP (Caesar et al., 2011) were transiently expressed in *N. benthamiana* leaf epidermis cells. YFP-PATL2 was previously shown to localize to the plasma membrane (Montag et al., 2020) in agreement with immunolocalization of PATL1 and PATL2 in plant cells (Peterman et al., 2004). IRT1-mCherry was previously shown to co-localize with IRT1-GFP and early endosome proteins (Ivanov et al., 2014) and with EHB1-GFP at the plasma membrane (Khan et al., 2019). These previous localization patterns corresponded with the localization of GFP-PATL2 (Tejos et al., 2018) and with that of IRT1-mCitrine (Dubeaux et al., 2018).

For root cell localization of IRT1-mCitrine and GFP-PATL2, the lines proIRT1::IRT1-mCitrine/*irt1* (Dubeaux et al., 2019) and proPATL2::GFP-PATL2 (Tejos et al., 2018) were grown for 6 days directly on + and − Fe medium prior to investigation of the root hair zone using the LSM780 confocal setup, described above. Z-stacks were taken through the root to generate a dissection in *x–z* dimensions as indicated in the figure legends.

Confocal images of fluorescent signals were collected using the LSM780 system (Zeiss, Germany). YFP and mCitrine signals were excited at 514 nm, and emission was detected at 520–550 nm. mRFP and mCherry were excited at 561 nm, and emission was detected at 580–630 nm. GFP signals were excited at 488 nm, and emission was detected at 510–540 nm.

### Histochemical β-glucuronidase staining

Promoter-GUS reporter seedlings were incubated in GUS staining solution (50 mM sodium phosphate, 2 mM potassium ferrocyanide, 2 mM potassium ferricyanide, 0.2% (v/v) Triton X-100, and 2 mM GUS substrate 5-bromo-4-chloro-3-indolyl-b-D-glucuronic acid) (Jefferson et al., 1987). Stained seedlings were cleared in 70% ethanol. Pictures of seedlings were taken using a Zeiss Axio Imager M2 microscope at 20× magnification using the “Tiles” module and the “stitching” function of the software ZEN 2 (Zeiss) to assemble the collected images.

### Plant immunoblot

Protein was extracted from the ground plant tissue with 2× SDG (4% SDS w/v, 0.2M DTT, 20% glycerol v/v, 0.02% bromophenol blue, 1 *µ*L per mg tissue). After boiling for 10 min, 5 *µ*L was separated on a discontinuous 12% SDS-polyacrylamide gel and proteins transferred to a 0.2 *µ*m nitrocellulose membrane (Amersham Protran 0.2 NC nitrocellulose Western blotting membranes, Cytiva). Immunoblot analysis was performed with rabbit anti-IRT1 IgG (αIRT1, Agrisera AS11 1780), rabbit anti-FER IgG (αFER, Agrisera AS10 674), and rabbit anti-ACTIN (ACT) (Agrisera AS13 2640) each diluted 1:5,000 in 2.5% Milk-TBST buffer, secondary goat anti-rabbit IgG, and HRP conjugate (Agrisera AS09 602, diluted 1:10,000 in 2.5% Milk-TBST). Signals of enhanced chemiluminescence reaction (with Amersham ECL Select Western Blotting Detection Reagent, Cytiva) were detected by FluorChem Q (proteinsimple) and analyzed with AlphaView software (proteinsimple). Background-corrected signals were normalized to prominent bands of ACT immunostaining.

### Gene expression analysis by reverse transcription-quantitative PCR (Rt-qPCR)

RT-qPCR was performed according to Abdallah and Bauer (2016). Each biological replicate contained roots of 12 plants. Total RNA was isolated using the peqGOLD Plant RNA Kit (PeqLab). For cDNA preparation (RT reaction), Oligo dT primer and RevertAid first-strand synthesis kit (Thermo Scientific) were used. The DyNAmo ColorFlash SYBR Green qPCR Kit (Thermo Scientific) was used for qPCR in the CFX96 Real-Time System (BioRad). Melt curve analysis was performed using the CFX Manager software (BioRad). Absolute quantification was performed using mass standard curve analysis. qPCR primers for Fe response genes are listed in Naranjo Arcos et al. (2017) and Gratz et al. (2019a, 2019b), for *VTE* and *PATL* genes listed in Supplemental Table 6. Gene expression of samples was normalized to expression of reference gene *EF1Bα*.

### Root Fe reductase activity assay and determination of Fe content

Root Fe reductase activity was measured spectrophotometrically by the ferrozine method described in Le et al. (2016), using per sample a pool of five plants in 2 mL reaction solution with 500 *µ*M ferrozine, 100 *µ*M FeNaEDTA and applying for calculation an extinction coefficient for ferrozine-Fe^2+^ of 28.6 mM^−1^ cm^−1^.

Quantification of the Fe content of the dried seeds was measured as previously described (Khan et al., 2019). Samples were extracted with HNO_3_ in Multiwave 3000 (Anton Paar) and Fe was determined by inductively coupled plasma optical emission spectrometry (Ultima 2; HORIBA); λ_Fe_ = 259.940 nm. Additional information is available in Supplemental File 1.

### Yeast *fet3 fet4* complementation

The IRT1 yeast *fet3 fet4* complementation was conducted as in Khan et al. (2019). Yeast expression vector containing coding sequence of *PATL2* in pAG425GPD-ccdB-HA (Susan Lindquist, Addgene plasmid # 14250) or empty vector as control was transferred to yeast strains INVSc1, designated wild type, and DEY1453, designated *fet3 fet4*. Primers for molecular cloning are listed in Supplemental Table 6. Ten-fold dilutions of yeast cultures harboring combinations of IRT1 and PATL2 were plated on agar plates containing yeast extract peptone dextrose medium either supplemented with 50 mM of Fe^2+^ chelator bathophenanthrolinedisulfonic acid (BPDS, Fe-depleted condition) or without it (Fe-sufficient control condition). Yeast colonies were photographed, the density determined with ImageJ and quantified, as indicated in the figure legend.

### Co-immunoprecipitation-mass spectrometry (IP-MS) analysis of the PATL2 interactome

The workflow is detailed in Supplemental Figure 6A. Additional information is available in Supplemental File 1. Briefly, PATL2-HA_3_ and wild-type seedlings were grown in the 14 + 3 d system and exposed to 0 *µ*M Fe (−Fe) and 50 *µ*M Fe (+Fe) to harvest a total of 20 root samples, total of 5 biological replicates × 2 lines × 2 growth conditions each with 150 seedling roots each. The 20 samples were co-immunoprecipitated with anti-HA magnetic beads (Thermo Scientific, Pierce). The 20 IP samples (example in Supplemental Figure 6B) were processed as described in Grube et al. (2018) using an Ultimate 3000 rapid separation liquid chromatography system (RSLS, Thermo Fisher Scientific), detection by Orbitrap Fusion Lumos Tribrid mass spectrometer (Thermo Fisher Scientific). Fragment spectra were analyzed using the MaxQuant software (version 1.6.6.0, Max Planck Institute of Biochemistry, Martinsried, Germany). Searches were based on entries of the *A. thaliana* proteome set (UP000006548, downloaded from the UniProt Knowledgebase). Label-free quantification was enabled and proteins had to be detected by at least two different peptides and four valid values in at least one group. Perseus (version 1.6.6.0, Max Planck Institute of Biochemistry, Martinsried, Germany) was used for further processing. Samples were assessed by principal component analysis, leading to exclusion of one 50 *µ*M wild-type sample.

The PATL2 interactomes were identified by statistical enrichment of IP-MS data from the PATL2-HA_3_ samples compared to the wild type samples using two-tailed two-sample Student's *t* tests (S0 0.1, FDR 0.05) (Statistical analysis I). As an additional cutoff, proteins had to be co-immunoprecipitated and enriched in at least three PATL2-HA_3_ samples and in less than three wild type control samples (Statistical analysis II). The protein lists are summarized in Supplemental Table 1. Gene ontology (GO) analysis was performed using the Protein Analysis Through Evolutionary Relationships (PANTHER) system's GO tool, http://www.pantherdb.org (Mi et al., 2021). Protein descriptions were downloaded from The Arabidopsis Information Resource (www.arabidopsis.org). GO term enrichment was conducted and represented as described (Kar et al., 2021). The proteomics data are available via ProteomeXchange with identifier PXD032079.

### H_2_O_2_ measurement and lipid peroxidation assay

The H_2_O_2_ concentration was measured from 50 mg plant material using the Amplex Red Hydrogen Peroxide/Peroxidase Assay Kit (Thermo Fisher Scientific) and calculated following the protocol by Brumbarova et al. (2016). Lipid peroxidation was determined using the TBARS assay described in Zhang and Huang (2013) using 50 mg of plant material. The TBARS concentration was determined using the Lambert–Beer–Law and the extinction coefficient of malondialdehyde (MDA; 155 mM^−1^ cm^−1^). Additional information is available in Supplemental File 1.

### Analysis of tocopherol content by gas chromatography (GC)-MS

Following a described protocol (Stahl et al., 2019), 50 mg shoot and up to 100 mg root materials were ground, and tocopherols extracted using CHCl_3_:MeOH:H_2_O (1:2.5:1, v/v/v). The analytes were converted into trimethylsilyl derivatives by use of N-methyl-N-trimethylsilyltrifluoroacetamide containing 1% (v/v) trimethylchlorosilane in pyridine. Tocol was used as internal standard for quantification by GC-MS (GC 7890A equipped with a 5975C mass spectrometric detector; Agilent Technologies). For compound separation, a fused silica capillary column (Phenomenex ZB-35; 30 m × 30.25 mm × 30.25 mm) was used. Mass spectra were recorded in the range between mass-to-charge ratio (*m*/*z*) 50 and 750 in the electron ionization mode. The absolute content of tocopherols was calculated using previously established procedures and correction factors (Stahl et al., 2019). Additional information on the method is available in Supplemental File 1.

### Bacterial protein expression and purification of recombinant PATL2 protein

Recombinant Strep-tagged proteins (PATL2, PATL2ΔGOLD, PATL2ΔCTN-SEC14) were expressed in *Escherichia coli* Rosetta2 (DE3) pRARE2 cells using the recombinant pET52 vectors and protein expression procedures described in Montag et al. (2020). Protein was extracted, filtered, subjected to Strep-TactinXT 4Flow cartridge (iba)-based affinity chromatography, concentrated and further purified by Superdex 200 Increase 10/300 GL prepacked Tricorn Column (Cytiva)-based size-exclusion chromatography. Protein fractions were analyzed by SDS-PAGE and immunoblot (Supplemental Figure 10A). Purified protein was used for ligand-binding assays. Additional information is available in Supplemental File 1.

### NBD-α-tocopherol-PATL2 protein-ligand-binding spectrofluorimetric assay

NBD-α-tocopherol (NBD-Toc) was synthesized according to the protocol of Nava et al. (2006) and diluted. The protein-ligand-binding assay was established according to Jarmoskaite et al. (2020). Purified PATL2 protein was used in the amounts indicated in the text and figure legends (50–250 nM) and combined with 2.5 *µ*L of respective NBD-dilution to yield the respective final concentrations of NBD-Toc in 1% ethanol (v/v) in a total volume of 250 *µ*L (see Supplemental Figure 10, B–F). The protein-NBD-Toc mixture was incubated at 20°C for 20 h until reaching the equilibrium state (see Supplemental Figure 10, B–F). Fluorescence measurements were performed using the Infinite M200 pro plate reader (TECAN). The raw values were subtracted from respective background fluorescence (NBD-Toc in TKE, no protein). Finally, the fraction bound (*F*_bound_) was calculated using 50 nM protein and division of each replicate value by its highest value (upper equilibrium). The dissociation constant *K*_D_ and non-linear fit were calculated using the DoseResponse function in OriginPRO 2021. Additional information is available in Supplemental File 1.

### Homology modeling and MD simulation

The full workflow and methods for homology modeling and MD simulation of the PATL2 protein conformations (step 1) and MD simulation of the PATL2-α-tocopherol-binding (step 2) are summarized in Supplemental Figure 11 and Supplemental File 1. Briefly, homology models were obtained via I-TASSER (Roy et al., 2010; Zhang et al., 2011; Yang et al., 2015) using two protein forms, PATL2-CTN-SEC14-GOLD (residues 340-648) and PATL2-CTN-SEC14 (residues 340–565). The best model for each protein form (denoted as model 1) was used for MD simulation (500 ns) using GROMACS (Abraham et al., 2015; Lindahl et al., 2021) to identify three clusters that represented > 75% of the protein conformations for PATL2-CTN-SEC14-GOLD and PATL2-CTN-SEC14. α-Tocopherol conformations were determined using Avogadro (Avogadro, 2012; Hanwell et al., 2012) and Autodock (Goodsell et al., 1996; Santos-Martins et al., 2014). Ensemble docking using Autodock Vina (Trott and Olson, 2010) was used to determine PATL2-α-tocopherol-binding sites, which were assessed in MD simulations (100 ns) using GROMACS for both PATL2-CTN-SEC14-GOLD and PATL2-CTN-SEC14. For all MD simulations, the CHARMM36 force field and the corresponding TIP3P water model were used, the temperature was set at 293 K (20°C) and the pressure at 1 bar, and GROMACS v. 2020 (Abraham et al., 2015; Lindahl et al., 2021) was employed. If not stated otherwise, the simulations were analyzed using GROMACS tools. Figures of the protein structures were generated with PyMol (2015).

### Statistical analysis

Data were analyzed using one-way ANOVA followed by a post hoc test via Fisher’s least significant difference (LSD) using OriginPro 9.OG software, as indicated in the figure legends.

## Accession numbers

Sequence data from this article can be found in the GenBank/EMBL data libraries under accession numbers PATL2, At1g22530; PATL1, At1g72150; FIT, At2g28160; FRO2, At1g01580; IRT1, At4g19690; bHLH039, At3g56980; VTE1, AT4G32770; VTE2, AT2G18950; VTE3, AT3G63410; and VTE4, AT1G64970.

## Supplemental data

The following materials are available in the online version of this article.

**Supplemental Figure S1**. Localization of fluorescence protein-tagged PATL2 and IRT1 proteins suggest that PATL2 and IRT1 may interact at the plasma membrane in epidermal cells of the root differentiation zone.

**Supplemental Figure S2**. Localization of PATL2 and IRT1 promoter activity showed overlap in the root epidermis.

**Supplemental Figure S3**. Confirmation of patl1 and patl2 loss of function mutants.

**Supplemental Figure S4**. Localization of PATL1 and IRT1 promoter activity showed overlap in the root epidermis.

**Supplemental Figure S5**. Enhanced Fe reductase activity was the most drastic and consistent phenotype of patl1 patl2 but not patl1 loss of function mutants.

**Supplemental Figure S6**. Complementation of Fe-deficient yeast fet3 fet4 strain by IRT in the presence and absence of PATL2.

**Supplemental Figure S7**. Workflow and background of PATL2-HA3 interactome analysis.

**Supplemental Figure S8**. Gene expression of VTE genes was not regulated by Fe supply or dependent on PATL2 in roots, and α-tocopherol was the most abundant tocopherol in roots.

**Supplemental Figure S9**. Gene expression in tocopherol-deficient mutant plants.

**Supplemental Figure S10**. Establishment of NBD-α-tocopherol-binding assay.

**Supplemental Figure S11**. Workflow of the molecular simulation approach to test for the PATL2-α-tocopherol interaction.

**Supplemental Figure S12**. Additional information for molecular simulation: Homology modeling via I-TASSER and structural analysis of protein models.

**Supplemental Figure S13**. Additional information for molecular simulation: Analysis of 500-ns MD simulations.

**Supplemental Figure S14**. Additional information for molecular simulation: Clustering analysis and electrostatic potential surface (EPS) for the CTN-SEC14-GOLD protein model.

**Supplemental Figure S15**. Additional information for molecular simulation: Clustering analysis and electrostatic surface potential (ESP) for the CTN-SEC14 protein model

**Supplemental Figure S16**. Additional information for molecular simulation: Summary of docking results for α-tocopherol to PATL2-CTN-SEC14-GOLD and PATL2-CTN-SEC14.

**Supplemental Figure S17**. Additional information for molecular simulation: Molecular dynamics simulations of the CTN-SEC14-GOLD a-tocopherol (α-Toc) complexes obtained from ensemble docking.

**Supplemental Figure S18**. Additional information for molecular simulation: Molecular dynamics simulation of ensemble docking results of the CTN-SEC14 model and α-tocopherol (α-Toc).

**Supplemental Figure S19**. Additional information for molecular simulation: Best three binding modes of α-tocopherol (α-Toc) to binding sites of the CTN-SEC14-GOLD and CTN-SEC14 models.

**Supplemental Figure S20**. Additional information for molecular simulation: The potential “allosteric” binding mode of α-tocopherol

**Supplemental Table S1**. Summary of proteomics data, PATL2-HA3 interactome under sufficient and deficient Fe supply in roots.

**Supplemental Table S2**. PATL2-HA3 interactome—Summary of GO term enrichment.

**Supplemental Table S3**. PATL2-HA3 interactome—List of enriched proteins.

**Supplemental Table S4**. Summary of molecular simulations performed in this work.

**Supplemental Table S5**. Energies of the six best molecular dynamics (MD) binding poses.

**Supplemental Table S6**. Primer list.

**Supplemental Materials** and Methods.

## Supplementary Material

kiac563_Supplementary_Data
