# Supplementary material for: SEC14-GOLD protein PATELLIN2 binds IRON-REGULATED TRANSPORTER1 linking root iron uptake to vitamin E
Source: Plant Physiol. 2022 Dec 9;192(1):504–26. doi: 10.1093/plphys/kiac563 (PMC10152663; doi:10.1093/plphys/kiac563)
Supplement: kiac563_Supplementary_Data [file kiac563_supplementary_data.zip › PP2022RA00497R1_Supplemental_Table_S5.pdf]

Supplemental Table S5: Energies (ΔG_{bind}) and two criteria ($DIST_{COM}$, $RMSD_{\alpha\text{-}Toc}$) of the six best molecular dynamics (MD) binding poses.

System	ΔG_{bind} [kcal/mol]	$DIST_{COM}$ [Å]	$RMSD_{\alpha\text{-}Toc}$ [Å]
CTN-SEC14-GOLD-cl1-SEC14-CONF1	-8.6	3.07 ± 0.42	2.74 ± 1.08
CTN-SEC14-GOLD-cl2-LBS-CONF1	-3.2	1.56 ± 0.40	3.20±0.33
CTN-SEC14-GOLD-cl3-LBS-CONF1	-6.1	2.42 ± 0.49	4.09 ± 0.75
CTN-SEC14-m1-LBS-CONF1	-6.6	0.86 ± 0.29	3.48 ± 0.38
CTN-SEC14-cl1-LBS-CONF1	-8.9	1.66 ± 0.34	2.66 ± 0.21
CTN-SEC14-cl2-LBS-CONF1	-8.0	1.3 ± 0.40	4.34 ± 0.34

Mean value ± standard deviation (SD) are given for the two criteria ($DIST_{COM}$, $RMSD_{\alpha\text{-}Toc}$), whereby these values were calculated over 100 ns MD simulations for PATL2-α-tocopherol binding modes by using n= 10,000 datapoints. α-Tocopherol, α-Toc; CONF, configuration; CRAL-TRIO-N-terminal extension, CTN; cluster, cl; average distance between the centers of mass (COM), $DIST_{COM}$; Golgi dynamics, GOLD; lipid-binding site, LBS; root mean square deviation, RMSD; SEC14, SEC14.
