# Supplementary material for: SEC14-GOLD protein PATELLIN2 binds IRON-REGULATED TRANSPORTER1 linking root iron uptake to vitamin E
Source: Plant Physiol. 2022 Dec 9;192(1):504–26. doi: 10.1093/plphys/kiac563 (PMC10152663; doi:10.1093/plphys/kiac563)
Supplement: kiac563_Supplementary_Data [file kiac563_supplementary_data.zip › Supplemental Figures.pdf]

Supplemental Figures S1 to S20

SEC14-GOLD protein PATELLIN2 binds IRON-REGULATED TRANSPORTER1 linking root iron uptake to vitamin E

Jannik Hornbergs, Karolin Montag, Jennifer Loschwitz, Inga Mohr, Gereon Poschmann, Anika Schnake, Regina Gratz, Tzvetina Brumbarova, Monique Eutebach1, Kalina Angrand, Claudia Fink-Straube, Kai Stühler, Jürgen Zeier, Laura Hartmann, Birgit Strodel, Rumen Ivanov and Petra Bauer

Abbreviations used in the figures and figure legends

ANOVA, Analysis of variance
α–Toc, α-Tocopherol
CTN, CRAL-TRIO-N-terminal extension
GOLD, Golgi dynamics
IRT1, IRON-REGULATED TRANSPORTER1
LSD, Least Significant Difference
PATL, PATELLIN
SD, Standard deviation
SEC14, SEC14

Supplemental Figure S1

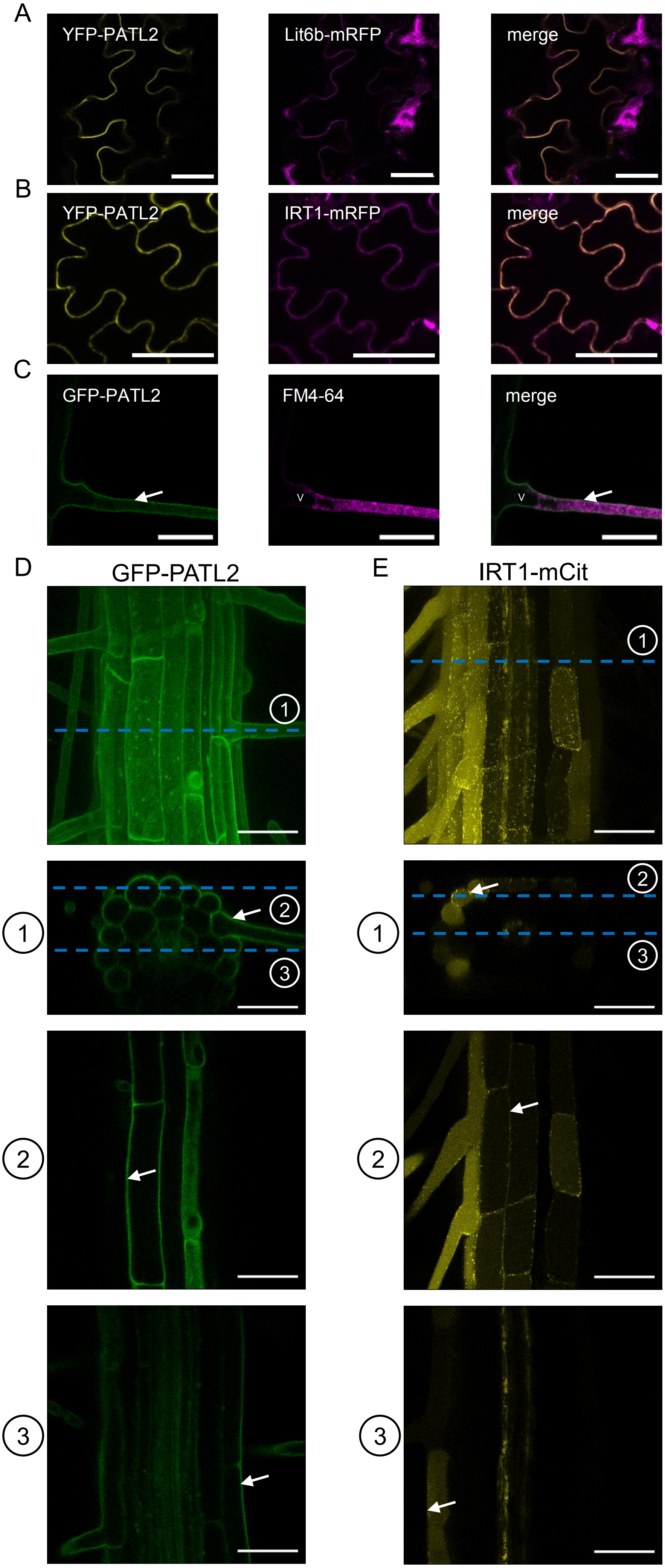

Supplemental Figure S1: Localization of fluorescence protein-tagged PATELLIN2 (PATL2) and IRON-REGULATED TRANSPORTER1 (IRT1) proteins

(A, B) Transient expression and co-localization of Yellow fluorescent protein (YFP)-PATL2 with (A) plasma membrane marker Lit6b-monomeric Red fluorescence protein (mRFP), and (B) IRT1-mRFP. Plant cells are *Nicotiana benthamiana leaf* epidermis cells. The YFP-PATL2, Lit6b-mRFP and IRT1-mRFP constructs have been used for localization in *N. benthamiana* leaves (Montag et al., 2020; Ivanov et al., 2014; Caesar et al., 2011). (C-E) Localization of fluorescent proteins in the root differentiation zones of transgenic Arabidopsis seedlings grown side-by-side in the 6-day system at 0 µM Fe. (C, D) Green fluorescent protein (GFP)-PATL2 in proPATL2::GFP-PATL2 seedlings previously described in Tejos et al. (2018). (C) Root hair cell stained with endocytosis marker FM-64. GFP-PATL2 signals are at the plasma membrane but not at the tonoplast (see arrow; v, vacuole). (E) IRT1-mCitrine in proIRT1::IRT1-mCitrine/ *irt1-1* seedlings previously described in Dubeaux et al. (2018). (D, E) Localization of fluorescent proteins in different optical planes. Maximum intensity projections (MIPs) were conducted in 2 µm slices. Plasma membrane localization is indicated by arrows. Size bar 50 µm. Top-most images, z-stack MIPs. The dashed blue lines indicate the planes of the cross sections shown below. Middle images, circled “1”, MIPs of cross sections. The dashed blue lines “2” and “3” indicate the planes of the longitudinal sections shown below. Lower images, longitudinal optical sections through the root epidermis, circled “2”, and through the root central stele, circled “3”. Size bar 50 µm.

Supplemental Figure S2

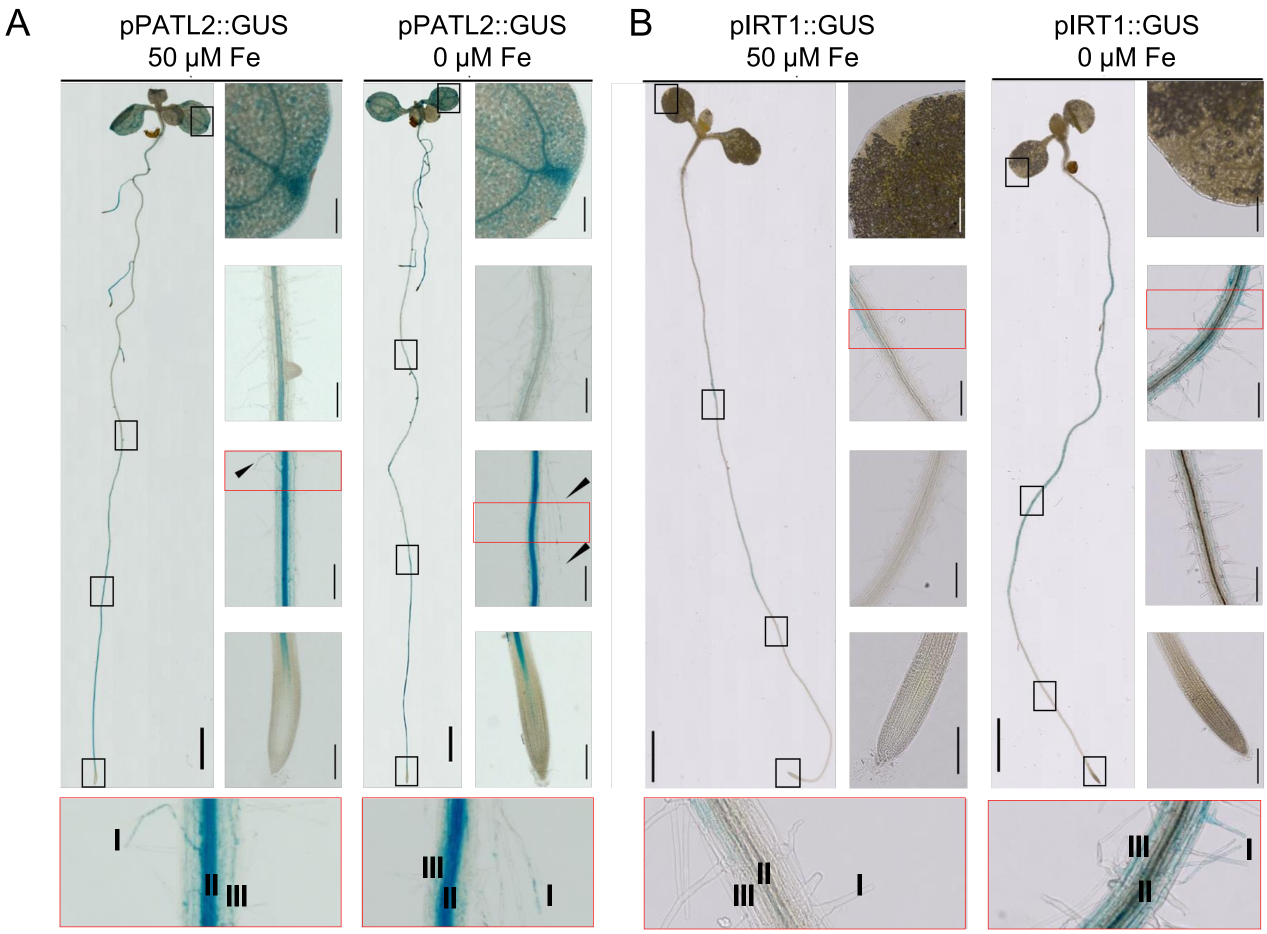

Supplemental Figure S2: Localization of *PATL2* and *IRT1* promoter activity showed overlap in the root epidermis

(A, B) Promotor-driven β-glucuronidase (GUS) activity in 8d-old Arabidopsis seedlings grown on either 50 µM Fe or 0 µM Fe; (A) *PATL2* promoter::GUS and (B) *IRT1* promoter::GUS. Left, whole plant; right, magnification of the regions marked in squares on the left, showing the cotyledon and different parts of the root zones. Arrowheads indicating GUS activity in root hairs. Bars 200 µm root magnification, 2 mm whole seedling images. Bottom, red boxes are magnifications of a 660 µm (width) x 220 µm (length) region within the root differentiation zone with (I) root hairs, (II) central cylinder and (III) root epidermis.

Supplemental Figure S3

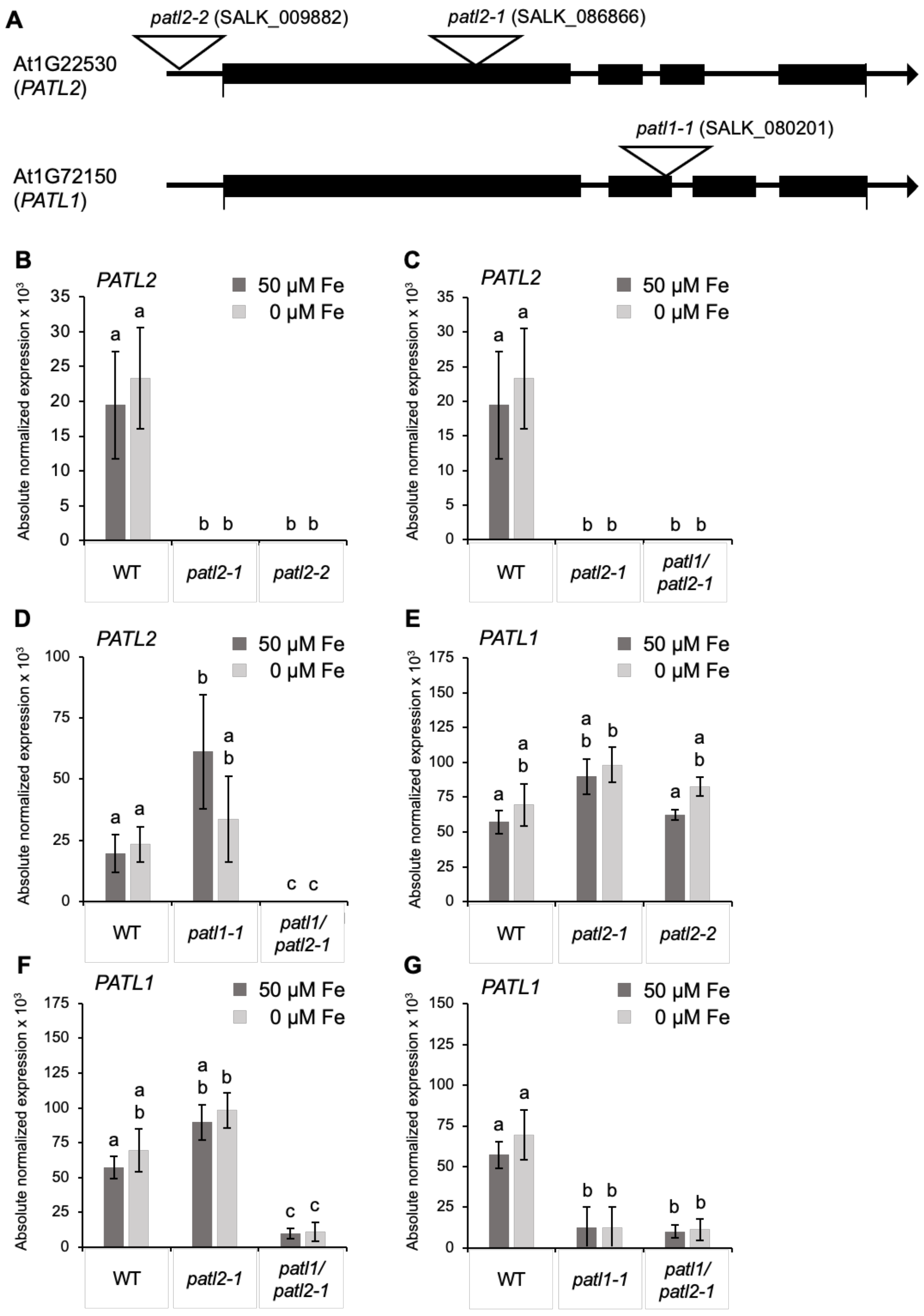

Supplemental Figure S3: Confirmation of *patl1* and *patl2* loss of function mutants

(A) Schematic representations of allele structures of *PATL2* (At1G22530) and *PATL1* (At1G72150) T-DNA insertion alleles (triangles, T-DNA insertions). Black boxes, introns. (B-G) *PATL2* and *PATL1* gene expression in roots of wild type, *patl2-1*, *patl2-2*, *patl1-1*, *patl1-1 patl2-2* mutant plants, as indicated. Plants were grown in the 14 +3 d system. Data are represented as mean ± standard deviation (SD). Different letters indicate statistically significant differences ($p < 0.05$, determined by analysis of variance (ANOVA) with post-hoc Fisher's Least Significant Difference (LSD) test) ($n = 3$).

Supplemental Figure S4

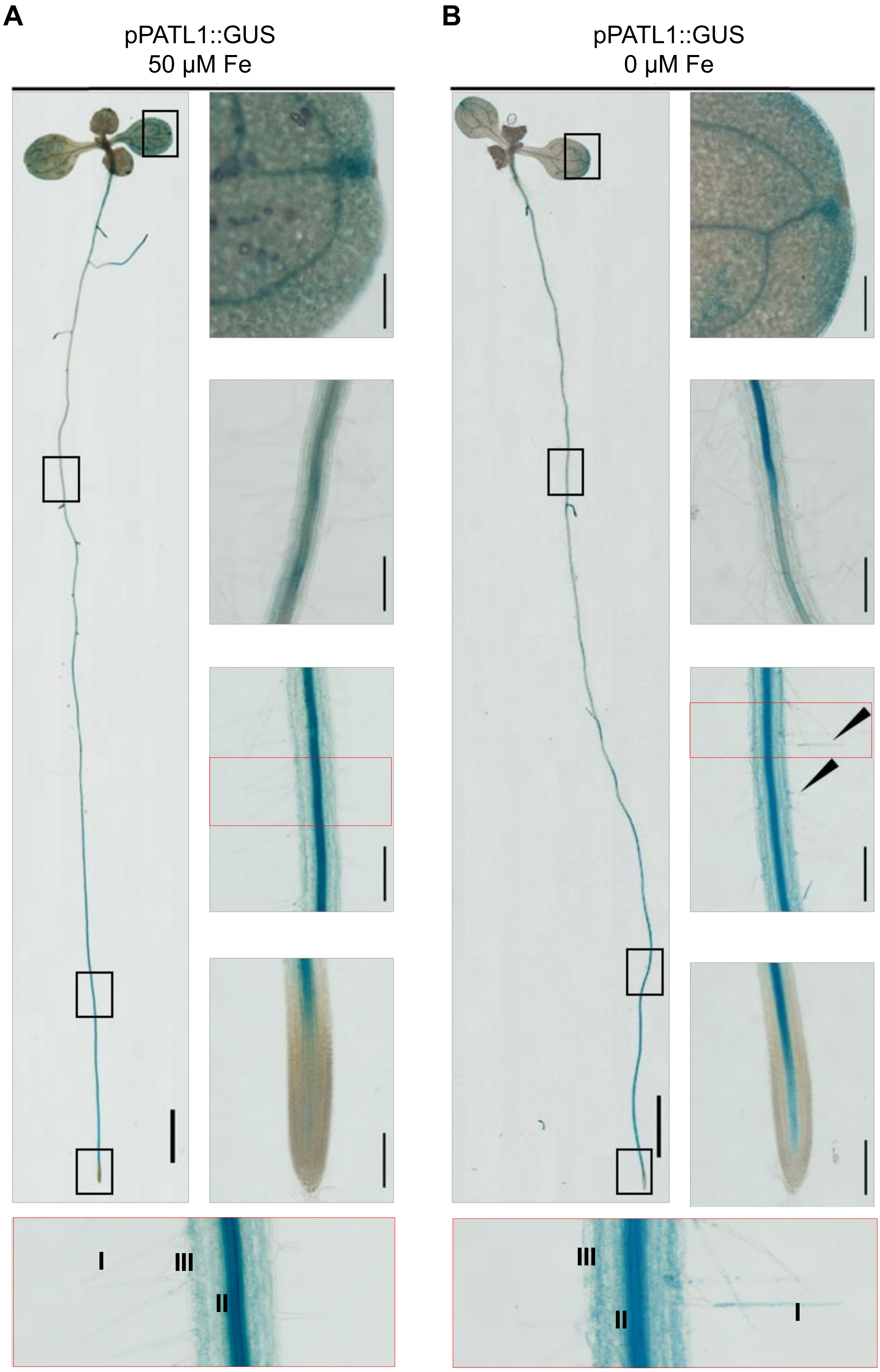

Supplemantal Figure S4: Localization of *PATL1* and *IRT1* promoter activity showed overlap in the root epidermis.

Promotor-driven β-glucuronidase (GUS) activity in 8d-old Arabidopsis seedlings grown on either 50 µM Fe or 0 µM Fe; (A) *PATL1* promoter::GUS and (B) *IRT1* promoter::GUS. Left, whole plant; right, magnification of the regions marked in squares on the left, showing the cotyledon and different parts of the root zones. Arrowheads indicating GUS activity in root hairs. Bars 200 µm root magnification, 2 mm whole seedling images. Bottom, red boxes are magnifications of a 660 µm (width) x 220 µm (length) region within the root differentiation zone with (I) root hairs, (II) central cylinder and (III) root epidermis.

Supplemental Figure S5

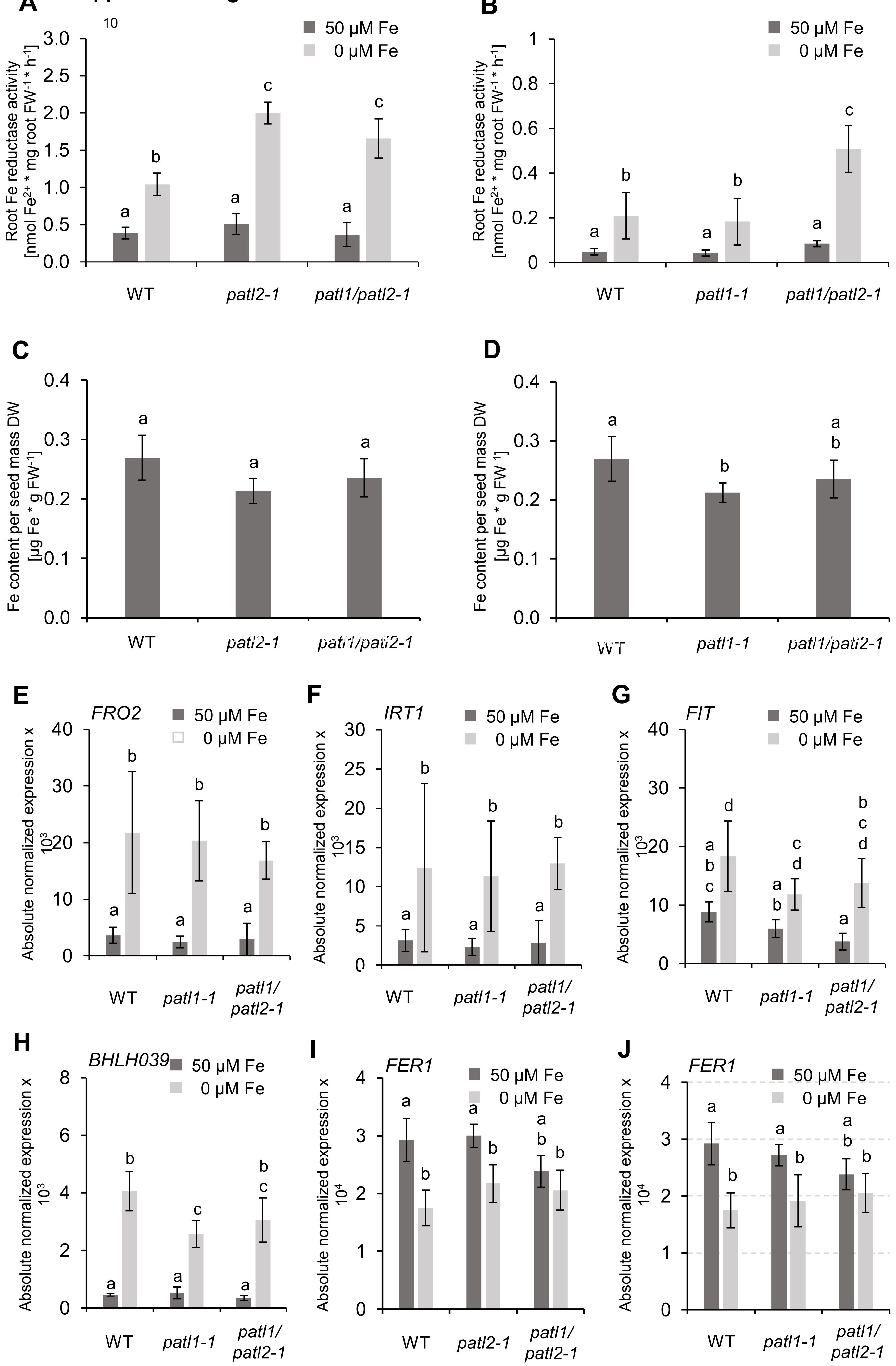

Supplemental Figure S5: Enhanced Fe reductase activity was the most drastic and consistent phenotype of *patl1 patl2* but not *patl1* loss of function mutants

Molecular-physiological analysis of Fe deficiency responses in roots of wild type (WT), *patl1-1* and *patl1-1 patl2-1* double mutant plants. (A, B) Root Fe reductase activity of double *patl1-1 patl2-1* mutants compared with (A) single *patl2-1* and (B) single *patl1-1*; fresh weight (FW) (C, D) Seed Fe contents per mass dry weight (DW). (E-J) Root Fe deficiency marker gene expression of (E) F*ERRIC REDUCTASE OXIDASE2* (*FRO2*), (F) *IRT1*, (G) FER-LIKE FE DEFICIENCY-INDUCED TRANSCRIPTION FACTOR (*FIT*), (H) *BASIC HELIX-LOOP-HELIX039* (*BHLH039*) and Fe sufficiency marker gene expression of (I, J) *FERRITIN1* (*FER1*), as indicated. (A-H) Plants were grown in the 14+3 d system. (A-J) Data are represented as mean ± SD. Different letters indicate statistically significant differences ($p < 0.05$, $n = 3$), determined by ANOVA with post-hoc Fisher's LSD test.

Supplemental Figure S6

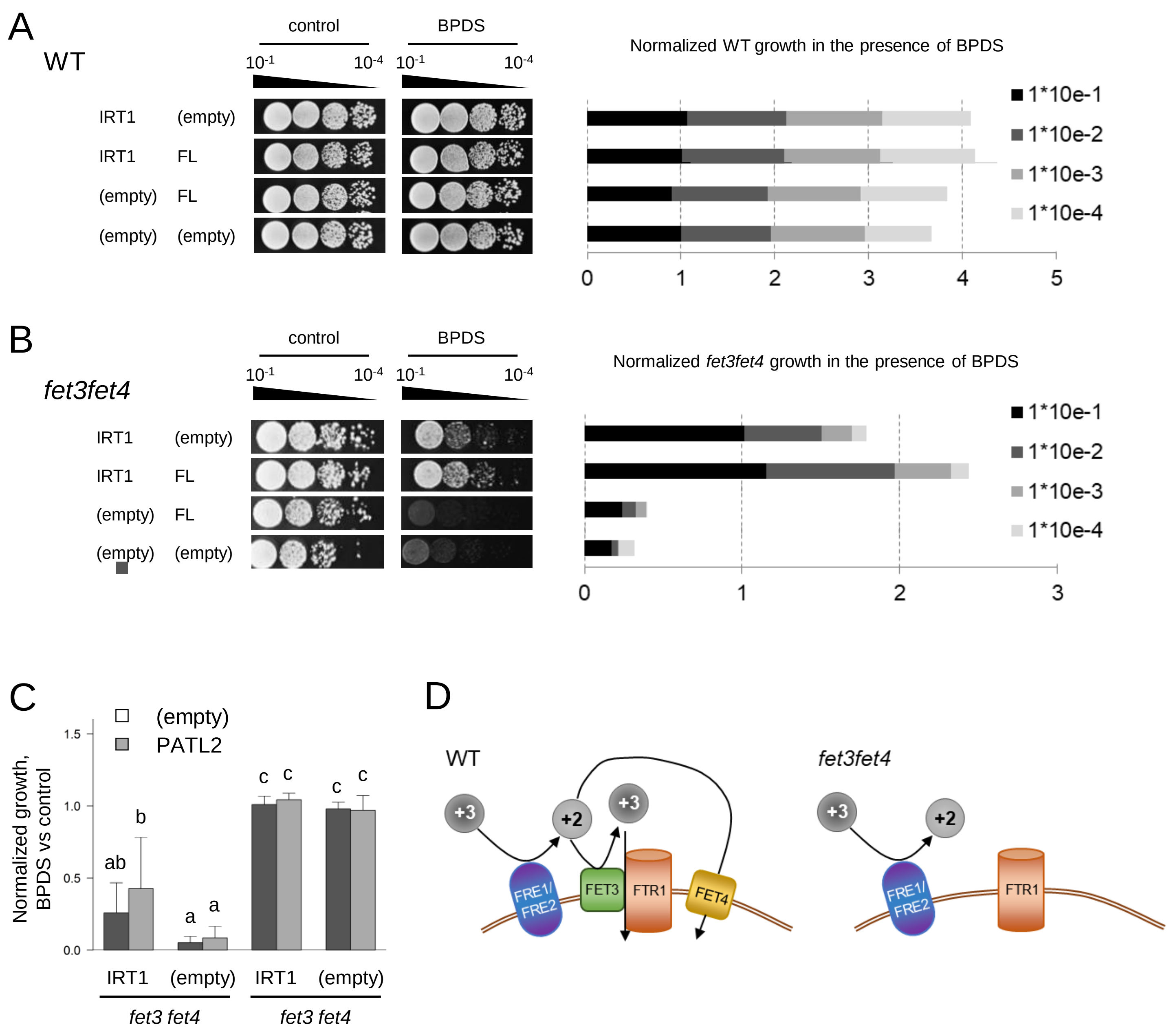

Supplemental Figure S6: Complementation of Fe transport (FET)-deficient yeast *fet3 fet4* strain by IRT in the presence and absence of PATL2. Yeast growth of wildtype and *fet3 fet4* strain on Fe-sufficient (control) and Fe-depleted (bathophenanthroline disulfonate, BPDS, Fe^{2+} chelator) medium transformed with IRT1 and PATL2 expression vectors or respective empty vector controls (empty), as indicated. (B), Quantification by colony density in BPDS medium normalized to respective control of (A). (C) Stem diagrams representing mean normalized values of (B) for the dilutions $1*10e^{-1}$ to $1*10e^{-3}$. Data are represented as mean ± SD. Different letters indicate statistically significant differences ($p < 0.05$, $n = 3$), determined by ANOVA with post-hoc Fisher's LSD test. (D) Scheme representing the *fet3 fet4* phenotype; FRE1, FRE2, ferric reductases; FET3/FTR1, multicopper oxidase/ Fe permease high-affinity Fe transport system; FET4, low-affinity Fe^{2+} transporter.

Supplemental Figure S7

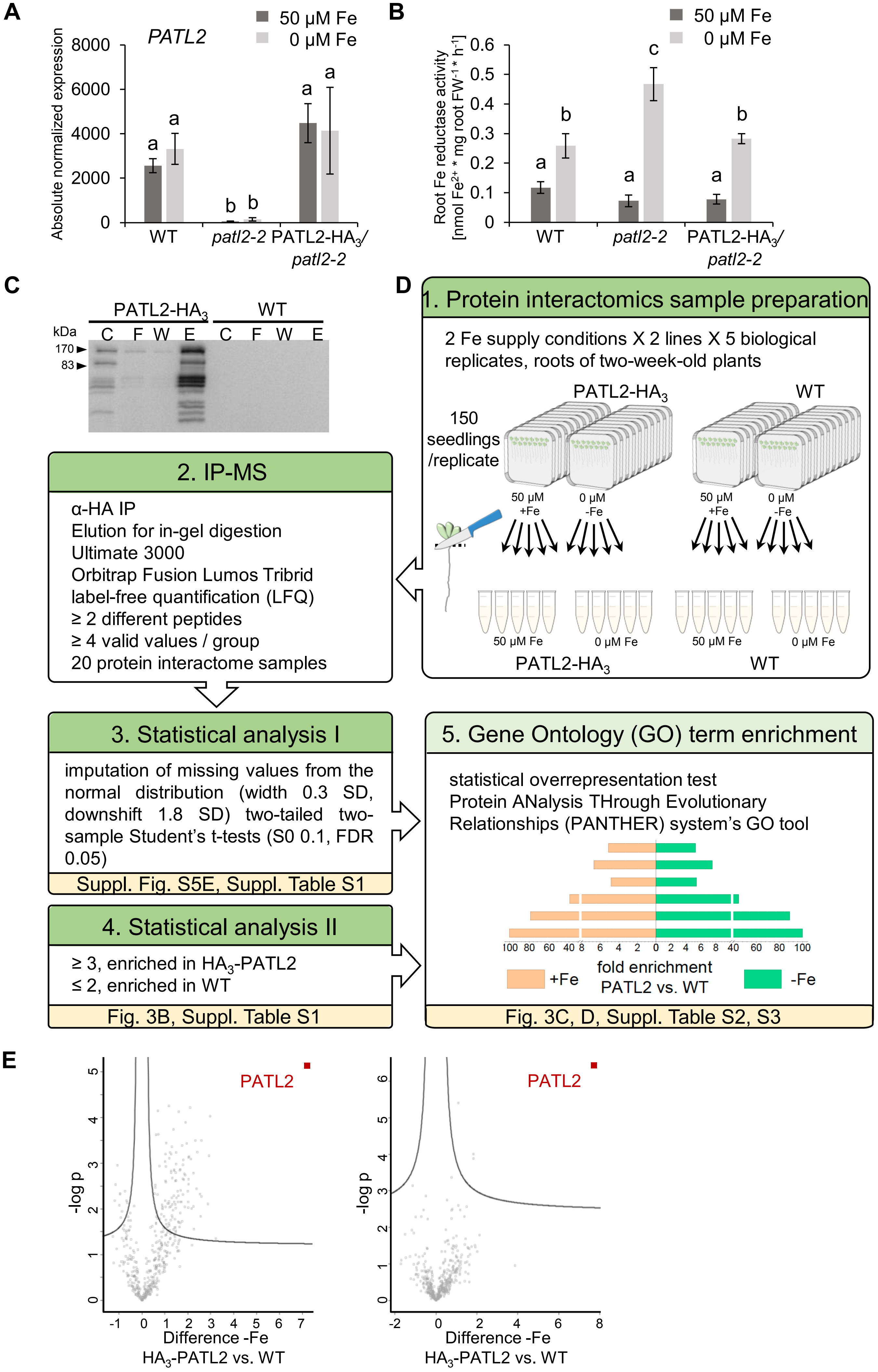

Supplemental Figure S7: Workflow and background of triple hemagglutinine-tagged (HA_3) PATL2-HA_3 interactome analysis. (A, B) Analysis of PATL2-HA_3 lines. (A) Gene expression analysis of *PATL2* in roots of wild type (WT), *patl2-2* mutant plants and pro35S::PATL2-HA_3/*patl2-2*. (B) Root Fe reductase activity of WT, *patl2-2* and pro35S::PATL2-HA_3/*patl2-2* roots showing complementation of Fe reductase activity by pro35S::PATL2-HA_3. FW, fresh weight. (A, B) Data are represented as mean ± SD. Different letters indicate statistically significant differences ($p < 0.05$, $n = 3$), determined by ANOVA with post-hoc Fisher's LSD test. (C-E) Overview of PATL2 interactomics by immunoprecipitation-mass spectrometry (IP-MS) and statistical data analysis. (C) Immunoblot of exemplary IP samples of pro35S::PATL2-HA_3 and WT roots. The expected PATL2-HA_3 protein bands are indicated by arrowheads. PATL2 protein forms a high molecular weight band (see Supplemental Figure S10A and Montag et al. 2020). Note the specificity, no protein is detected in the WT samples. Abbreviations used are C, crude protein extract (starting material); F, flowthrough; W, washing step 1 out of 5; E, elution. Equal volumes are loaded in each lane. (D) Flowchart of IP-MS analysis. In total, 20 samples consisting of five biological replicates of PATL2-HA_3 and wild type (WT) roots, each collected under 50 µM Fe (+Fe) and 0 µM Fe (-Fe) were used for IP-MS analysis, followed by statistical analysis (abbreviations SD, standard deviation; FDR, false discovery rate) and gene ontology (GO) term enrichment. (E) Volcano plots obtained by statistical analysis after data filtering (see (D) and Materials and Methods for further details). I. PATL2 protein was detected in the specific HA-IP fraction and is highlighted in red. (A-C) Plants were grown in the 14+3 d system under Fe-sufficient (50 µM Fe) and Fe-deficient (0 µM Fe) conditions.

Supplemental Figure S8

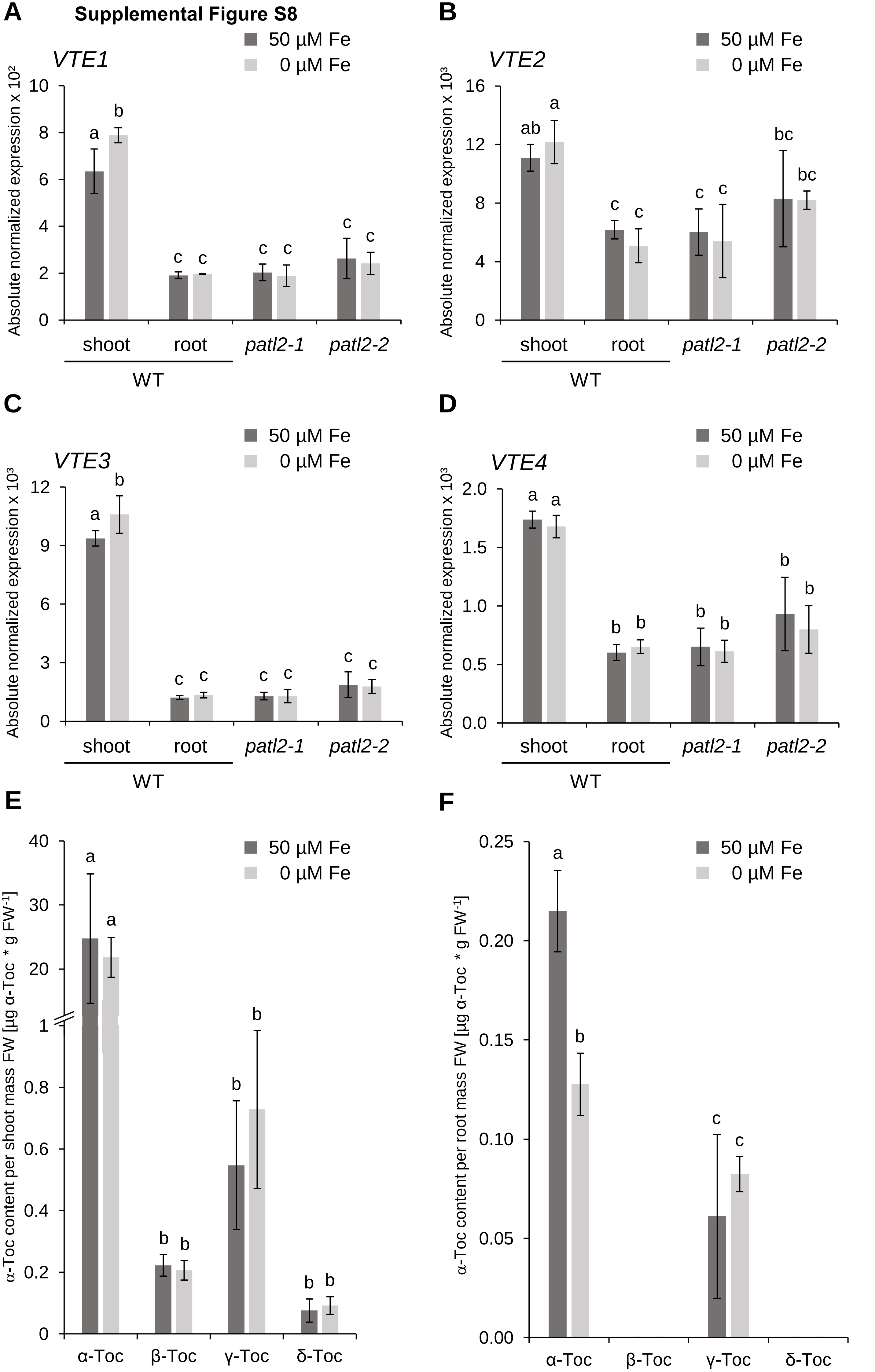

Supplemental Figure S8: Gene expression of *VITAMIN E* (*VTE*) genes was not regulated by Fe supply or dependent on PATL2 in roots, and α-tocopherol was the most abundant tocopherol in roots

(A-D) Gene expression analysis of tocopherol biosynthesis genes in shoots and roots of wild type (WT) and roots of *patl2* mutant plants, as indicated, (A) *VTE1* (B) *VTE2*, (C) *VTE3*, (D) *VTE4*. (E, F) α–, β–, γ–, δ-Tocopherol (Toc) contents per mass in (E) shoots, and (F) roots of WT plants. FW, fresh weight. Plants were grown in the 14+3 d system under Fe-sufficient (50 µM Fe) and Fe-deficient (0 µM Fe) conditions, shoots and roots harvested separately. (A-F) Data are represented as mean ± SD. Different letters indicate statistically significant differences ($p < 0.05$, $n = 3$), determined by ANOVA with post-hoc Fisher's LSD test.

Supplemental Figure S9

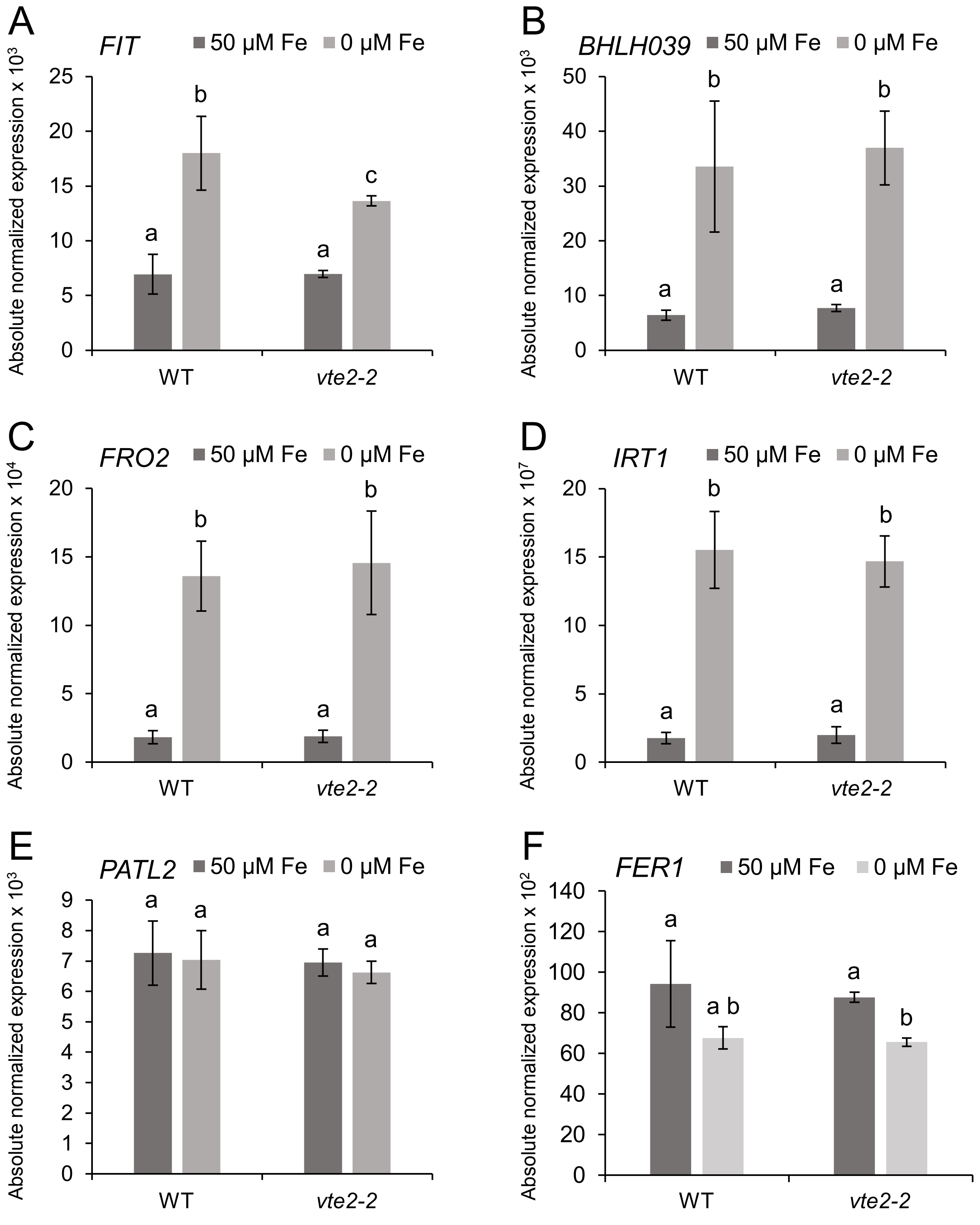

Supplemental Figure S9: Gene expression in tocopherol-deficient *vitamin e2* (*vte2*) mutant plants

Gene expression analysis of Fe deficiency marker genes in roots of wild type (WT) and *vte2-2* mutant plants, as indicated, (A) *FIT* (B) *BHLH039*, (C) *FRO2*, (D) *IRT1*, of (E) *PATL2*, and of Fe sufficiency marker gene (F) *FER1*. Plants were grown in the 14+3 d system under Fe-sufficient (50 µM Fe) and Fe-deficient (0 µM Fe) conditions. (A-F) Data are represented as mean ± SD. Different letters indicate statistically significant differences ($p < 0.05$, $n = 3$), determined by ANOVA with post-hoc Fisher's LSD test.

Supplemental Figure S10

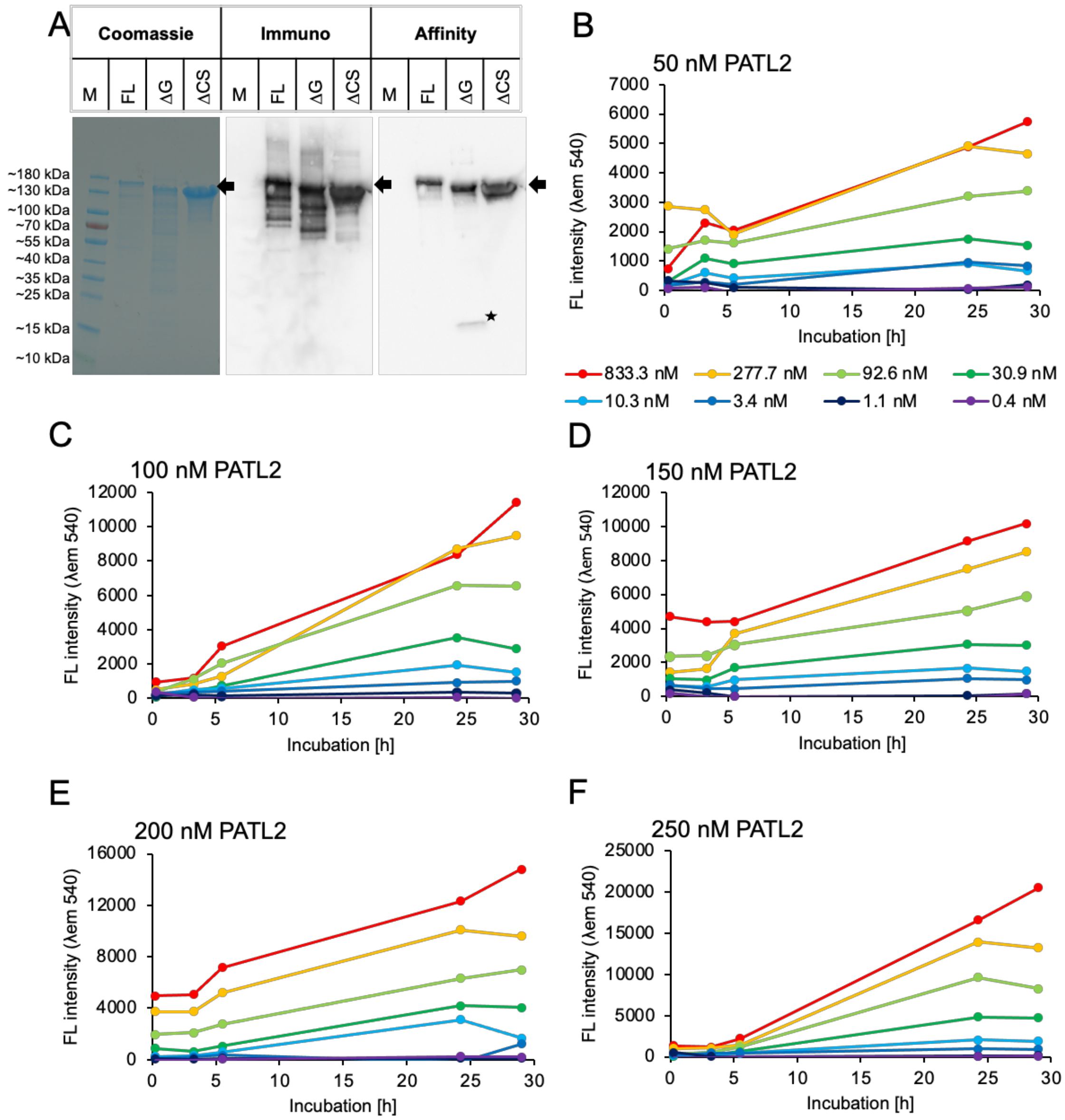

Supplemental Figure S10: Establishment of nitrobenzoxadiazole (NBD)-α-tocopherol binding assay.

(A) Quality control of PATL2 and PATL2 mutant protein forms used in protein-ligand binding assays. Strep-tagged protein was expressed in *Escherichia coli* and purified using affinity and size exclusion chromatography. Purified protein was checked by gel electrophoresis and specific protein detection. Left, Coomassie-stained protein gel (Coomassie); middle, immunoblot (Immuno) using α-PATL2-1 polyclonal antiserum; right, affinity blot (Affinity) using StrepTactin, showing high purity of PATL2 (full-length, FL), PATL2 deletion variants devoid of the Golgi dynamics (GOLD) domain (ΔG), the CRAL TRIO-N-terminal extension and SEC14 (CTN-SEC14) domain (ΔCS). Arrows indicate regions with expected protein bands; *highlights biotin carboxyl carrier protein, a co-purified contaminant from *E. coli*. Note that immunostaining identified bands below the expected appearance at approx. 170 kDa (FL), 160 kDa (ΔG) and 120 kDa (ΔCS) (see Montag et al., 2020; Peterman et al., 2004). (B-F) Establishment of the PATL2-NBD–α-tocopherol spectrofluorimetric protein-ligand binding assay. The assays were conducted from 0 up to 28 hours of incubation varying α-tocopherol ligand concentrations (molar, M; in y-axis FL, fluorescence light; emission, em) in the presence of different PATL2 protein concentrations, as indicated, (B) 50 nM, (C) 100 nM, (D) 150 nM, (E) 200 nM, (F) 250 nM PATL2 protein. The color code in (B) indicates the α-tocopherol ligand concentrations used in B-F. The 18-24 h time point and 50 nM PATL2 protein concentration were selected for determining the K_D according to Jamorskaite et al. (2020).

Supplemental Figure S11

1. Structural analysis of PATL2

Homology modeling
via I-TASSER

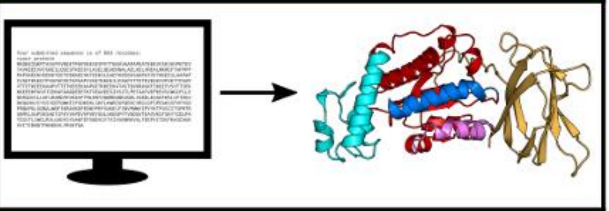

Methods: The webserver I-TASSER was used to obtain structures of PATL2, CTN-SEC14-GOLD and CTN-SEC14.

Result: Five model structures were obtained.

Conclusion: Models 1 had the best scores for the CTN-SEC14-GOLD and CTN-SEC14 systems to analyze their stability and flexibility.

Molecular dynamics (MD) simulations
via GROMACS (500 ns)

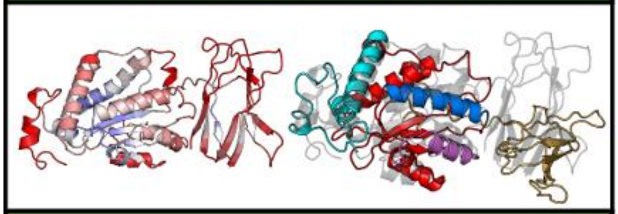

Methods: RMSD (domain level), RMSF (residue level), EPS, clustering (cutoff 3 Å)

Result: CTN-SEC14-GOLD and CTN-SEC14 proteins were stable; GOLD domain conferred flexibility.

Conclusion: Model 1 and the three first clusters (>75% structures) were applied for ensemble protein-ligand docking.

2. PATL2-α-Toc interactions

Ensemble docking
via Autodock via

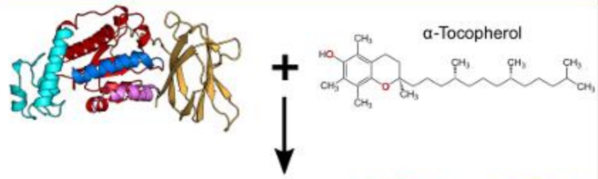

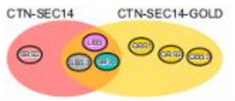

protein structure / Binding site	LBS	SBS1	SBS2	SBS3	GBS1	GBS2	GBS3
CTN-SEC14	25	2	1	12	-	-	-
CTN-SEC14-GOLD	18	7	-	13	10	1	9
**Total**	**43**	**9**	**1**	**25**	**10**	**1**	**9**

Methods: Three different grids (1) LBS, (2) SEC14 (SBS) and (3) GOLD domain (GBS) were used for the four protein structures; five α-Toc conformations (CONF1-5) were saved out from each docking.

Result: In total, seven binding sites (BS) were identified, α-Toc docked to the LBS with lowest binding energies.

Conclusion: For each protein structure and BS, at least one α-Toc conformation was used for MD simulations.

MD simulations
via GROMACS (100 ns)

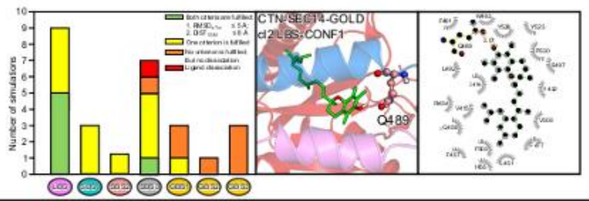

Methods: 1. Mean of RMSD of α-Toc ($RMSD_{α\text{-}Toc}$ ≤ 5 Å), 2. Mean of center of mass distance between BS and α-Toc ($DIST_{COM}$ ≤ 8 Å)

Result: α-Toc was stable inside the LBS, more flexible in other BS.

Conclusion: α-Toc bound stably to the LBS.

Supplemental Figure S11: Workflow of the molecular dynamics (MD) simulation approach to test for the PATL2-α-tocopherol interaction

The computational protein-ligand binding approach consisted of two major steps. I, Obtaining structural models of the PATL2-derived protein forms. Due to the intrinsically disordered nature of the N-terminal PATL2 region, the molecular simulations focussed on the CTN-SEC14-GOLD and CTN-SEC14 parts of PATL2. II, Molecular docking of α-tocopherol to the CTN-SEC14-GOLD and CTN-SEC14 protein forms to identify the binding sites and to verify these via subsequent MD simulations.

The inserted figures only serve illustration purposes for outlining the various steps in the simulation procedures in this workflow overview. Details on these inserted figures follow in the subsequent Figures and Supplemental Figures. Binding site, S Ḁ̀ configuration, CON Ḁ̀ average distance between he` centers o` mass (COM), $DIST_{COM}$` electrostatic potential surface, EPS` GOLD S,` GS Ḁ̀ lipid S,` LS Ḁ̀ roo` mean square deviation, RSD Ḁ̀ roo` mean square fluctuation, RMSF` SEC14 S,` SBS` α-Tocopherol, α-Toc.

Supplemental Figure S12

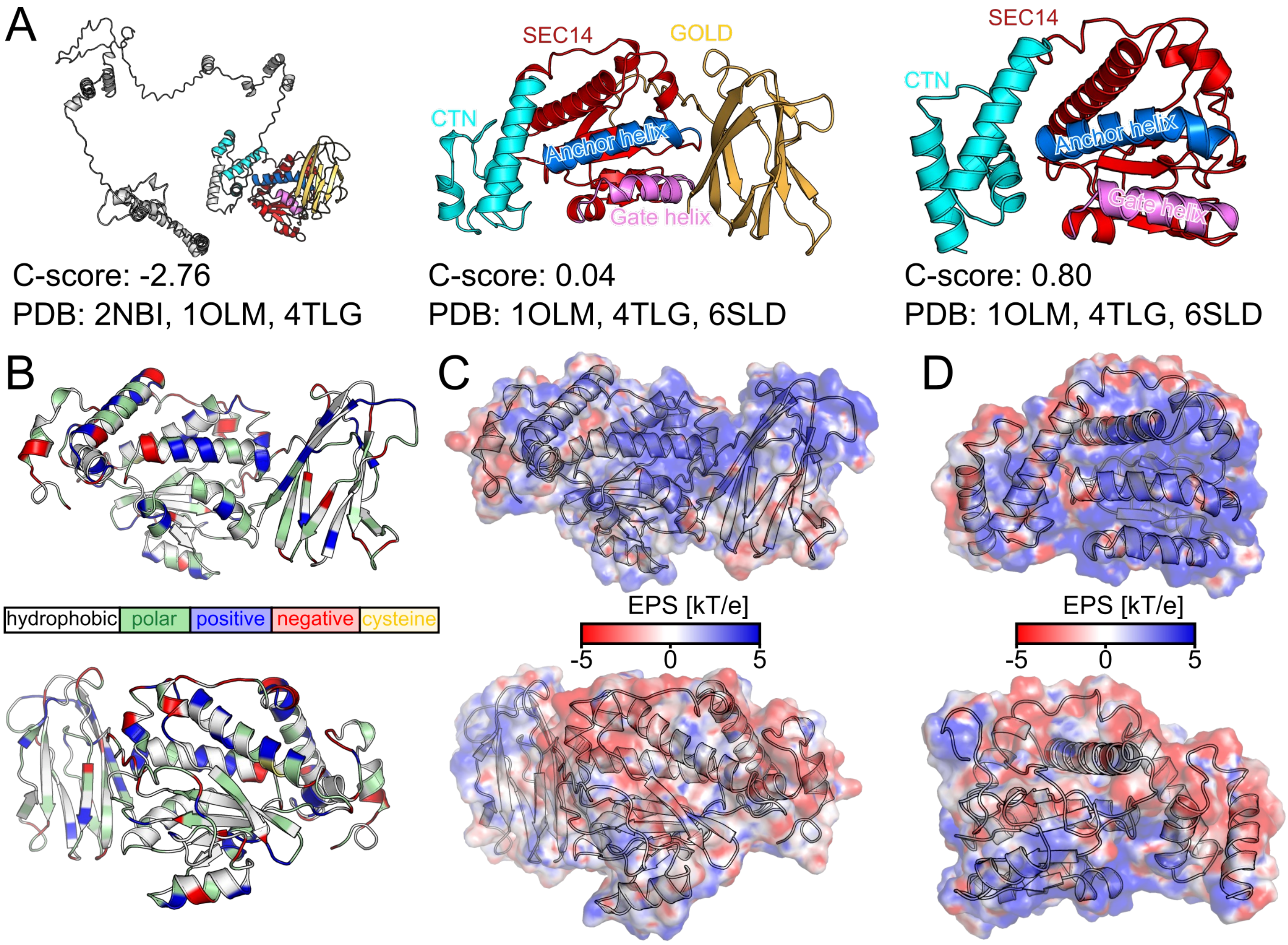

Supplement Figure S12: Additional information for molecular simulation: Homology modeling via I-TASSER and structural analysis of protein models (A) Protein homology models, left, full-length PATL2 (model 5), middle, CTN-SEC14-GOLD part of PATL2 (model 1, CTN-SEC14-GOLD) and right CTN-SEC14 part of PATL2 (model 1, CTN-SEC14). Protein database identification entries (code names 2NBI, 1OLM, 4TLG and 6SLD, H.M. Berman, J. Westbrook, Z. Feng, G. Gilliland, T.N. Bhat, H. Weissig, I.N. Shindyalov, P.E. Bourne (2000) The Protein Data Bank Nucleic Acids Research, 28: 235-242) of high-ranked protein data bank templates were used for the homology modeling. The C-scores were between -5 and +2, whereby a higher C-score corresponds to a higher structural confidence. The different domains of PATL2 are labeled and colored, cyan, CTN; red, SEC14; orange, GOLD; blue, anchor helix; pink, gate helix. Only CTN-SEC14-GOLD and CTN-SEC14 were used for further analysis. (B-D) Protein structures visualized according to residue type analysis; upper, front view; lower, back view, 180° rotated; (B, C) CTN-SEC14-GOLD; (D) CTN-SEC14. In (B) residues are colored using *color_by_residuetype.py* in PyMOL; white, hydrophobic; green, polar; blue, positively charged; red, negatively charged; yellow, cysteine. In (C, D) the protein surface is colored based on the electrostatic potential surface (EPS), between -5 (negatively charged, red) and +5 kT/e (positively charged, blue), calculated with the Adaptive Poisson–Boltzmann Solver (APBS) tool.

Supplemental Figure S13

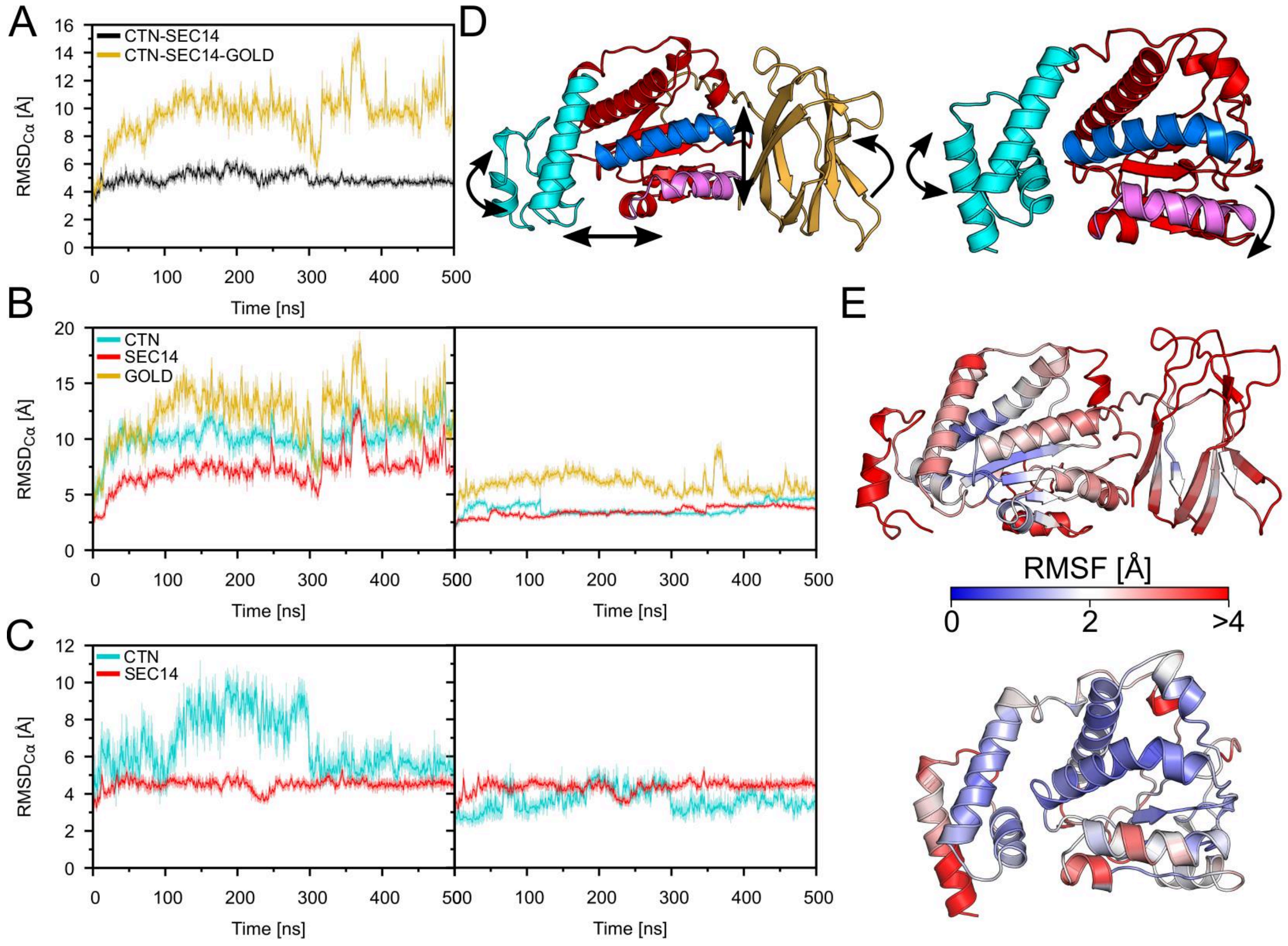

Supplement Figure S13: Additional information for molecular simulation: Analysis of 500-ns MD simulations

Simulations were run for CTN-SEC14-GOLD and CTN-SEC14 protein models. The root mean square deviation (RMSD) values based on Cα atoms serve to assess overall structural changes, while the root mean square fluctuation (RMSF) values highlight the flexibilities of the residues. RMSD analyses were performed for (A) the whole CTN-SEC14-GOLD and CTN-SEC14 proteins; for (B, C) individual domains (as indicated by colors) of the (B) CTN-SEC14-GOLD and (C) CTN-SEC14 proteins. In (B, C) the left panels indicate the RMSD values after aligning the whole protein structures to obtain overall changes such as domain movements, while the right panels indicate the RMSD values after aligning the three individual domains to their start structures, revealing structural changes within the domains. For all RMSD plots, the solid lines show the running average and the shades illustrate the raw data. (D) Schematic illustration of the motions of the individual CTN and GOLD domains, reflected by larger RMSD values, whereby motions of the N-terminal helix of the CTN domain, as well as structural changes surrounding the anchor helix of the SEC14 domain become stabilized upon α-tocopherol binding. Motions are indicated by arrows. The different domains of PATL2 are labeled and colored, cyan, CTN; red, SEC14; orange, GOLD; blue, anchor helix; pink, gate helix. (E) RMSF values of each residue projected onto the homology models, visualizing the different structural stabilities; blue, rigid residues (RMSF < 2 Å); red, flexible residues (RMSF > 2 Å), according to the color scale.

Supplemental Figure S14

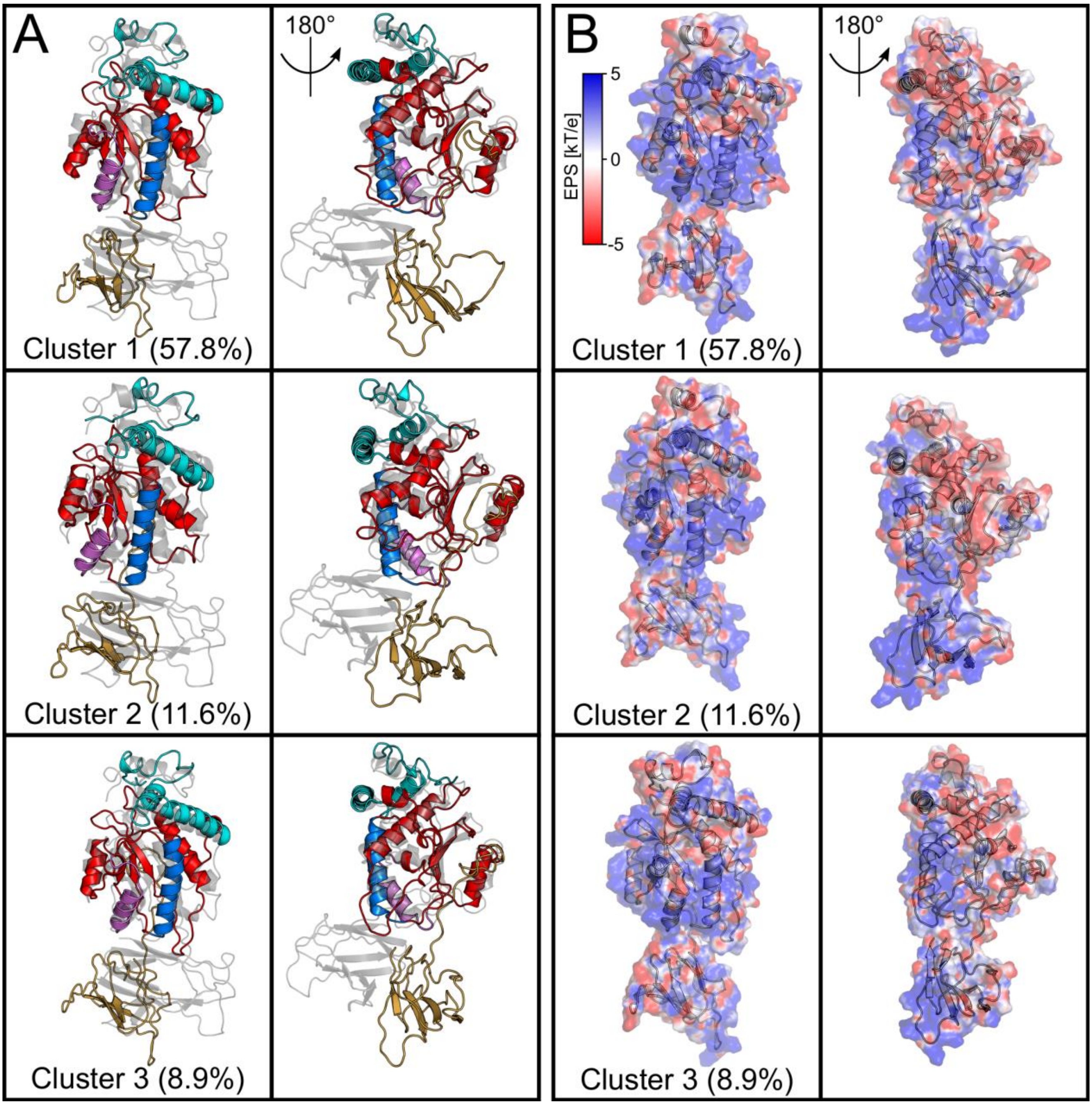

Supplemental Figure S14: Additional information for molecular simulation: Clustering analysis and electrostatic potential surface (EPS) for the CTN-SEC14-GOLD protein model

(A) The first three structural clusters according to population among 5000 simulation snapshots, representing 78.3 % of these snapshots, as obtained from a clustering analysis. Clustering was performed based on the RMSD between snapshots and with a cutoff value of 3 Å to assign cluster membership. The different domains are colored, cyan, CTN; red, SEC14; orange, GOLD; blue, anchor helix; pink, gate helix. The homology model used as starting structure for the MD simulations is shown as gray cartoon. (B) The EPS analysis of the three major clusters, between -5 (negatively charged) and +5 kT/e (positively charged), calculated with the Adaptive Poisson–Boltzmann Solver (APBS) tool 3.0.0.

Supplemental Figure S15

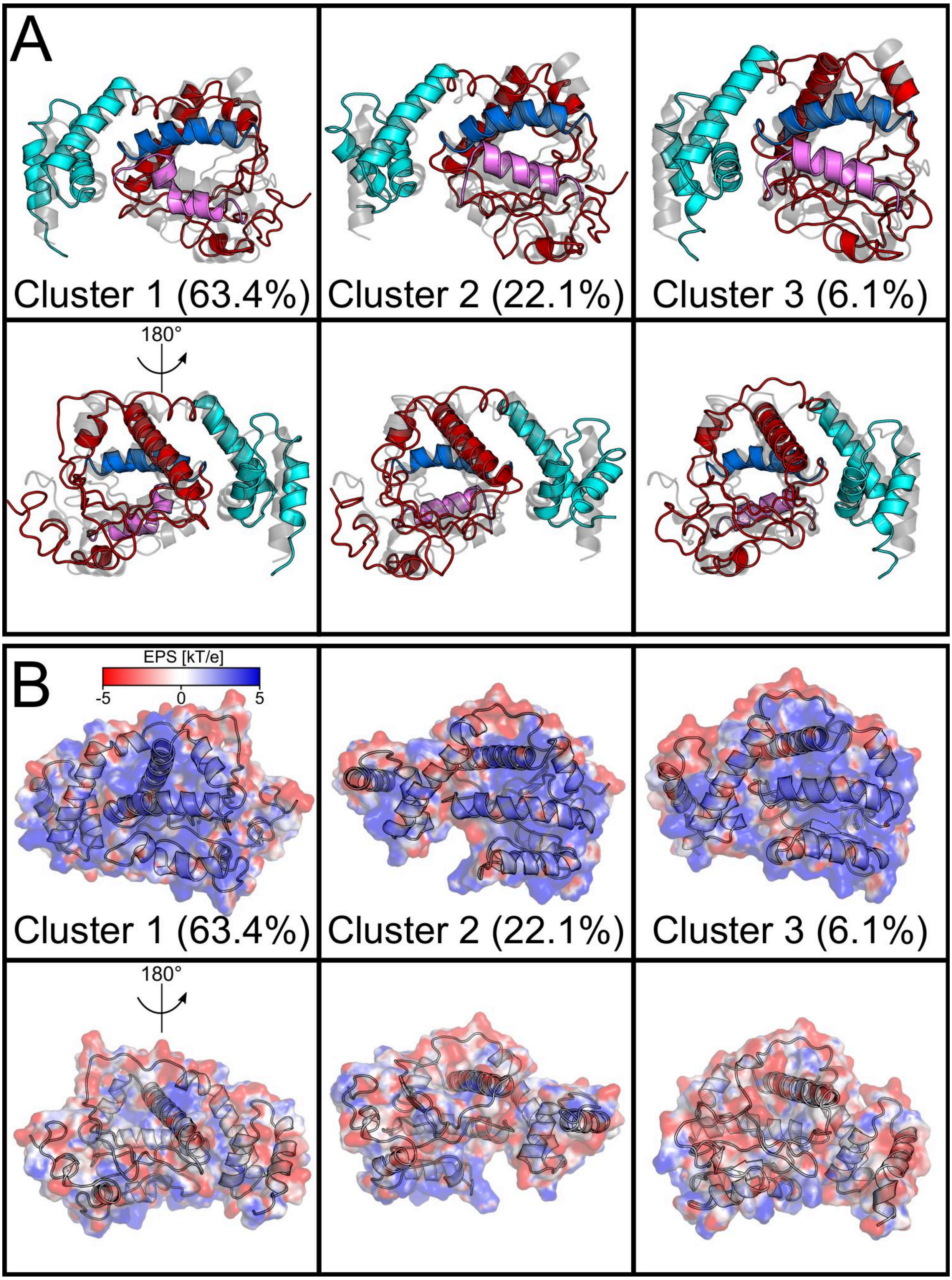

Supplemental Figure S15: Additional information for molecular simulation: Clustering analysis and electrostatic surface potential (ESP) for the CTN-SEC14 protein model

(A) The first three structural clusters according to population among 5000 simulation snapshots, representing 91.6 % of these snapshots. Clustering was performed based on the RMSD values between snapshots and with a cutoff value of 3 Å to assign cluster membership. The different domains are colored; cyan, CTN; red, SEC14; blue, anchor helix; pink, gate helix. The homology model used as starting structure for the MD simulations is shown as gray cartoon. (B) The EPS analysis of the three major clusters, between -5 (negatively charged) and +5 kT/e (positively charged), calculated with the Adaptive Poisson–Boltzmann Solver (APBS) tool 3.0.0.

Supplemental Figure S16

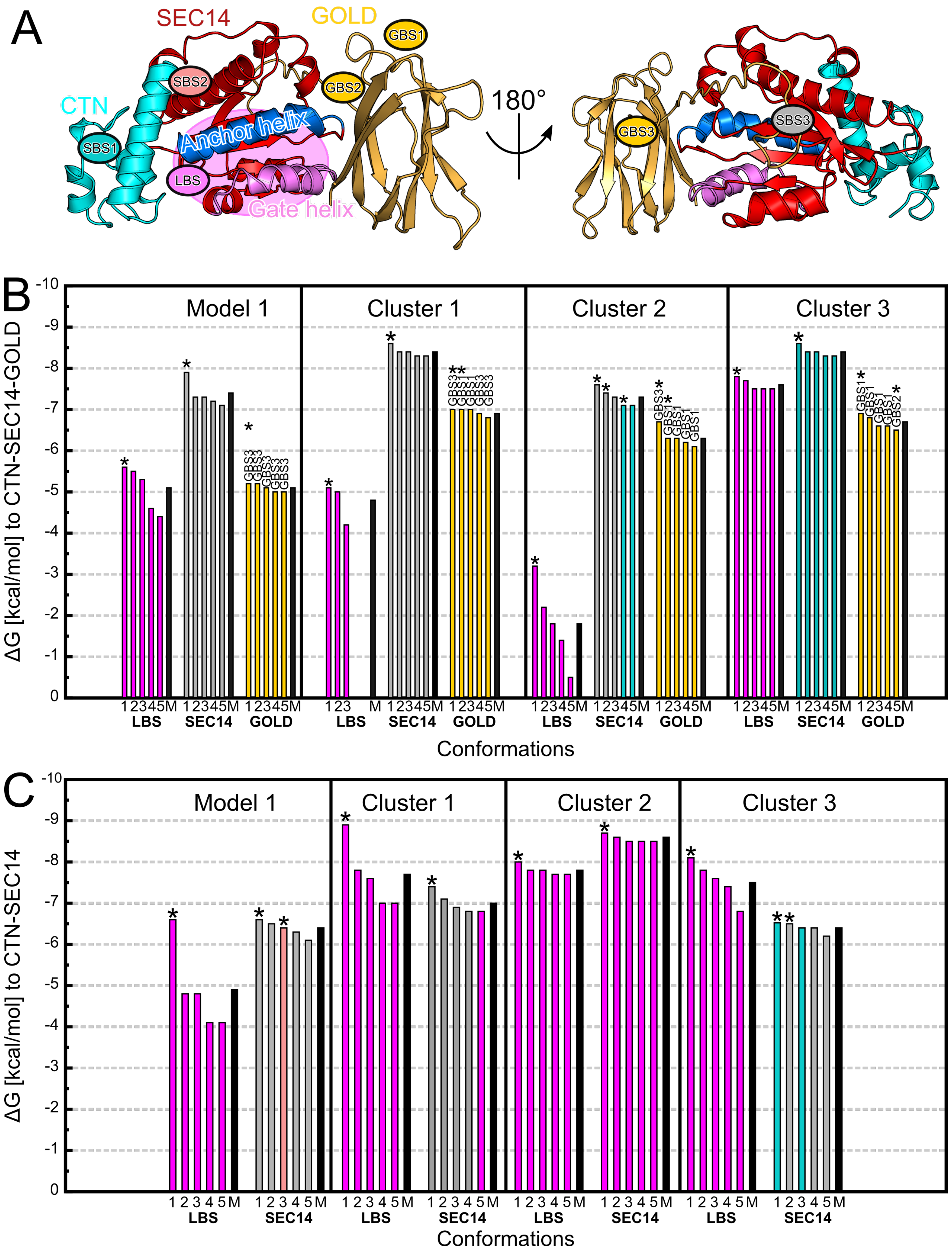

Supplement Figure S16: Additional information for molecular simulation: Summary of docking results for α-tocopherol to PATL2-CTN-SEC14-GOLD and PATL2-CTN-SEC14.

(A) Homology model 1 of PATL2-CTN-SEC14-GOLD with the seven best α-tocopherol-binding sites identified by molecular docking; left, view of protein side oriented towards the membrane and right, 180° rotated view of protein side oriented towards the cytoplasm (compare with Supplemental Figure S12). Different domains and relevant helices are marked in color; blue, CTN; red, SEC14; gold, GOLD domain; pink, gate helix; dark blue, anchor helix. The seven best α-tocopherol binding sites are LBS, lipid-binding site inside the SEC14 lipid-binding pocket; SBS2, SBS3, binding sites at the CTN-SEC14 domain (SEC14-binding site, SBS); GBS1, GBS2, GBS3, binding sites at the GOLD domain (GOLD-binding site, GBS). (B), (C) Bar charts of binding energies (ΔG) of the five best α-tocopherol conformations (bars labeled 1, 2, 3, 4 and 5) for the different docking areas (LBS, SEC14 (SBS) and GOLD (GBS) domain) of (B) PATL2-CTN-SEC14-GOLD, (C) PATL2-CTN-SEC14, represented in ascending order; color code corresponding to (A). (B, C) Highest negative binding energy values indicate best binding modes; the black bar labeled M represents the mean of the corresponding five energies. * indicates the conformations chosen for the 100 ns MD simulations, resulting in 17 simulations for A and 10 simulations for B, with at least one conformation for each binding site and each protein conformation (homology model 1, MD clusters 1, 2 and 3) being selected.

Supplemental Figure S17

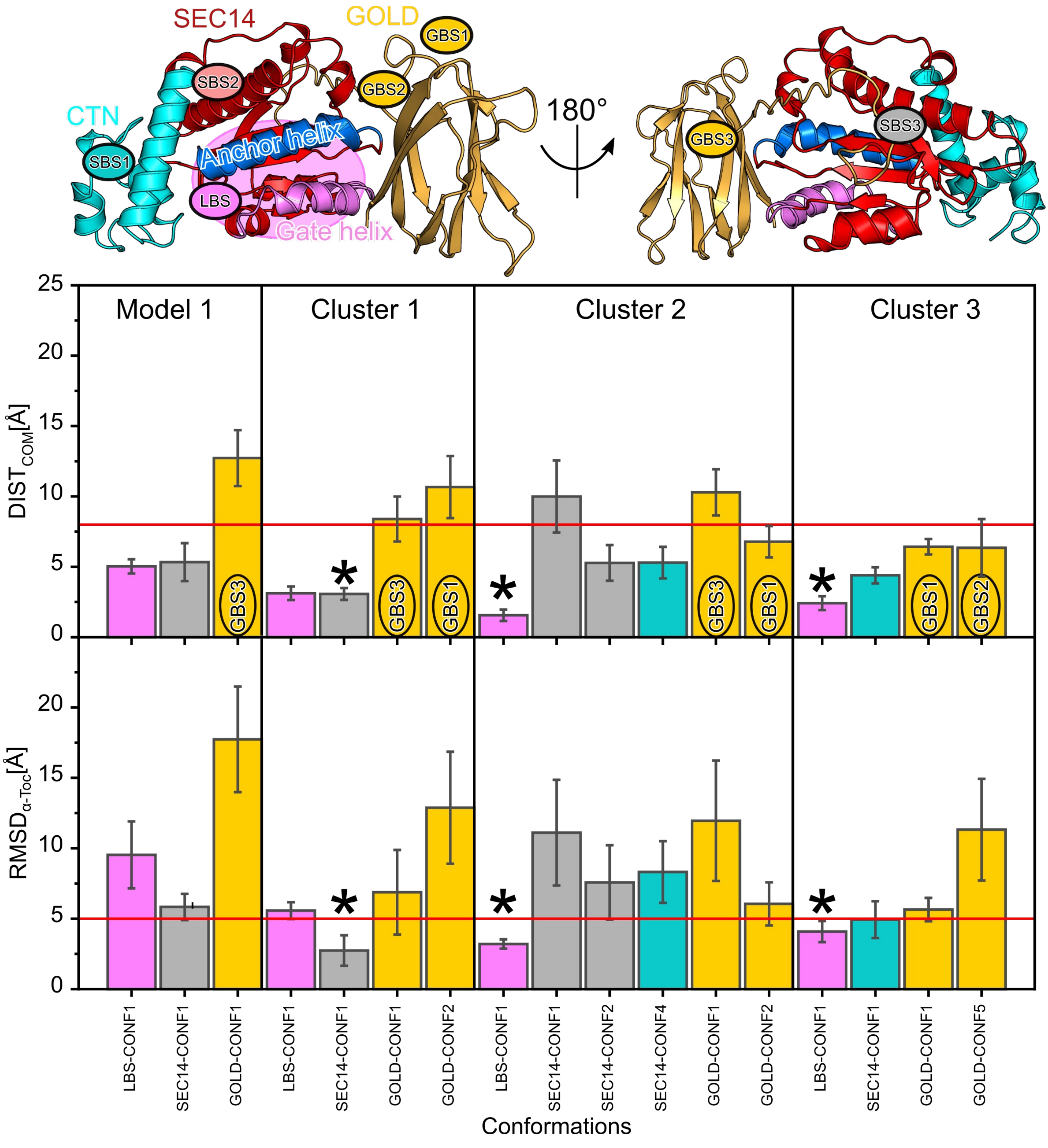

Supplement Figure S17: Additional information for molecular simulation: Molecular dynamics (MD) simulations of the CTN-SEC14-GOLD α-tocopherol (α-Toc) complexes obtained from ensemble docking.
Upper panel, homology model 1 with the different binding sites being indicated; lipid-binding site (LBS); SEC14-binding site (SBS); GOLD-binding site (GBS). The different domains are labeled and colored, cyan, CTN; red, SEC14; orange, GOLD; blue, anchor helix; pink, gate helix. Middle panel, average distance ($DIST_{COM}$) between the centers of mass of α-Toc and the different binding sites, and lower panel, average mean root mean square deviation of α-Toc in the respective binding site ($RMSD_{\alpha\text{-Toc}}$). Results are shown for each of the 17 × 100 ns MD simulations. The α-Toc conformations on the x-axes are labeled according to the docking area (LBS, SEC14 or GOLD) and the docking conformation number (CONF1-5). The color of the bars corresponds to that of the binding site in the upper panel. The red lines indicate the criteria $DIST_{COM} \leq 8$ Å and $RMSD_{\alpha\text{-Toc}} \leq 5$ Å applied to identify stable binding modes, marked by *. Conformation, CONF. The error bars indicate the standard deviation (SD), which is based on a total of 100 ns MD simulations by using n = 10,000 datapoints.

Supplemental Figure S18

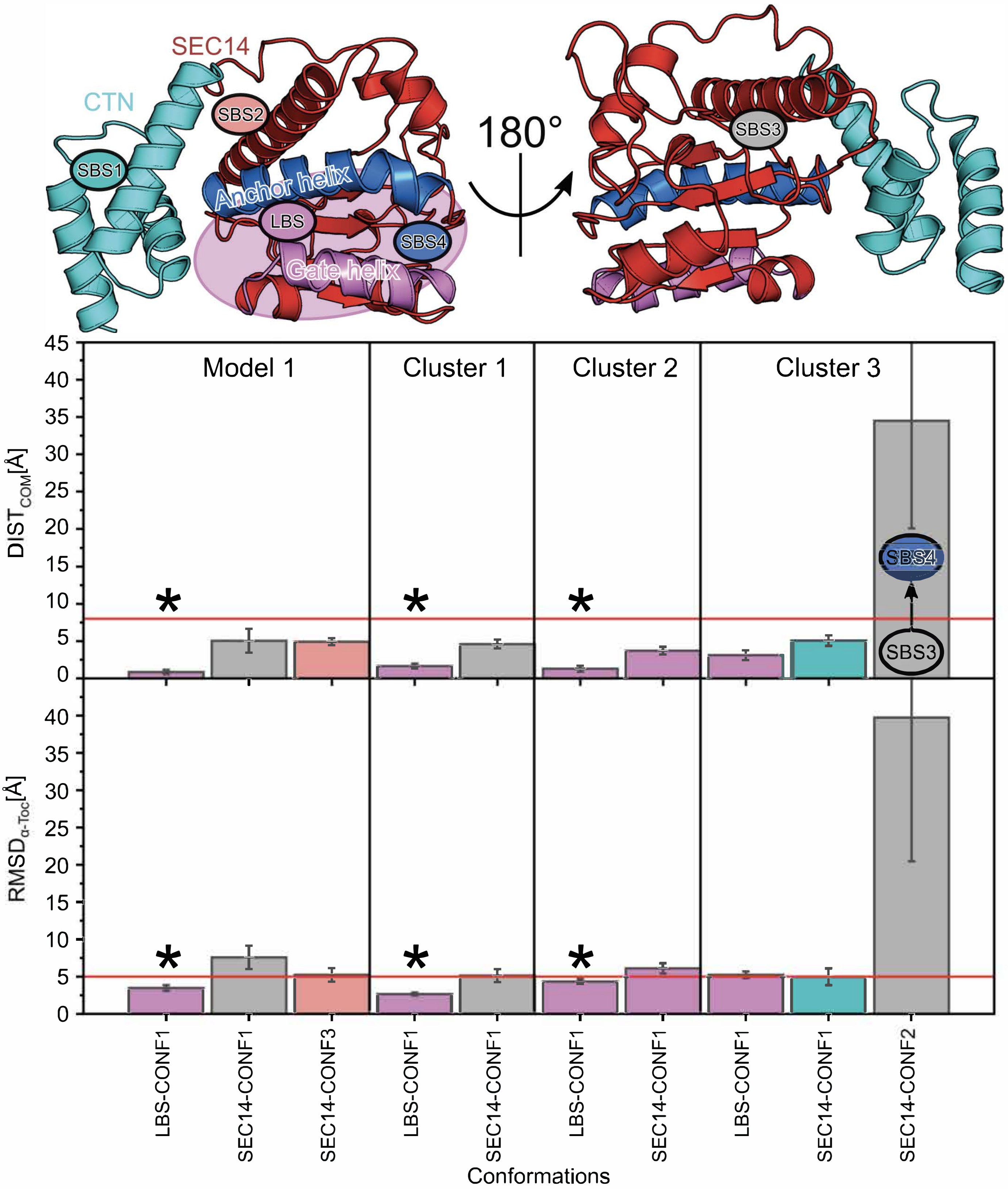

Supplement Figure S18: Additional information for molecular simulation: Molecular dynamics (MD) simulation of ensemble docking results of the CTN-SEC14 model and α-tocopherol (α-Toc).

Upper panel, model 1 with different binding sites (BS); lipid-binding site (LBS); SEC14-binding site (SBS), identified by ensemble docking, in the front and 180°-rotated back view. The different domains are labeled and colored, cyan, CTN; red, SEC14; blue, anchor helix; pink, gate helix. Middle panel, calculated mean center of mass (COM) distance ($DIST_{COM,mean}$) between α-Toc and the BS, and lower panel, mean root mean square deviation of α-Toc ($RMSD_{mean}$) based on 17 × 100 ns MD simulations, represented each for the protein conformations of model 1 and the three clusters obtained (see Supplemental Figure S15). The α-Toc conformations are labeled according to the docking area (LBS, SEC14) and the docking conformation number (CONF1-5). Red lines, selected criteria $DIST_{COM,mean} \leq$ 8 Å (middle panel) and $RMSD_{mean} \leq 5$ Å (lower panel), fulfilled by conformations marked by *. The color of the bar plots corresponds to that of the BS position, see upper panel. Conformation, CONF. Note that in the SEC14-CONF2–state the ligand dissociated from SBS3 towards SBS4. The error bars indicate the standard deviation (SD), which is based on a total of 100 ns MD simulations by using n = 10,000 datapoints.

Supplemental Figure S19

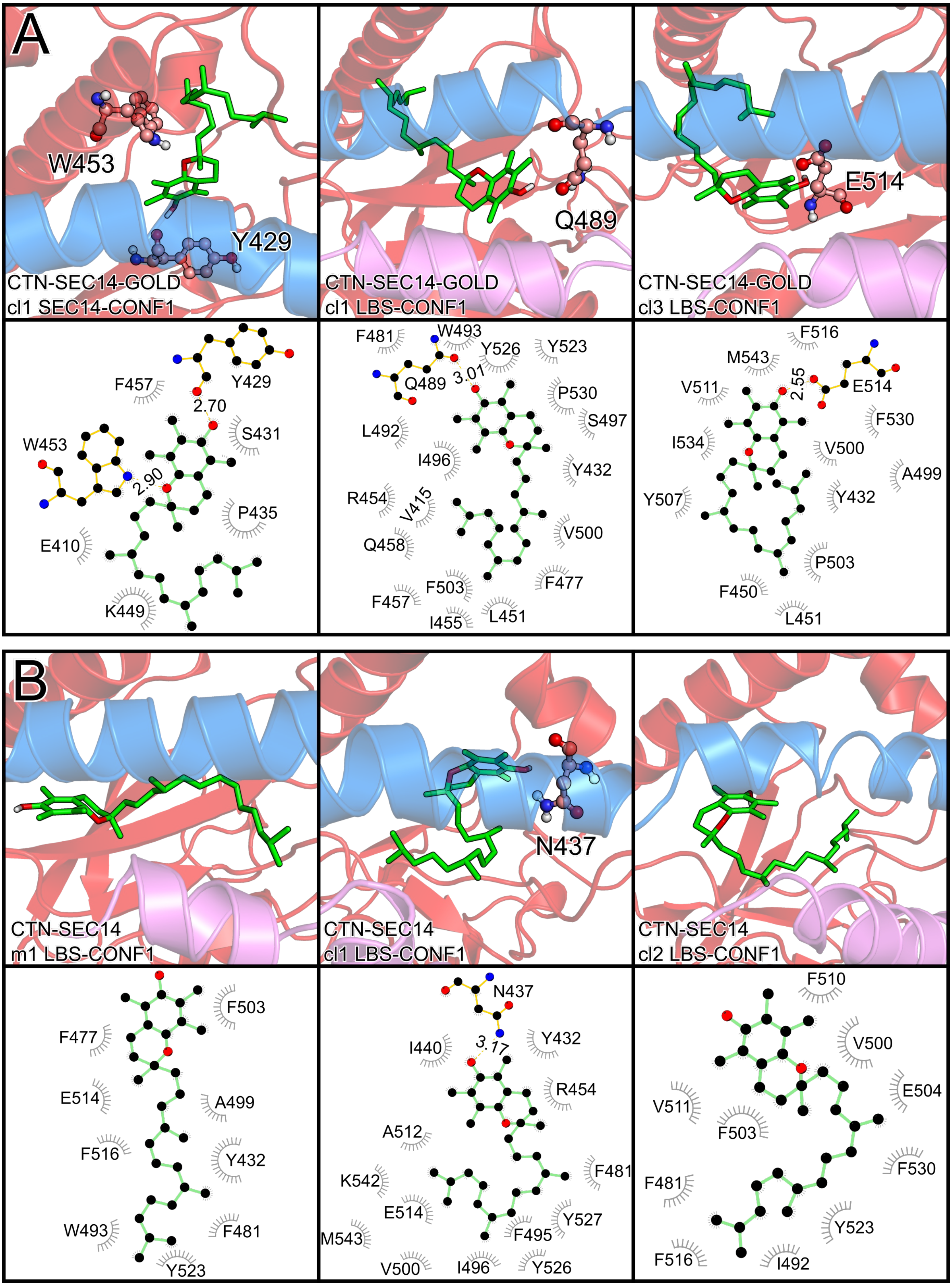

Supplemental Figure S19: Additional information for molecular simulation: Best three binding modes of α-tocopherol (α-Toc) to binding sites of the CTN-SEC14-GOLD and CTN-SEC14 models

The best three binding modes of α-Toc to binding sites of (A) CTN-SEC14-GOLD and (B) CTN-SEC14, labeled by * in Supplemental Figure S17 and S18, respectively, are shown. The upper panels in (A) and (B) show the 3D configurations of the binding, where the proteins are depicted as in the upper panels of Supplemental Figures S17 and S18, while α-Toc is shown as green sticks and the highlighted residues as ball-and-stick models. The side chains of these residues form a hydrogen bond with the hydroxyl group of α-Toc. The bottom panels of (A) and (B) show the interactions between the protein and α-Toc, as determined and visualized by Ligplot+; gray half-circles, residues with hydrophobic interactions; orange, residues with salt bridges or hydrogen bonds, together with distance values between hydrogen donors and acceptors; green, connection to the ligand. The names of the labels reflect the protein model (CTN-SEC14-GOLD or CTN-SEC14), the respective protein conformation (m1, cl1, cl2 or cl3, according to Supplemental Figures S17, S18), the docking areas (LBS, SEC14 or GOLD) and the α-Toc conformation in the docking (CONF1-5 for α-Toc). Note that CTN-SEC14-GOLD–cl2–LBS–CONF1 conformation 1 of the docking between CTN-SEC14-GOLD-cl2 and α- Toc in LBS is reproduced as example in Figure 5F.

Supplemental Figure S20

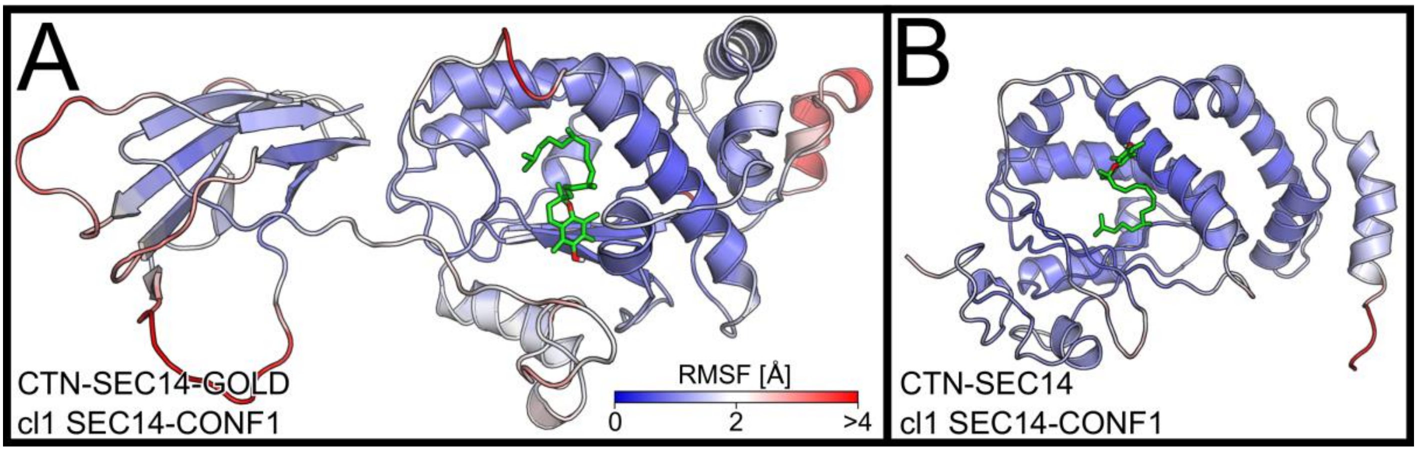

Supplement Figure S20: Additional information for molecular simulation: The potential "allosteric" binding mode of α-tocopherol

The root mean square fluctuation (RMSF) values, representing the residue flexibilities, were projected onto the initial protein structures of (A) PATL2-CTN-SEC14-GOLD and (B) PATL2-CTN-SEC14 for α-tocopherol (α-Toc) in the SEC14-CONF1 at the cluster 1 (cl1) structure, respectively; configuration, CONF. The color code indicates in blue, rigid residues (< 2 Å); red, flexible residues (> 2 Å). α-Toc is shown as green sticks. The binding site shows a stabilizing effect on both PATL2 structures.
