# Supplementary material for: SEC14-GOLD protein PATELLIN2 binds IRON-REGULATED TRANSPORTER1 linking root iron uptake to vitamin E
Source: Plant Physiol. 2022 Dec 9;192(1):504–26. doi: 10.1093/plphys/kiac563 (PMC10152663; doi:10.1093/plphys/kiac563)

Supplemental file

Materials and Methods – Supplementary information

Yeast Two-Hybrid (Y2H) Assay

PATL2 was identified as IRT1vr interaction partner using an activation domain (AD)-IRT1vr fusion expressed from pGBKT7-GW vector as bait in a Y2H screen against a cDNA expression library expressed from pGAT7 vector prepared from Fe-deficient Arabidopsis roots, resulting in binding domain (BD)-fusion prey proteins, as described in (Khan et al., 2019). 43 of the colonies grown on triple selection medium supplemented with 4 mM 3-amino-1,2,4-triazole (3-AT) after day five were tested for potential IRT1vr interactors by amplifying insertion fragments in the pGADT7 vector by PCR with primers Yeast secF and Yeast seqR (Supplemental Table S6). Following agarose gel separation, representative amplification fragments were sequenced for seven clones, one of them contained *PATL2* sequences. For verification of identified IRT1vr-PATL2 interaction an independent targeted Y2H assay was performed according to (Khan et al., 2019). Pairs of pGBKT7-GW and pACT2-GW vectors containing IRT1vr, full-length PATL2 or PATL2 deletion derivatives were co-introduced into yeast strain AH109 and spotted on a double-selective SD medium lacking tryptophane (selects for pGBKT7-GW) and leucine (selects for pACT2-GW), pH 5.8, and SD medium lacking tryptophane, leucine and histidine, and supplemented with 0.5 mM 3-AT, pH 5.8. Absence of yeast colony formation indicating no interaction or positive colony growth indicative of protein-protein interaction were pictured after 4 days of incubation at 30°C.

Bimolecular Fluorescence Complementation (BiFC)

The 2in1 pBiFC-2in1 vector system (Grefen and Blatt, 2012) was used to verify protein-protein interactions in transiently-transformed *Nicotiana benthamiana* epidermis cells. IRT1vr coding sequence starting with an artificial ATG codon and lacking a stop codon was amplified using primers I1LB4 and I1LB1 (Supplemental Table S6) for subcloning into vector pDONR221-B4B1. PATL2 and PATL2ΔN, PATL2ΔC, PATL2ΔCTN, PATL2ΔCTN-SEC14, PATL2ΔSEC14 and PATL2ΔGOLD fragments lacking stop codons were amplified with primers compatible with vector pDONR221-B3B2 (Supplemental Table S6). The generation of IRT1vr, PATL2ΔN, PATL2ΔC, PATL2ΔCTN, PATL2ΔCTN-SEC14, PATL2ΔSEC14 and PATL2ΔGOLD deletion fragments was described previously (Khan et al. 2019; Montag et al. 2020). Following the Gateway cloning procedure (Invitrogen), the fragments were transferred to pBiFC-2in1-CC vector, thus achieving combinations of IRT1vr and one of the PATL2 clones.

Finalized pBiFC-2in1-CC vectors were introduced into *N. benthamiana* through *Agrobacterium tumefaciens* C58C1 (pTiB6S3ΔT)^{H}-mediated transient leaf transformation, as described (Khan et al., 2019). The RFP signal encoded by the vector irrespectively of the BiFC

success was an indicator for a successful transformation event. YFP signal indicated protein-protein interaction of proteins tagged to YFP forms. Confocal images of fluorescent signals were collected as described below.

Protein Co-Immunoprecipitation

PATL2 coding sequence was cloned into pAUL1 (Lyska et al., 2013) to express PATL2-HA_3 protein. *N. benthamiana* leaves were transformed as described above to express combinations of IRT1-GFP and PATL2-HA_3. The plant material was ground in liquid nitrogen and directly resuspended in immunoprecipitation (IP) buffer (50 mM Tris HCl, pH 8.0, 150 mM NaCl, 1 mM EDTA, 1 % (w/v) Triton-X-100, 1 x cOmpete Mini Protease Inhibitor, Roche). Cell debris were removed by centrifugation for 10 minutes at 16.000*g* and 4°C. Part of the sample was kept as input control for SDS-PAGE. GFP-Trap M beads (ChromoTek) were washed in IP Buffer and resuspended to a final volume of 25 µl/reaction. GFP-Trap_M beads were added to the supernatant and incubated by rotating for 2 hours at 21°C. Beads were collected and washed three times with 1 ml IP buffer. Protein elution was performed using 50 µl SDG buffer (62 mM Tris-HCl, pH 8.6, 2.5 % (w/v) SDS, 2 % (w/v) DTT and 10 % (v/v) glycerol) at 95°C for 10 min. Input and elution fractions were separated on a 10 % SDS-PAGE followed by blotting on Amersham™ Protran™ 0.2 µm nitrocellulose membrane (GE Healthcare Life Sciences). PATL2-HA and IRT1-GFP were detected via immunoblot. The membrane was blocked with 5 % milk-TBST (5 % w/w fatty acid-free milk powder, 150 mM NaCl, 2.7 mM KCl, 24.7 mM Tris-HCl, 0.05 % v/v Tween-20, pH 7.4) for 15 min at room temperature, followed by 1h incubation with the specific antibody in 2.5 % milk-TBST. The membrane was washed with TBST and incubated with secondary antibody for 1h. After washing with TBST, signals were detected by enhanced chemiluminescence (GE Healthcare Life Sciences). Antibodies used: anti-IRT1 (AS11 1780; Agrisera, 1:5000); goat anti-rabbit IgG horseradish peroxidase (AS09 602; Agrisera, 1:5000); rat monoclonal anti-HA horseradish peroxidase conjugated (3F10 671; Roche, 1:5000); anti-HA high affinity (11867423001; Sigma-Aldrich, 1:1000). IRT1-GFP had been localized at the plasma membrane and used for Co-IP studies to pull-down IRT1-interacting HA_3-EHB1, but not a non-interacting plasma membrane-associated mutant version of EHB1 (Khan et al. 2019). Primers for molecular cloning are listed in Supplemental Table S6. Additional information is available in Supplemental File 1.

Plant Material and Growth Conditions

Mutant lines *patl2-1* (SALK_086866), *patl2-2* (SALK_009882), *patl1-1* (Salk_080201) and *vte2-2* (Havaux et al., 2005), in *Arabidopsis thaliana* Columbia-0 (Col-0, wild type) ecotype background, were used in this study. *patl1-1 patl2-2* double knock-out mutant were generated by crossing. The PATL2-HA_3 construct was generated by amplifying *PATL2* coding sequence,

subcloning the fragment into pDONR207 and transferring to pAUL1 (Lyska et al., 2013). For the generation of the PATL2-HA_3 pAUL1:PATL2 (2xpro35S, pro35S::PATL2-HA_3) was used for transforming *A. tumefaciens* C58C1 (pTiB6S3ΔT)^H strain. Transformation of Col-0 and *patl2* mutants was performed using floral dipping (Clough and Bent, 1998). Dipped plants were selected for positive transformation and homozygous T4 generations were used in this work. *PATL1* and *PATL2* promoter::GUS transcriptional fusions were generated by amplifying 1000 bp fragments form the respective upstream region (Supplemental Table S6). The fragments introduced into pMDC107 vector (Curtis and Grossniklaus, 2003), which was transferred to *A. tumefaciens* and used for Arabidopsis transformation by floral dipping. The pIRT1::GUS line was previously described (Vert et al., 2002).

Physiological studies

Arabidopsis seeds were surface-sterilized for 8 min in sterilization solution (6 % NaClO and 0.1 % Triton X-100) and grown upright on Hoagland medium agar plates (Khan et al., 2019). In the “14+3 system”, 14-day-old plants were grown on 50 µM Fe medium and after 14 d transferred to new Hoagland medium that was either Fe-sufficient containing 50 µM FeNaEDTA (50 µM Fe) or Fe-deficient with no FeNaEDTA (0 µM Fe) for three days. In the “6- or 10-day system” seedlings were directly grown on 50 µM Fe or 0 µM Fe Hoagland plates for six days. Plants were grown at 20°C in a 16h-light/8h-dark rhythm in climate chambers (CLF PlantClimatics). For seed harvesting, plants were grown in soil in the greenhouse. *Nicotiana benthamiana* plants were grown on soil for four weeks before transformation. Root length measurements were taken from seedlings grown in the 6-day system using ImageJ as previously described (Khan et al. 2019). Soil plant analysis development (SPAD) values, a relative indication of chlorophyll contents, were determined in leaves of plants grown in the 14+3 system using SPAD 502 Plus Chlorophyll Meter (Spectrum Technologies, Inc.).

Localization and Co-localization of fluorescence protein reporter fusion proteins

For co-localization experiments, constructs expressing YFP-PATL2 (Montag et al., 2020), IRT1-mCherry (Ivanov et al., 2014) and Lti6b-mRFP (Caesar et al., 2011) were transiently expressed in *N. benthamina* leaf epidermis cells. YFP-PATL2 was previously shown to localize to the plasma membrane (Montag et al. 2020) in agreement with immunolocalization of PATL1 and PATL2 in plant cells (Peterman et al. 2004). IRT1-mCherry was previously shown to co-localize with IRT1-GFP and early endosome proteins (Ivanov et al 2014) and with EHB1-GFP at the plasma membrane (Khan et al. 2019). These previous localization patterns corresponded with the localization of GFP-PATL2 (Tejos et al., 2018) and with that of IRT1-mCitrine (Dubeaux et al. 2018).

For root cell localization of IRT1-mCitrine and GFP-PATL2, the lines proIRT1::IRT1-mCitrine/*irt1* (Dubeaux et al. 2019) and proPATL2::GFP-PATL2 (Tejos et al. 2018) were grown for 6 days directly on + and -Fe medium prior to investigation of the root hair zone using the LSM780 confocal setup, described above. Z-stacks were taken through the root to generate a dissection in x-z dimensions as indicated in the figure legends.

Confocal images of fluorescent signals were collected using the LSM780 system (Zeiss, Germany). YFP and mCitrine signals were excited at 514 nm, and emission was detected at 520–550 nm. mRFP and mCherry were excited at 561 nm, and emission was detected at 580–630 nm. GFP signals were excited at 488 nm, and emission was detected at 510–540 nm.

Histochemical β-Glucuronidase Staining

PATL1 promoter::GUS, *PATL2* promoter::GUS and *IRT1* promoter::GUS seedlings were grown on Hoagland plates for 8 days. Seedlings were incubated in GUS staining solution (50 mM sodium phosphate, 2 mM potassium ferrocyanide, 2 mM potassium ferricyanide, 0.2 % Triton X-100, and 2 mM GUS substrate 5-bromo-4-chloro-3-indolyl-b-D-glucuronic acid) (Jefferson et al., 1987). Stained seedlings were cleared in 70% ethanol. Pictures of seedlings were taken using Zeiss Axio Imager M2 microscope at 20x magnification using the “Tiles” module and the “stitching” function of the software ZEN 2 (Zeiss) to assemble the collected images. Three independent lines were analyzed for each pPATL1::GUS and pPATL2::GUS construct. Representative results are shown.

Plant Immunoblot

Protein was extracted from ground plant tissue with 2x SDG (4 % SDS w/v, 0.2M DTT, 20 % glycerol v/v, 0.02 % bromophenol blue, 1 µl per mg tissue). After boiling for 10 minutes, 5 µl were separated on a discontinuous 12 % SDS-polyacrylamide gel and proteins transferred to a 0.2 µm nitrocellulose membrane (Amersham Protran 0.2 NC nitrocellulose Western blotting membranes, Cytiva). Immunoblot analysis was performed with rabbit anti-IRT1 IgG (αIRT1, Agrisera AS11 1780) or rabbit anti-FER IgG (αFER, Agrisera AS10 674), each diluted 1:5000 in 2.5% Milk-TBST buffer, secondary goat anti-rabbit IgG, HRP conjugate (Agrisera AS09 602, diluted 1:10,000 in 2.5% Milk-TBST) and rabbit anti-ACTIN (ACT) (Agrisera AS13 2640). Signals of enhanced chemiluminescence reaction (with Amersham™ ECL™ Select Western Blotting Detection Reagent, Cytiva) were detected by FluorChem Q (proteinsimple™) and analyzed with AlphaView® software (proteinsimple™). Background-corrected signals were normalized to prominent bands of of ACT immunostaining.

Gene Expression Analysis by reverse transcription (RT)-qPCR

RT-qPCR was performed according to (Ben Abdallah and Bauer, 2016). Each biological replicate contained roots of 12 plants. Total RNA was isolated using the peqGOLD Plant RNA Kit (PeqLab). For cDNA preparation Oligo dT primer and RevertAid first-strand synthesis kit (Thermo Scientific) were used. The DyNAmo ColorFlash SYBR Green qPCR Kit (Thermo Scientific) was used for qPCR in technical duplicates in the CFX96 Real-Time System (BioRad). Melt curve analysis was performed using the CFX Manager software (BioRad). Absolute quantification was performed using mass standard curve analysis. qPCR primers for Fe response genes are listed in (Naranjo Arcos et al., 2017; Gratz et al., 2019), for *VTE* and *PATL* genes listed in Supplemental Table S6. Gene expression of samples was normalized to expression of reference gene *EF1Bα*.

Root Fe reductase activity assay and determination of Fe content

Root Fe reductase activity was measured spectrophotometrically by the ferrozine method described in (Le et al., 2016), using per sample a pool of five plants in 2 ml reaction solution with 500 µM ferrozine, 100 µM FeNaEDTA and applying for calculation an extinction coefficient for ferrozine-Fe^{2+} of 28.6 $mM^{-1} * cm^{-1}$.

Quantification of Fe content of the dried seeds was determined as previously described (Khan et al. 2019). Seed samples were ground and melted at high temperature in a solution of 65% (v/v) HNO_3 and 30% (v/v) H_2O_2. The cooled solutions were measured with inductively coupled plasma optical emission spectrometry (conical atomizer; 0.61 l/min argon flow; pressure: 2.41 bar, λ_{Fe}=259.940 nm, Ultima 2; HORIBA). Three independent batches of seeds were analysed.

Yeast *fet3 fet4* complementation

For the yeast *fet3 fet4* complementation assay, *IRT1* coding sequence was cloned into vector pAG426GPD-ccdB-eYFP vector (Susan Lindquist, Addgene plasmid # 14228) as previously described (Khan et al., 2019). Coding sequence of *PATL2* was subcloned into pDONR207 and transferred to pAG425GPD-ccdB-HA (Susan Lindquist, Addgene plasmid # 14250). Yeast strains INVSc1 (*MATa his3D1 leu2 trp1-289 ura3-52 MAT his3D1 leu2 trp1-289 ura3-52*, Thermo Fisher Scientific) and DEY1453 (*MATa/MATa ade2/1 canl/canl his3/his3 leu2/leu2 trpl/trpl ura3/ura3 fet3-2::HIS3/fet3-2::HIS3 fet4-1::LEU2/fet4-1::LEU2*; (Eide et al., 1996)) were used as hosts. Primers for molecular cloning are listed in Supplemental Table S6. To study the IRT1-mediated complementation of the DEY1453 (*fet3 fet4*) Fe-deficiency phenotype, 10-fold dilutions of yeast cultures harboring combinations of IRT1 and PATL2, together with control transformants, were plated on agar plates containing yeast extract peptone

dextrose medium either supplemented with 50 mM of Fe chelator bathophenanthrolinedisulfonic acid (BPDS, Fe-depleted condition) or without it (control condition). Serial dilutions of yeast colony growth was observed on day three after plating. An identical experiment in the INVSc1 strain was used to exclude any potential adverse effects to yeast growth from the expression of the constructs in the absence of the Fe uptake-related mutations. Yeast colonies were photographed, the density determined with ImageJ and quantified, as indicated in the figure legend.

Co-Immunoprecipitation-Mass Spectrometry (IP-MS) Analysis of the PATL2 Interactome

The workflow is detailed in Supplemental Figure S6D. PATL2-HA_3 and wild-type seedlings were grown in the 14+3 d system and exposed to 0 µM Fe (-Fe) and 50 µM Fe (+Fe) to harvest a total of 20 root samples, as 5 biological replicates x 2 lines x 2 growth conditions à 150 seedling roots each. Samples were flash-frozen in liquid nitrogen and ground using the Precellys® 24 with Cryolys® cooling unit (VWR) in the presence of 1.4 mm Precellys® zirconium oxide beads (Bertin Corp.) three times for 20 sec. The material was solubilized in 1 ml IP buffer and immunoprecipitation was performed as described above with 25 µl anti-HA magnetic beads (Thermo Scientific, Pierce) with overnight incubation at 4°C. The IP sample was collected along with input and the five wash samples (see example in Supplemental Figure S6C).

The 20 IP samples were briefly separated (~5 mm running distance) in Bis-Tris buffered 4–12 % acrylamide gels (Thermo Fisher Scientific, Waltham, MA, USA). After silver staining and band processing, in-gel digest was performed using trypsin as described in (Grube et al., 2018). Following extraction from the gel, peptide samples were separated on an Ultimate 3000 rapid separation liquid chromatography system (RSLS, Thermo Fisher Scientific) using a 1 h gradient on C18 material basically as described in (Grube et al., 2018). The separated peptides were detected by tandem mass spectrometry in an Orbitrap Fusion Lumos Tribrid mass spectrometer (Thermo Fisher Scientific) operated in data-dependent positive mode and coupled via a nano-electrospray interface. Precursor spectra were recorded at a resolution of 120,000 over a scan range of 200-2000 m/z. Maximum injection time was 50 ms and the AGC target was set to 400000. Subsequently, twofold- to sevenfold-charged precursors were isolated by the build in quadrupole (isolation window: 1.6 m/z) and fragmented via higher-energy collisional dissociation. The fragment spectra were recorded in the linear ion trap (scan range: auto, scan rate: rapid, maximum injection time: 150 ms, AGC target: 10000). Cycle time was 2 seconds and already fragmented precursors were excluded from fragmentation for the next 60 seconds.

Recorded MS data were analyzed using the MaxQuant software (version 1.6.6.0, Max Planck Institute of Biochemistry, Martinsried, Germany) with standard parameters. Searches were

based on entries of the *Arabidopsis thaliana* proteome set (UP000006548, downloaded from the UniProt Knowledgebase on 7th Mai 2019 and one additional entry for HA tagged PATL2). Label-free quantification (LFQ) was enabled and proteins had to be detected by at least two different peptides and four valid values in at least one group. Perseus (version 1.6.6.0, Max Planck Institute of Biochemistry, Martinsried, Germany) was used for further processing. Detected intensities were log_2-transformed and missing values were imputed by replacement with randomized values from the normal distribution (width 0.3 SD, downshift 1.8 SD). Samples were assessed by principle component analysis and following that one sample (WT, +Fe) was removed. Statistical enrichment of IP-MS data from the PATL2-HA_3 compared to the wild type interactome was identified by two-tailed two-sample Student's t-tests (S0 0.1, FDR 0.05) (termed Statistical analysis I). As an additional cut-off method, we used enrichment in > 2 HA_3-PATL2 samples and simultaneously in < 3 wild-type control samples for -Fe and < 2 wild-type control samples for +Fe (termed Statistical analysis II). The protein lists are summarized in Supplemental Table S1. Gene ontology analysis was performed using the Protein Analysis Through Evolutionary Relationships (PANTHER) system's GO tool, www.pantherdb.org (Mi et al., 2021). Protein descriptions were downloaded from The Arabidopsis Information Resource (www.arabidopsis.org). GO term enrichment was conducted and represented as previously described (Kar et al., 2021). The mass spectrometry proteomics data have been deposited to the ProteomeXchange Consortium via the PRIDE [1] partner repository with the dataset identifier PXD032079.

H_2O_2 Measurement and Lipid Peroxidation Assay

The H_2O_2 content was measured using the Amplex® Red Hydrogen Peroxide/Peroxidase Assay Kit (Thermo Fisher Scientific) following the protocol by (Brumbarova et al., 2016).
Lipid peroxidation was determined using the thiobarbituric acid reactive substances (TBARS) assay described in (Zhang and Huang, 2013). 50 mg of roots were shock-frozen and ground. Plant material was homogenized using 0.5 ml of 0.1 % TCA and subjected to 10-min centrifugation at maximum speed. Then, 350 µl supernatant were combined with 1.65 ml TBA solution (0.5 % w/v TBA, 20 % w/v TCA) and incubated for 25 minutes at 95°C. After cooling samples were centrifuged for 5 min at maximum speed (4°C). The supernatant was subjected to absorption determination at 532 nm and 600 nm. The TBARS concentration was determined using the Lambert-Beer-Law and the extinction coefficient of malondialdehyde (MDA; 155 $mM^{-1}cm^{-1}$).

Analysis of Tocopherol Content by gas chromatography (GC)-MS

The tocopherol content was described in (Stahl et al., 2019). In brief, 50 mg shoot and up to 100 mg root material was ground using a nitrogen-cooled Precellys (Bertin Technologies) and

extracted twice with 1 ml $CHCl_3$:MeOH:H_2O (1:2.5:1, v/v/v). For quantification, 1 µg Tocol was used as internal standard (IST). 600 µl extract was evaporated using a SpeedVac (Concentrator plus, Eppendorf) and subjected to silylation using 20 µl pyridine, 20 µl *N*-methyl-*N*-trimethylsilyltrifluoroacetamide containing 1% (v/v) trimethylchlorosilane and 60 µl hexane and incubation at 70°C for 30 min. Shoot samples were diluted 1:1 with hexane and transferred to a GC vial. Root samples were transferred undiluted to GC vials. 2 µl of the sample mixture was separated on a gas chromatograph (GC 7890A; Agilent Technologies) equipped with a fused silica capillary column (Phenomenex ZB-35; 30 m x 0.25mm x 0.25 µm). Mass spectra were recorded in the range between mass-to-charge ratio (m/z) 50 and 750 with a 5975C mass spectrometric detector (Agilent Technologies) in the electron ionization mode. The peak areas corresponding to α-tocopherol (m/z 502), β-tocopherol and γ-tocopherol (m/z 488), δ-tocopherol (m/z 474) were set in relation to the peak area of IST Tocol (m/z 460). The absolute content of tocopherols was calculated using previously established correction factors (Stahl et al., 2019).

Bacterial Protein Expression and Purification of Recombinant PATL2 Protein

Recombinant Strep-tagged proteins (PATL2, PATL2∆GOLD, PATL2∆CTN-SEC14, POI) were expressed in *Escherichia coli* Rosetta2 (DE3) pRARE2 cells using the recombinant POI pET52 vectors described in (Montag et al., 2020). In brief, bacterial cultures containing protein expression plasmids were induced with 0.5 mM IPTG at an OD600 of 0.6-0.8 and grown for 20 hours at 18°C. Cells were harvested by centrifugation and suspended it Buffer W (100 mM Tris-HCl, 650 mM NaCl, 1 mM EDTA, 1x Mini EDTA free protease inhibitor cocktail (Merck), 5 mM DTT, 100 µM PMSF, pH 8.0). Cell disruption was performed by sonication (Digital Sonifier® W-250 D, Branson Ultrasonic Corporation). The soluble fraction was separated by centrifugation (24,000*g*, 1 h, 4°C), incubated with avidin to block native Biotin and cleared of precipitates (centrifugation, 24,000*g*, 30 min, 4°C). The soluble fraction was filtered through a 0.44 µm nitrocellulose filter and subjected to a NGC-System (Biorad) equipped with a 5 ml Strep-Tactin®XT 4Flow® cartridge (iba) for affinity chromatography. The column was washed using high salt and ATP-containing Buffer W (100 mM Tris-HCl, 650 mM NaCl, 1 mM EDTA, 10 mM ATP, 20 mM MgCl3, pH 8.0). The elution fractions were pooled and concentrated using Amicon® Ultra-4 10K centrifugal filter device (Merck) and subsequently subjected to Superdex™ 200 Increase 10/300 GL prepacked Tricorn™ Column (Cytiva) for size exclusion chromatography. Immunoblot validated POI fraction was stored in the fridge for up to one week and used for assays (Supplemental Figure S9A).

NBD-α-tocopherol-PATL2 protein-ligand binding spectrofluorimetric assay

NBD-α-tocopherol (NBD-Toc) was synthesized according to the protocol of (Nava et al., 2006). Successful synthesis was confirmed by ^{1}H NMR und Electron Ionization MS using a Bruker Advance III - 300 in $CDCl_3$ with residual chloroform as an internal reference (7.26 ppm): δ (ppm): 8.51 (d, ^3J = 8.70 Hz, 1H, ArH), 6.18 (d overlapped with br s, ^3J = 8,67 Hz, 2H, ArH & -NH-), 3.48 (q, solvent Et_2O), 2.55 (t, ^3J = 6,89 Hz, 2H, -NH-CH_2-), 2.08 (s, 3H, -CH_3), 2.06 (s, 3H, -CH_3), 2.05 (s, 3H, -CH_3), 1.88-1.72 (m, 6H, -CH_2-), 1.65-1.23 (m, 44H, -$(CH_2)_7$-, solvent hexane & H_2O, 1.21 (s, 3 H, -CH_3), 1.21 (t, solvent Et_2O), 1.06 (s, 9 H, -Si-$(CH_3)_3$), 0.91-0.83 (m, -CH_3 (n-hexane)), 0.12 (s, , 6H, -Si-CH_3); chemical shifts given as δ-values (ppm), coupling constants (J) in hertz (Hz). The abbreviations s, d, t, q, m, Ar, and br are used for singlet, doublet, triplet, quartet, multiplet, aryl and broad. Mass spectrum (MS) was obtained using a Finnigan Trace DSQ spectrometer; MS (EI) m/z: 624 (M+).

Crude NBD-Toc was stored in the fridge under N_2 atmosphere. Working aliquots in 100% EtOH (p.a.) were stored at -20°C and chilled at ambient temperature for half an hour prior to use. NBD-Toc was diluted to a series of 16 concentrations (at 100fold of final assay concentrations) on ice to prevent EtOH evaporation. The protein-ligand binding assay was established according to (Jarmoskaite et al., 2020). *E. coli*-expressed and purified PATL2 and PATL2 variant protein (POI, see previous paragraph) was used, the buffer was changed using PD Mini desalting columns (Cytiva) and diluted to 50 nM in TKE (50 mM Tris-HCl, 100 mM KCl, 1 mM EDTA, pH 7.6). POI was used in the respective concentrations, 2.5 µl of respective NBD-dilution was added to yield the respective final concentrations of NBD-Toc in 1% EtOH (v/v) in a total volume of 250 µl (see Supplemental Figure S9B-F). The POI-NBD-Toc mixture was incubated at 20°C for 3 h and 20 h until reaching of the equilibrium state (see Supplemental Figure S9B-F). Fluorescence measurements were performed using the Infinite M200 pro plate reader (TECAN., λex/em: 469/540 nM, Gain: 135, Z-Position: calculated from well, 25 flashes/read, integration time: 25 µs). The raw values were subtracted from respective background fluorescence (NBD-Toc in TKE, no protein). Finally, the fraction bound (F_{bound}) was calculated using 50 nM protein and division of each replicate value by its highest value (upper equilibrium). The dissociation constant K_D and non-linear fit were calculated using the DoseResponse function in OriginPRO 2021.

Homology modeling and MD Simulations

The workflow is depicted in Supplemental Figure S10.

Obtaining protein structural models (Step I):

A) Homology modeling via I-TASSER: As there was no experimental PATL2 structure available, homology modeling was performed using the I-TASSER web server (Zhang, 2008; Roy et al., 2010; Yang et al., 2015) to predict the tertiary structure of PATL2 (residues 1–683), of PATL2-CTN-SEC14-GOLD (341–683) and PATL2-CTN-SEC14 (CTN-SEC14, 341–581). I-

TASSER uses an iterative approach found reliable in other predictions (https://zhanggroup.org/I-TASSER/). This tool includes different validated algorithms to search the protein database (PDB) library for templates, to use fragments of the template that fit with the sequence alignment, as well as *ab initio* modeling and clustering for obtaining the whole structure and several analysis tools for validation of the five best structures (C-score) and providing ligand-binding information. First, the meta-threading algorithm identified the top ten sequence alignments to targets of similar folds from the PDB. Then, structure models were produced via Monte-Carlo simulations to obtain thousands of structural conformation variants of the target. They were clustered into five representative models ranked by the cluster size and their quality based on the C-score, which ranges between −5 and +2, with a higher C-score corresponding to a high structural confidence. The first model per protein form with best C-score (model 1) was used for molecular dynamics (MD) simulations (Supplemental Figure S11). To determine the electrostatic potential surfaces (EPS) of the proteins, we used the APBS tool 3.0.0 (Jurrus et al., 2018) (https://server.poissonboltzmann.org/).

B) MD simulation protocol and force field parameters for the ligands:

For the MD simulations, the CHARMM36 force field (from July 2020 and February 2021), the CHARMM-modified TIP3P water model and GROMACS 2020 (Abraham et al., 2015; Lindahl et al., 2021) were used. The simulations were run at a temperature of 293 K (20 °C) and a pressure of 1 bar. In Supplemental Table S4, all MD simulations are listed, yielding a total simulation time of 3700 ns.

First, we tested the stability of homology model 1 of PAL2-CTN-SEC14 and PATL2-CTN-SEC14-GOLD, which was placed in a cubic box with a volume of 90 × 90 × 90 $Å^3$ (concentration: 2.28 mM) and 110 × 110 × 110 $Å^3$ (concentration: 1.26 mM), respectively. After solvation with 22,689/41,528 water molecules and 1 Cl^- for PATL2-CTN-SEC14-GOLD (to neutralize the system), a system was created with a total of 72.319/130.581 atoms. Subsequently, the energy was minimized via the steepest descent algorithm, followed by equilibration to the desired temperature of 293 K (20 °C) and pressure of 1 bar for mimicking the physiological environment. To this end, we performed a 0.1 ns equilibration run, keeping the number of atoms (N), the box volume (V) and temperature (T) constant (NVT equilibration), and a subsequent 1-ns equilibration run to adjust the pressure (p) (NpT equilibration). Here, restraints on the protein non-hydrogen atoms were applied with a force constant of 10 kJ mol^{-1} $Å^{-2}$ to allow the water molecules to relax around the solutes. We used the velocity rescaling thermostat during the NVT simulations, whereas the Nosé-Hoover thermostat (Nosé, 1984; Hoover, 1985) and the isotropic Parrinello-Rahman barostat (Parrinello and Rahman, 1981) were employed for the NpT simulations. For calculation of the electrostatic interactions with periodic boundary conditions, the particle mesh-Ewald (PME) sum method (Darden et al., 1993; Essmann et al., 1995) was employed, with a cutoff value of 12 Å applied to the short-

range interactions calculated in real space. The Lennard-Jones (LJ) interactions were also cut at 12 Å. The LINCS algorithm (Hess et al., 1997) was employed for constraining all bond lengths during the MD simulations. The time step for integration was set to 4 fs by using virtual sites for the hydrogens (CH_3 groups), The production MD runs were performed for 500 ns for the homology model 1 of both protein forms, using the same MD settings as for the *NpT* equilibration runs yet without restraints on the proteins. The coordinates and velocities were recorded every 20 ps.

Molecular docking and MD simulations of PATL2-α-Toc (Step II):

In order to predict where the α-tocopherol can bind at both protein forms (PATL2-CTN-SEC14-GOLD and PATL2-CTN-SEC14), we performed molecular docking. To do this, we used AutoDockTools (Goodsell et al., 1996; Santos-Martins et al., 2014) to prepare the *pdbqt* files for the protein as receptor and α-tocopherol as ligand, and also defined the grids in which the α-tocopherol should be docked, the so-called docking areas. For either protein, the homology model 1 and also the first three conformational clusters obtained from the preceding MD simulations were used for docking, as common in the ensemble docking approach (Amaro et al., 2018). The α-tocopherol structure was first prepared with Avogadro (Avogadro, 2012; Hanwell et al., 2012) with a slight geometry optimizing to get a reasonable *mol2* file, which was then converted then to the *pdbqt* format. For the grids, three different docking areas were used: (1) the lipid binding site (LBS) behind the anchor and gate helix, (2) the whole SEC14 domain (SBS) to test for alternative binding modes, and (3) the GOLD domain (GBS) in the PATL2-CTN-SEC14-GOLD system to assess the involvement of that domain in α-tocopherol binding. Then, we applied molecular docking (using the *pdbqt* files) via AutoDock Vina (Trott and Olson, 2010),which treats ligands as fully flexible, while keeping the receptor rigid. In the ensemble docking approach used here, receptor flexibility is considered by using different protein conformations for the receptor. We used the following AutoDock Vina settings: (1) exhaustiveness of 8 for a longer and detailed searching of the α-tocopherol binding modes and (2) number of maximal α- tocopherol binding modes set to five to ensure enough sampling for the different binding sites.

Each of the five α- tocopherol conformations in the different binding sites is scored by a binding energy (ΔG), which indicates how preferable the PATL2-α-tocopherol interactions are. A positive value means that the binding mode is not preferred and involves unfavorable contacts, while negative values represent favorite binding modes. For each binding site in the different docking areas we selected at least one favorable α-tocopherol binding mode (the one with lowest ΔG per binding site) for further MD simulations. The list with the selected binding modes can be found in Supplemental Table S4. In total, we chose 17 and 10 binding modes for the PATL2-CTN-SEC14-GOLD and PATL2-CTN-SEC14, respectively, and simulated each of

them for 100 ns to further assess their stability. The same MD parameters were used as already described above, except that we constrained only the bonds involving hydrogen atoms (and not all bonds) with the LINCS algorithm. For α-tocopherol, we obtained the force field parameters of the Feller group, which were previously used by (Leng et al., 2015; Leng et al., 2018), and converted them into the required input format for MD simulations with GROMACS.

Analysis of the simulations

To generate the 3D protein structure figures, we used PyMol (PyMol, 2015). If not stated otherwise, all analyses were performed with GROMACS 2020 (Abraham et al., 2015; Lindahl et al., 2021). For describing the protein flexibility during the MD simulations, the root mean square fluctuation (RMSF) of the Cα atoms around their average positions was calculated for each residue. The RMSF values represent the positional changes of the selected atoms as time averages, where residues with RMSF values over 2 Å were defined as flexible. Furthermore, we used the evolution of the root mean square deviation (RMSD) of the protein conformations sampled during the MD simulations with respect to the corresponding homology model (model 1 of PATL2-CTN-SEC14-GOLD and PATL2-CTN-SEC14, respectively). Again, the Cα atoms were used for that calculation and the RMSDs of the whole proteins (for overall movements) and of the individual domains (for internal changes) were determined, which was accomplished by fitting to the respective parts of the starting structure. A clustering analysis was performed to identify the preferred protein conformations. To this end, the Daura algorithm (Daura et al., 1999) was employed and applied to the Cα atoms. A cutoff value of 3 Å based on the RMSD between the conformations was used to separate the conformations into clusters. The three most populated clusters were considered for the docking of α-tocopherol.

In order to validate the PATL2-α-tocopherol binding modes studied by MD simulations, the RMSD of α-tocopherol ($RMSD_{\alpha\text{-Toc}}$) was calculated after fitting the trajectory onto the protein structure as present at the beginning of the simulation. The other quantity that was calculated was the distance between the centers of mass of the binding site in question and α-tocopherol ($DIST_{COM}$). For α-tocopherol considered to be stably bound, two criteria had to be fulfilled: (1) a trajectory-averaged $RMSD_{\alpha\text{-Toc}} \leq 5$ Å and (2) a trajectory-average $DIST_{COM} \leq 8$ Å. The mean value and standard deviation (SD) for the two criteria ($DIST_{COM}$, $RMSD_{\alpha\text{-Toc}}$), were calculated over 100 ns MD simulations for PATL2-α-tocopherol binding modes by using n= 10,000 datapoints. To characterize the interactions between PATL2 and α-tocopherol, we used Ligplot+ (Wallace et al., 1995; Laskowski and Swindells, 2011) 2D plot. To identify the best binding modes, we performed a cluster analysis of the α-tocopherol conformations of the respective trajectory after aligning the protein conformations of that trajectory. An $RMSD_{\alpha\text{-Toc}}$ cutoff of 2 Å was used to separate the α-tocopherol conformations into clusters.

References

Abraham MJ, Murtola T, Schulz R, Páll S, Smith JC, Hess B, Lindahl E (2015) GROMACS: High performance molecular simulations through multi-level parallelism from laptops to supercomputers. SoftwareX **1-2:** 19-25

Amaro RE, Baudry J, Chodera J, Demir Ö, McCammon JA, Miao Y, Smith JC (2018) Ensemble Docking in Drug Discovery. Biophys J **114:** 2271-2278

Avogadro (2012) Avogadro: an open-source molecular builder and visualization tool. *In* https://avogadro.cc, ed,

Ben Abdallah H, Bauer P (2016) Quantitative Reverse Transcription-qPCR-Based Gene Expression Analysis in Plants. Methods in Molecular Biology **1363:** 9-24

Bensmihen S, To A, Lambert G, Kroj T, Giraudat J, Parcy F (2004) Analysis of an activated ABI5 allele using a new selection method for transgenic Arabidopsis seeds. FEBS Lett **561:** 127-131

Brumbarova T, Le CTT, Bauer P (2016) Hydrogen Peroxide Measurement in Arabidopsis Root Tissue Using Amplex Red. Bio-protocol **6:** e1999

Caesar K, Elgass K, Chen Z, Huppenberger P, Witthöft J, Schleifenbaum F, Blatt MR, Oecking C, Harter K (2011) A fast brassinolide-regulated response pathway in the plasma membrane of Arabidopsis thaliana. Plant J **66:** 528-540

Clough SJ, Bent AF (1998) Floral dip: a simplified method for Agrobacterium-mediated transformation of Arabidopsis thaliana. Plant J **16:** 735-743

Curtis MD, Grossniklaus U (2003) A gateway cloning vector set for high-throughput functional analysis of genes in planta. Plant Physiol **133:** 462-469

Darden T, York D, Pedersen L (1993) Particle mesh Ewald: An N·log(N) method for Ewald sums in large systems. The Journal of Chemical Physics **98:** 10089-10092

Daura X, Gademann K, Jaun B, Seebach D, van Gunsteren WF, Mark AE (1999) Peptide folding: When simulation meets experiment. Angewandte Chemie-International Edition **38:** 236-240

Eide D, Broderius M, Fett J, Guerinot ML (1996) A novel iron-regulated metal transporter from plants identified by functional expression in yeast. Proc. Natl. Acad. Sci. USA **93:** 5624-5628

Essmann U, Perera L, Berkowitz M, Darden T, Lee H, Pedersen L (1995) A Smooth Particle Mesh Ewald Method. J. Chem. Phys. **103:** 8577

Goodsell DS, Morris GM, Olson AJ (1996) Automated docking of flexible ligands: applications of AutoDock. J Mol Recognit **9:** 1-5

Gratz R, Manishankar P, Ivanov R, Koster P, Mohr I, Trofimov K, Steinhorst L, Meiser J, Mai HJ, Drerup M, Arendt S, Holtkamp M, Karst U, Kudla J, Bauer P, Brumbarova T (2019) CIPK11-Dependent Phosphorylation Modulates FIT Activity to Promote Arabidopsis Iron Acquisition in Response to Calcium Signaling. Developmental Cell **48:** 726-+

Grefen C, Blatt MR (2012) A 2in1 cloning system enables ratiometric bimolecular fluorescence complementation (rBiFC). Biotechniques **53:** 311-314

Grube L, Dellen R, Kruse F, Schwender H, Stühler K, Poschmann G (2018) Mining the Secretome of C2C12 Muscle Cells: Data Dependent Experimental Approach To Analyze Protein Secretion Using Label-Free Quantification and Peptide Based Analysis. J Proteome Res **17:** 879-890

Hanwell MD, Curtis DE, Lonie DC, Vandermeersch T, Zurek E, Hutchison GR (2012) Avogadro: an advanced semantic chemical editor, visualization, and analysis platform. Journal of Cheminformatics **4:** 17

Havaux M, Eymery F, Porfirova S, Rey P, Dörmann P (2005) Vitamin E protects against photoinhibition and photooxidative stress in Arabidopsis thaliana. Plant Cell **17:** 3451-3469

Hess B, Bekker H, Berendsen HJC, Fraaije JGEM (1997) LINCS: A linear constraint solver for molecular simulations. Journal of Computational Chemistry **18:** 1463-1472

Hoover WG (1985) Canonical dynamics: Equilibrium phase-space distributions. Physical Review A **31:** 1695-1697

Ivanov R, Brumbarova T, Blum A, Jantke AM, Fink-Straube C, Bauer P (2014) SORTING NEXIN1 is required for modulating the trafficking and stability of the Arabidopsis IRON-REGULATED TRANSPORTER1. Plant Cell **26:** 1294-1307

Jarmoskaite I, AlSadhan I, Vaidyanathan PP, Herschlag D (2020) How to measure and evaluate binding affinities. eLife **9:** e57264

Jefferson RA, Kavanagh TA, Bevan MW (1987) GUS fusions: beta-glucuronidase as a sensitive and versatile gene fusion marker in higher plants. EMBO J **6:** 3901-3907

Jurrus E, Engel D, Star K, Monson K, Brandi J, Felberg LE, Brookes DH, Wilson L, Chen J, Liles K, Chun M, Li P, Gohara DW, Dolinsky T, Konecny R, Koes DR, Nielsen JE, Head-Gordon T, Geng W, Krasny R, Wei GW, Holst MJ, McCammon JA, Baker NA (2018) Improvements to the APBS biomolecular solvation software suite. Protein Sci **27:** 112-128

Kar S, Mai HJ, Khalouf H, Abdallah HB, Flachbart S, Fink-Straube C, Bräutigam A, Xiong G, Shang L, Panda SK, Bauer P (2021) Comparative transcriptomics of lowland rice varieties leads to novel candidate genes for adaptive iron excess tolerance. Plant Cell Physiol **62:** 624-640

Khan I, Gratz R, Denezhkin P, Schott-Verdugo SN, Angrand K, Genders L, Basgaran RM, Fink-Straube C, Brumbarova T, Gohlke H, Bauer P, Ivanov R (2019) Calcium-Promoted Interaction between the C2-Domain Protein EHB1 and Metal Transporter IRT1 Inhibits Arabidopsis Iron Acquisition. Plant Physiol **180:** 1564-1581

Laskowski RA, Swindells MB (2011) LigPlot+: multiple ligand-protein interaction diagrams for drug discovery. J Chem Inf Model **51:** 2778-2786

Le CTT, Brumbarova T, Ivanov R, Stoof C, Weber E, Mohrbacher J, Fink-Straube C, Bauer P (2016) ZINC FINGER OF ARABIDOPSIS THALIANA12 (ZAT12) Interacts with FER-LIKE IRON DEFICIENCY-INDUCED TRANSCRIPTION FACTOR (FIT) Linking Iron Deficiency and Oxidative Stress Responses. Plant Physiol **170:** 540-557

Leng X, Kinnun JJ, Marquardt D, Ghefli M, Kučerka N, Katsaras J, Atkinson J, Harroun TA, Feller SE, Wassall SR (2015) α-Tocopherol Is Well Designed to Protect Polyunsaturated Phospholipids: MD Simulations. Biophysical journal **109:** 1608-1618

Leng X, Zhu F, Wassall SR (2018) Vitamin E Has Reduced Affinity for a Polyunsaturated Phospholipid: An Umbrella Sampling Molecular Dynamics Simulations Study. The Journal of Physical Chemistry B **122:** 8351-8358

Lindahl, Abraham, Hess, Spoel vd (2021) GROMACS 2020.5 Source code (2020.5). *In*,

Lyska D, Engelmann K, Meierhoff K, Westhoff P (2013) pAUL: a gateway-based vector system for adaptive expression and flexible tagging of proteins in Arabidopsis. PLoS One **8:** e53787

Mi H, Ebert D, Muruganujan A, Mills C, Albou LP, Mushayamaha T, Thomas PD (2021) PANTHER version 16: a revised family classification, tree-based classification tool, enhancer regions and extensive API. Nucleic Acids Res **49:** D394-d403

Montag K, Hornbergs J, Ivanov R, Bauer P (2020) Phylogenetic analysis of plant multi-domain SEC14-like phosphatidylinositol transfer proteins and structure–function properties of PATELLIN2. Plant Molecular Biology **104:** 665-678

Naranjo Arcos MA, Maurer F, Meiser J, Pateyron S, Fink-Straube C, Bauer P (2017) Dissection of iron signaling and iron accumulation by overexpression of subgroup Ib bHLH039 protein. Scientific Rep **7:** 10911

Nava P, Cecchini M, Chirico S, Gordon H, Morley S, Manor D, Atkinson J (2006) Preparation of fluorescent tocopherols for use in protein binding and localization with the α-tocopherol transfer protein. Bioorganic & Medicinal Chemistry **14:** 3721-3736

Nosé S (1984) A molecular dynamics method for simulations in the canonical ensemble. Molecular Physics **52:** 255-268

Parrinello M, Rahman A (1981) Polymorphic transitions in single crystals: A new molecular dynamics method. Journal of Applied Physics **52:** 7182-7190

PyMol (2015) The pymol molecular graphics system, version 1.8. *In* Schrödinger, ed. LLC

Roy A, Kucukural A, Zhang Y (2010) I-TASSER: a unified platform for automated protein structure and function prediction. Nat Protoc **5:** 725-738

Santos-Martins D, Forli S, Ramos MJ, Olson AJ (2014) AutoDock4(Zn): an improved AutoDock force field for small-molecule docking to zinc metalloproteins. J Chem Inf Model **54:** 2371-2379

Stahl E, Hartmann M, Scholten N, Zeier J (2019) A Role for Tocopherol Biosynthesis in Arabidopsis Basal Immunity to Bacterial Infection. Plant Physiol **181:** 1008-1028

Trott O, Olson AJ (2010) AutoDock Vina: improving the speed and accuracy of docking with a new scoring function, efficient optimization, and multithreading. Journal of computational chemistry **31:** 455-461

Vert G, Grotz N, Dedaldechamp F, Gaymard F, Guerinot ML, Briat JF, Curie C (2002) IRT1, an Arabidopsis transporter essential for iron uptake from the soil and for plant growth. Plant Cell **14:** 1223-1233

Wallace AC, Laskowski RA, Thornton JM (1995) LIGPLOT: a program to generate schematic diagrams of protein-ligand interactions. Protein Eng **8:** 127-134

Yang J, Yan R, Roy A, Xu D, Poisson J, Zhang Y (2015) The I-TASSER Suite: protein structure and function prediction. Nat Methods **12:** 7-8

Zhang Y (2008) I-TASSER server for protein 3D structure prediction. BMC Bioinformatics **9:** 40

Zhang Z, Huang R (2013) Analysis of Malondialdehyde, Chlorophyll Proline, Soluble Sugar, and Glutathione Content in Arabidopsis seedling. BIO-PROTOCOL **3**

Figure 1B:

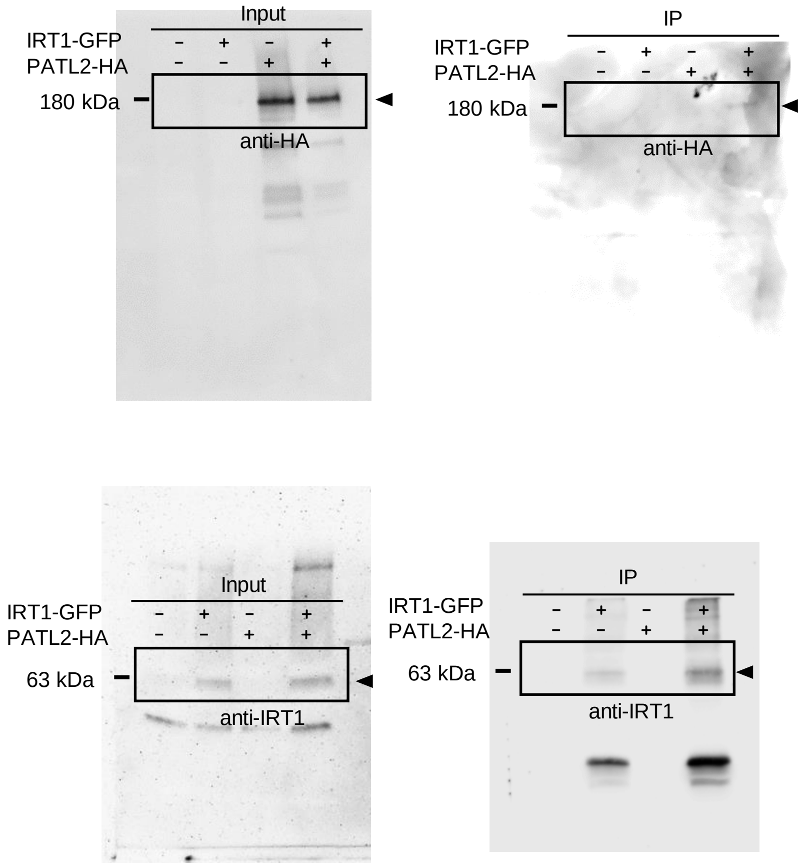

Figure 2B:

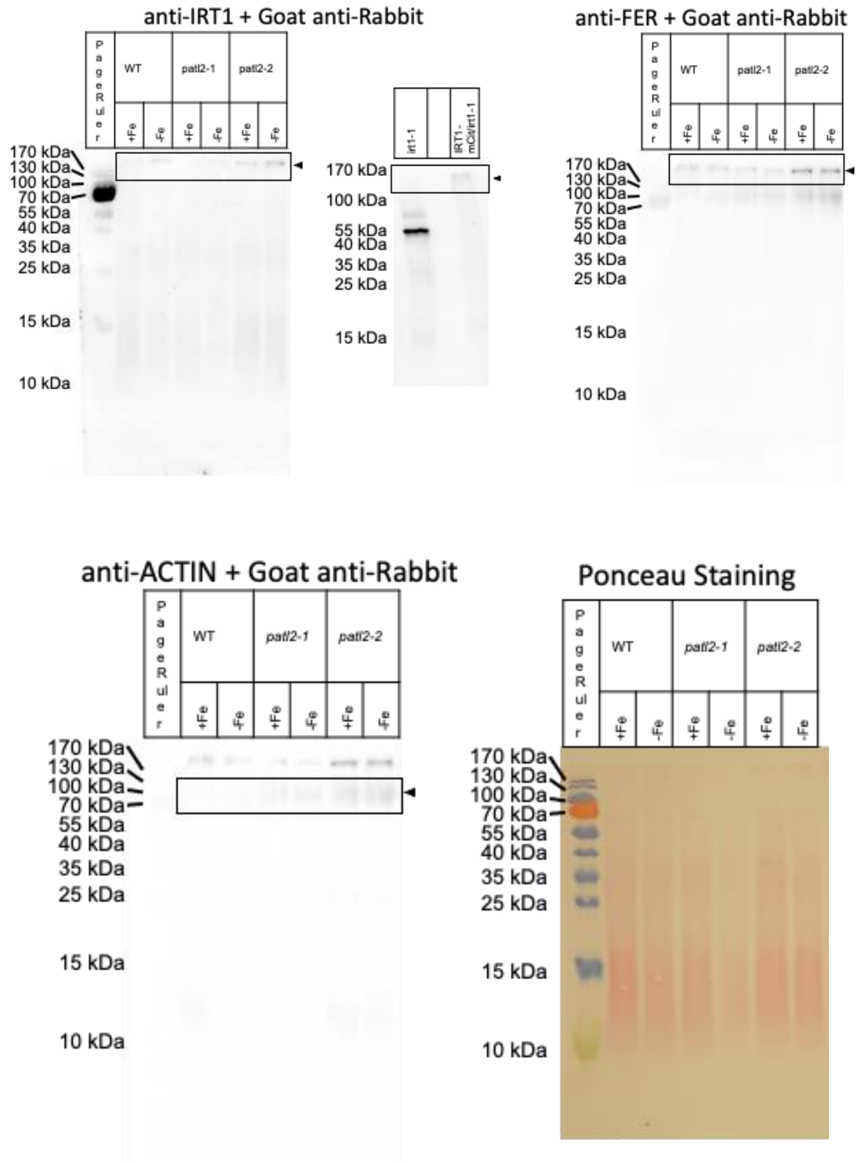

Supplement: kiac563_Supplementary_Data [file kiac563_supplementary_data.zip › Supplemental file Mat Methods.pdf]
