# Supplementary material for: SEC14-GOLD protein PATELLIN2 binds IRON-REGULATED TRANSPORTER1 linking root iron uptake to vitamin E
Source: Plant Physiol. 2022 Dec 9;192(1):504–26. doi: 10.1093/plphys/kiac563 (PMC10152663; doi:10.1093/plphys/kiac563)
Supplement: kiac563_Supplementary_Data [file kiac563_supplementary_data.zip › Supplemental Table S4.pdf]

Supplemental Table S4: Summary of molecular simulations performed in this work.

System	Size in atoms	Runs
PATL2-CTN-SEC14-GOLD	130,581	1 × 500 ns
PATL2-CTN-SEC14	72,319	1 × 500 ns
PATL2-CTN-SEC14-GOLD with α-tocopherol	~130,000	17 × 100 ns
PATL2-CTN-SEC14 with α-tocopherol	~71,000	10 × 100 ns
Total simulation time		3700 ns

CRAL-TRIO-N-terminal extension, CTN; Golgi dynamics, GOLD; SEC14, SEC14.
